# Atom-efficient synthesis of 2,4,6-trisubstituted 1,3,5-triazines *via* Fe-catalyzed cyclization of aldehydes with NH_4_I as the sole nitrogen source†

**DOI:** 10.1039/d0ra03323e

**Published:** 2020-06-09

**Authors:** Jiang Xiao, Shuang Ren, Qiang Liu

**Affiliations:** State Key Laboratory of Chemo/Biosensing and Chemometrics, College of Chemistry and Chemical Engineering, Hunan University Changsha 410082 P.R. China fqiangliu@qq.com ccguo@hnu.edu.cn

## Abstract

An atom-efficient, straightforward method for the synthesis of 2,4,6-triaryl-1,3,5-triazines *via* iron-catalyzed cyclization of aldehydes with NH_4_I as the sole nitrogen source is demonstrated. This strategy works smoothly under air atmosphere, and affords symmetrical 2,4,6-trisubstituted and unsymmetrical 1,3,5-triazines with yields from 18% to 72%. Compared to other methods, the present protocol provides a straightforward and atom-efficient approach to 2,4,6-trisubstituted 1,3,5-triazines using an inexpensive, easily available ammonium salt as the sole nitrogen source. Research into the preliminary mechanism indicates that *N*-benzylidenebenzimidamides are involved in this cyclization reaction.

## Abstract 2

An atom-efficient, straightforward method for the synthesis of 2,4,6-triaryl-1,3,5-triazines *via* iron-catalyzed cyclization of aldehydes with NH_4_I as the sole nitrogen source is demonstrated.

## Graphical abstract

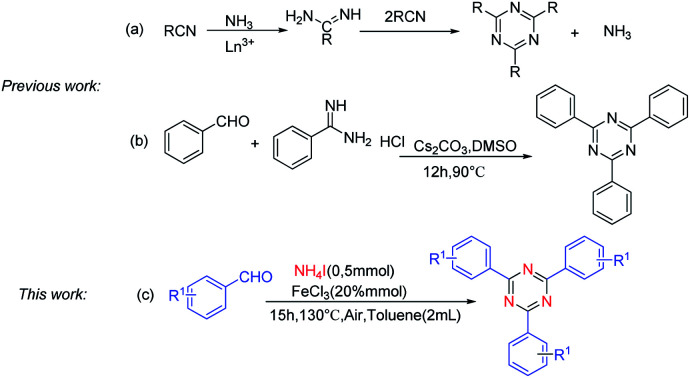

## Introduction

**Scheme 1:**
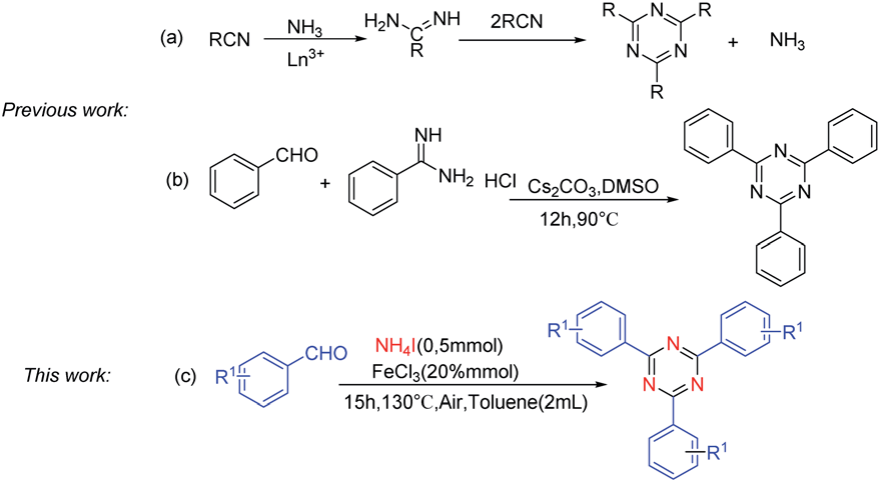
Methods for the synthesis of 1,3,5-triazine derivatives.

The derivatives of 1,3,5-triazine are well-known compounds of considerable interest because of their applications in many different fields.^1^ Actually, these compounds serve as pharmaceuticals,^2^ liquid crystals,^3^ transition-metal catalysts,^4^ building blocks for supramolecular chemistry,^5^ reactive dyes^6^^,^ organic light-emitting diodes (OLEDs),^7^ and chemical reagents for selected transformations.^8^ Although the substituted 1,3,5-triazines have extensive applications, the method of synthesis of these compounds is still very limited. Traditional methods of substituted 1,3,5-triazines preparation involved cyclotrimerization of nitriles,^9^ or cyclization of imidates^10^ and amidine derivatives,^11^ in which nitriles, imidates and amidine^12^ served as nitrogen sources. For example, Forsberg and co-workers found that lanthanum and yttrium trifluoromethanesulfonates could catalyze a reaction between ammonia salts and aromatic nitriles to yield symmetrically 2,4,6- substituted 1,3,5-triazine^13^ (Scheme 1a). However, ammonia salts were used as cocatalysts rather than a nitrogen source. Condensation of aromatic aldehydes with amidines was found to produce symmetrically and unsymmetrically 2,4,6- substituted 1,3,5-triazine^14^ (Scheme 1b), in which, aromatic amidines were the nitrogen source. Recently, the coupling of halogenated 1,3,5-triazines and aryl boronic acids based on Suzuki-coupling reactions has been explored.^15^ Oxidative coupling reaction of amidine hydrochlorides and alcohol has been developed to synthesize 1,3,5-triazine derivatives.^16^ In this procedure, amidine was the nitrogen sources. Therefore, it is remarkable significance to find a cheap, readily available nitrogen source for 1,3,5-triazine synthesis.

Ammonium iodide (NH_4_I) as nitrogen source was widely used to synthesis nitrogen-containing heterocycles and nitrile. For instance, Deng and co-works reported NH_4_I as a nitrogen source to construct pyrimidines and pyridines.^17^ Guo and co-works reported cyanation of ketones using ammonium salts as the nitrogen source in the presence of TBAI.^18^ In continuation with our ongoing work on cyclization approaches in organic synthesis,^19^ herein, we demonstrate that NH_4_I can be used as the sole nitrogen source for the cyclotrimerization of aldehydes (Scheme 1c).

## Results and discussion

Initially, we investigated the reaction between benzaldehyde (1a) and NH_4_I under the condition of air and chlorobenzene as the solvent. It was found that chlorobenzene is a good solvent for high yield synthesis of nitrogen-containing heterocyclic compounds using NH_4_I as the nitrogen source.^20^^.^Therefore, we chose chlorobenzene as the initial solvent, and obtained 62% yield of 2a with adding 0.2 equiv. of FeCl_3_ (Table 1, entries 1). Moderate yields of 2a were observed in the presence of other catalysts, such as CuCl_2_, CoCl_2_ and CuO (Table 1, entries 2–4). This result encouraged us to choose FeCl_3_ as the catalyst. Next, reaction temperature was scanned to improve the yield, 130 °C was determined as optimum for the cyclotrimerization of aldehydes (Table 1, entries 6). Finally, the screening of solvents was carried out, toluene was found to be the most suitable medium for this cyclotrimerization reaction compared with chlorobenzene, DMF, and DMSO (Table 1, entries 6, 8–10). After screening on different parameters, the highest yield of 2a (72%) was achieved^2*a*^ when the reaction was carried out with benzaldehyde (0.5 mmol), NH_4_I (0.5 mmol), FeCl_3_ (20% mmol) at 130 °C under the atmosphere of air in toluene (entry 8; Table 1).

**Table 1:** Optimization of reaction conditions^a^

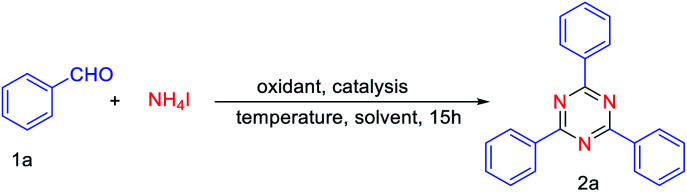				
Entry	Catalysis	Temperature [°C]	Solvent	Yield^b^
1	FeCl_3_	150	Chlorobenzene	62%
2	CuCl_2_	150	Chlorobenzene	53%
3	CoCl_2_	150	Chlorobenzene	49%
4	CuO	150	Chlorobenzene	32%
5	FeCl_3_	140	Chlorobenzene	61%
6	FeCl_3_	130	Chlorobenzene	60%
7	FeCl_3_	120	Chlorobenzene	32%^d^
**8**	**FeCl_3_**	**130**	**Toluene**	**72%**
9	FeCl_3_	130	DMSO	—
10	FeCl_3_	130	DMF^c^	—

a Reaction conditions: benzaldehyde 1a (0.5 mmol), NH_4_I (0.5 mmol), catalysis (20% mmol), solvent (2.0 mL).

b Yield calculated by GC-MS.

c DMF = dimethylformamide.

d 24 h.

With the optimized reaction conditions in hand, the substrate scope of aldehyde was firstly evaluated for the synthesis of various symmetrical 2,4,6-trisubstituted 1,3,5-triazines (Table 2). A series of electron-donating or electron-withdrawing group-substituted benzaldehyde could be employed in this reaction (Table 2, 2b–2q), affording the desire 2,4,6-trisubstituted 1,3,5-triazines in 41–72% yields. Among them, the yield of aldehyde bearing electron-donating group such as –Me, –OMe (Table 2, 2b, 2c, 2m, 2n) has been found no significant difference compared with that of aldehyde bearing electron-withdrawing ones such as –F, –Cl,–Br (Table 2, 2j, 2k, 2l). Both of them provided the desired products in good yields. Moreover, the steric effect of substituted benzaldehydes was also explored. Reaction of *para* and *meta*-subsitituted benzaldehyde (Table 2, 2b, 2c, 2k) with NH_4_I gave the corresponding products in higher yields compared with *ortho*-substituted ones (Table 2, 2d, 2h). Unfortunately, when disubstituted benzaldehyde and trisubstituted benzaldehyde were used as substrates, the lower yield of corresponding products was obtained (Table 2, 2o, 2r). When heteroaryl carbaldehydes were used as substrates (Table 2, 2e, 2f), the desired products also produced in 64% and 65% yields.

**Table 2:** Scope of various aldehyde and NH_4_I^a^

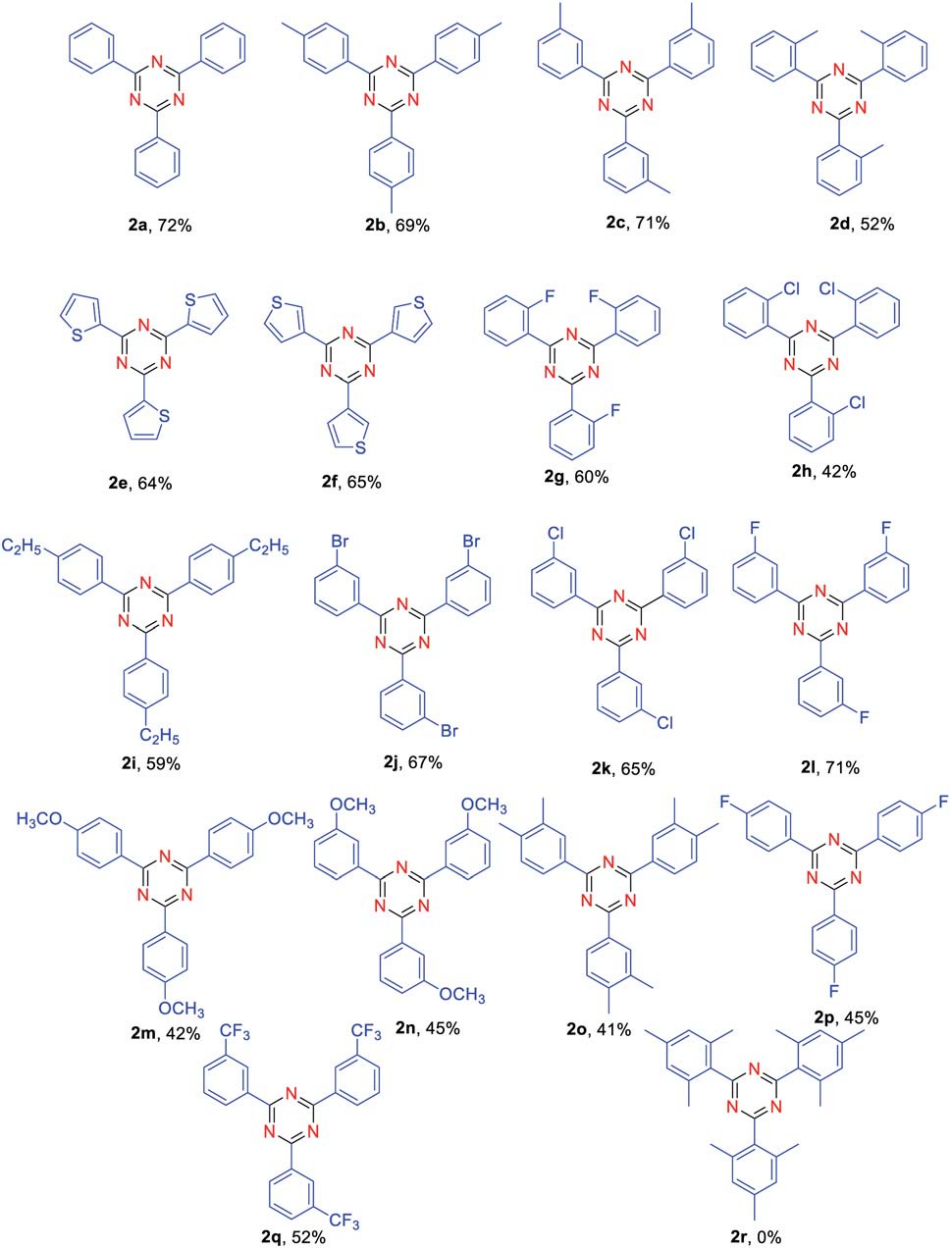

a Reaction conditions: aldehyde (0.5 mmol), NH_4_I 2 (0.5 mmol), FeCl_3_ (20% mmol), toluene (2.0 mL) were heated at 130 °C for 15 h.

Synthesis of unsymmetrical 2,4,6-trisubstituted 1,3,5- triazines was also explored. To our delight, the reaction proceeded very well for benzaldehyde and 4-methylbenzaldehyde as substrates, providing the corresponding products in moderate yields (Table 3, 3h, 4h). Moreover, the reactions of benzaldehyde and thiophene-2-carbaldehyde or thiophene-3-carbaldehyde were also found to be effective (Table 3, 3b, 4b, 4c). However, the reaction of 3-methylbenzaldehyde and thiophene-2-carbaldehyde gave a relatively low yield compared with benzaldehyde and thiophene-2-carbaldehyde as substances (Table 3, 3f). Notably, when *ortho*-substituted aldehydes were used to react with another aldehyde, no desired product could be obtained. It is confirmed that the steric effect is critical for the reaction.

**Table 3:** Scope of different aldehyde and NH_4_I^a^

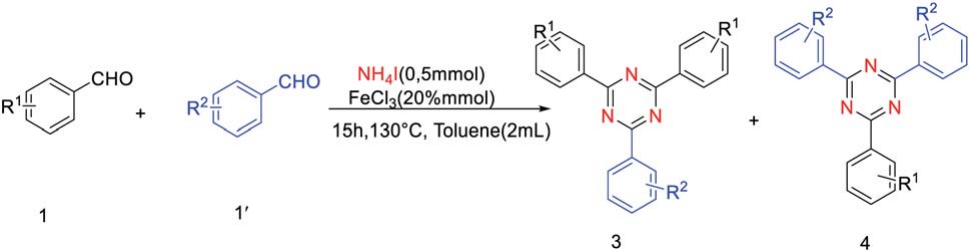
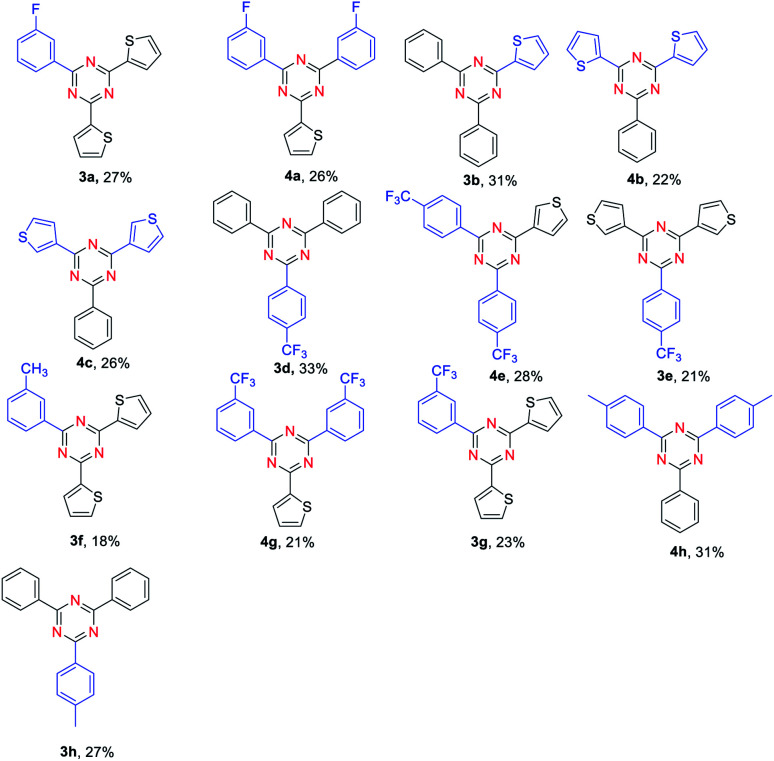

a Reaction conditions: aldehyde 1 (0.3 mmol), aldehyde 1, (0.2 mmol), NH_4_I (0.5 mmol), FeCl_3_ (20% mmol), toluene (2.0 mL) were heated at 130 °C for 15 h.

**Scheme 2:**
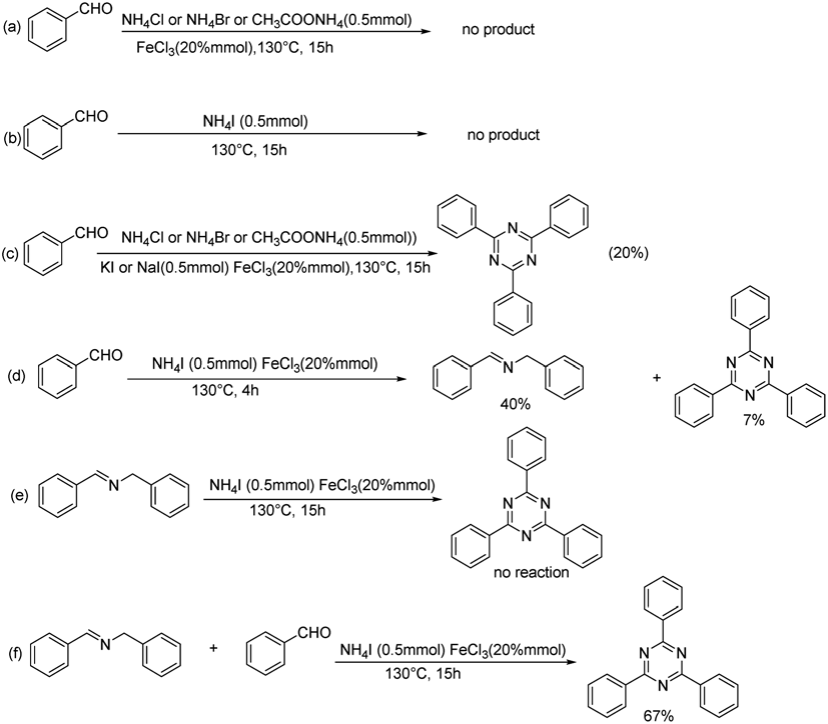
Control experiments.

In order to gain insight into the reaction mechanism, a couple of control experiments were performed (Scheme 2). Firstly, when a variety of ammonium salts other than NH_4_I were heated in toluene at 130 °C for 15 h, no product was observed (Scheme 2a). With NH_4_I as nitrogen source, the desired product was similarly not observed without addition of FeCl_3_ (Scheme 2b). Reaction of benzaldehyde and other ammonium salts also offered corresponding products with addition of KI or NaI (Scheme 2c). Next, the model reaction was detected by GC-Ms for 1 h up to 10 h. The *N*-benzyl-1-phenylmethanimine was the main product (40%) at 4 h (Scheme 2d). Afterward, we used *N*-benzyl-1-phenylmethanimine as substance to determine whether it participated in the reaction under standard conditions. It was found that *N*-benzyl-1-phenylmethanimine still existed after reaction (Scheme 2e). We then used *N*-benzyl-1-phenylmethanimine and benzaldehyde as substances (Scheme 2f), the desired product was obtained with a yield of 67%. This yield is similar with that of the reaction with only aldehyde as substance. To our surprising, a large amount of *N*-benzyl-1-phenylmethanimine maintained after detection of GC-MS (ESI Fig. 1†). Therefore, *N*-benzyl-1-phenylmethanimine may be not an intermediate of the reaction.

**Scheme 3:**
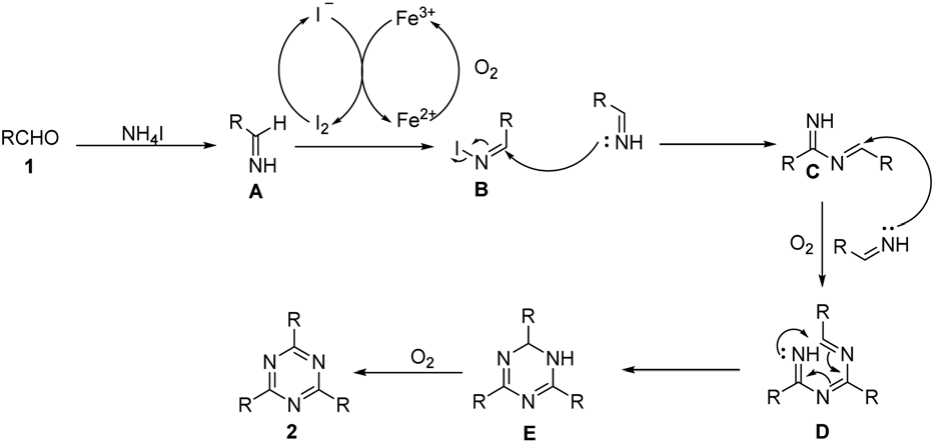
Proposed reaction mechanism.

Based on control experiments and literature results,^21,22^ a plausible mechanism is depicted in Scheme 3. Firstly, aldehydes and NH_4_I react to form imines A. Meanwhile, Fe^3+^ oxidizes I^−^ to form I_2_, which then oxidizes the imine intermediate to obtain *N*-iodo aldimine intermediate B.^21^ The condensation reaction of imines A and intermediate B affords an imine intermediate C. Subsequently, under the oxidation of oxygen, the cyclization reaction of imines A and intermediate C yields intermediate E by two steps. Finally, the intermediate E undergoes an oxidation reaction to offer the desired product 1,3,5-triazine (2).

## Conclusions

In summary, we have developed a Fe-catalyzed cyclization of aldehydes with NH_4_I for the synthesis of symmetrical and unsymmetrical 2,4,6-trisubstituted 1,3,5-triazines in moderate to good yields. This method has relatively broad substrate scope. Importantly, NH_4_I was employed as sole nitrogen resource. This protocol provides a simple and atom-efficient route to synthesize valuable 1,3,5-triazines.

## Conflicts of interest

There are no conflicts to declare.

## Supplementary Material

RA-010-D0RA03323E-s001
