# Supplementary material for: Atom-efficient synthesis of 2,4,6-trisubstituted 1,3,5-triazines via Fe-catalyzed cyclization of aldehydes with NH4I as the sole nitrogen source
Source: RSC Adv. 2020 Jun 9;10(37):22230–3. doi: 10.1039/d0ra03323e (PMC9054497; doi:10.1039/d0ra03323e)
Supplement: RA-010-D0RA03323E-s001 [file RA-010-D0RA03323E-s001.pdf]

Electronic Supplementary Material (ESI) for RSC Advances.
This journal is © The Royal Society of Chemistry 2020

Supporting Information

Table of Contents

1. **General information** S2
2. **General procedures**....S2-S3
3. **GC-MS detection of reaction**....S4
4. **Spectra Data of the Products** S5
5. **NMR data of products** S5-S13
6. **Notes and references**.... S14

1. General information

Unless otherwise noted, all reactions were carried out in Schlenk tubes. Reagents and solvents were obtained from commercial sources and used without further purification. The ^{1}H ,^{13}C and ^{9}F spectra were recorded on a Brucker ADVANCE III spectrometer at 400 MHz, 100 MHz and 376 MHz, and chemical shifts were reported in parts per million (ppm). Column chromatography was performed using silica gel of 300-400 μm.The GC-MS results were recorded on a GC-MS QP2010 equipmment. The electron ionization (EI) method was used for HRMS measurement, and the mass analyzer type is TOF for EI. The HRMS (EI) was recorded on an Esquire 3000 plus instrument.

2. General Procedures

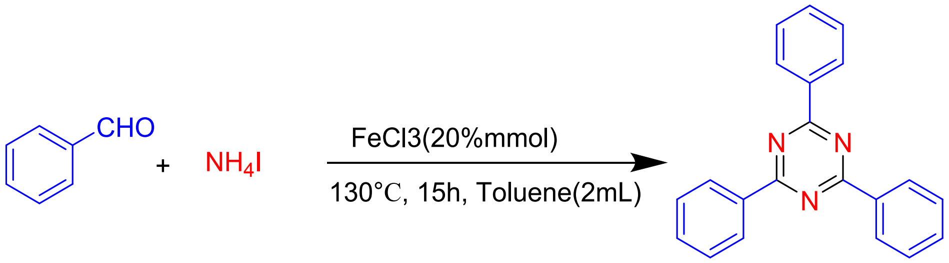

General procedure for the synthesis of symmetrical 2,4,6-trisubstitued1,3,5-triazin: In a Schlenk tube of 25 mL, benzaldehyde 1a (0.5 mmol, 1.0 equiv.), NH_4I 2a (0.5 mmol, 1.0 equiv.), $FeCl_3$ (0.1 mmol, 0.2 equiv.), were dissolved in Toluene (2 mL). The mixture was stirred at 130 °C for 15 h under air atmosphere. After completion of the reaction, the resulting solution was cooled to room temperature; the solution was diluted with ethyl acetate (10 mL), washed with water (5 mL), extracted with ethyl acetate (3×5 mL), dried over anhydrous Na_2SO_4 and concentrated in vacuo. The crude product was purified by column chromatography on silica gel (petroleum ether /diethyl ether) to afford the product in order to analyze NMR.

.

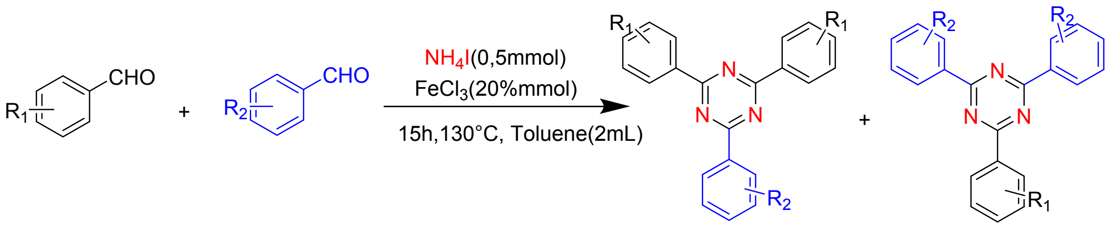

General procedure for the synthesis of unsymmetrical 2,4,6-trisubstitued 1,3,5-triazin: In a Schlenk tube of 25 mL, benzaldehyde 1a (0.3 mmol.), 4-methylbenzaldehyde 1b (0.2mmol.) NH_4I 2a (0.5 mmol.), $FeCl_3$ (0.1 mmol, 0.2 equiv.), were dissolved in Toluene (2 mL). The mixture was stirred at 130 °C for 15 h under air atmosphere. After completion of the reaction, the resulting solution was cooled to room temperature; the solution was diluted with ethyl acetate (10 mL), washed with water (5

mL), extracted with ethyl acetate (3×5 mL), dried over anhydrous Na_2SO_4 and concentrated in vacuo. The crude product was purified by column chromatography on silica gel (petroleum ether /diethyl ether) to afford the product in order to analyze NMR.

3. GC-MS detection of reaction

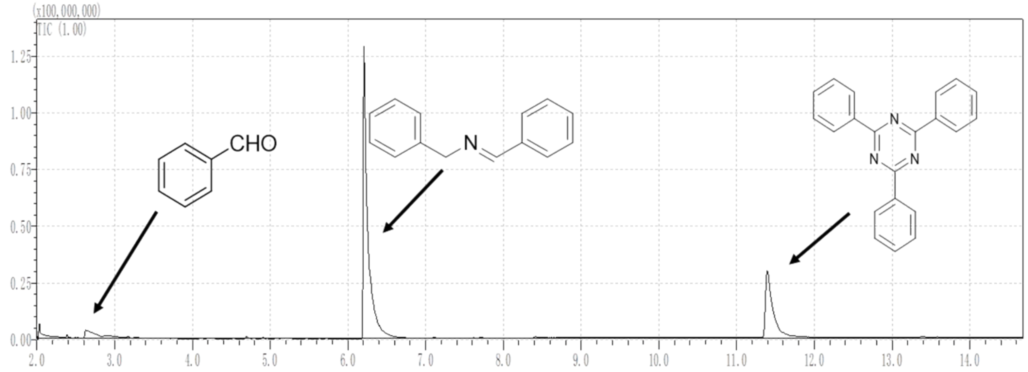

Figure.1 GC-MS detection of aldehyde and N-benzyl-1-phenylmethanimine reacted

4. Spectra Data of the Products

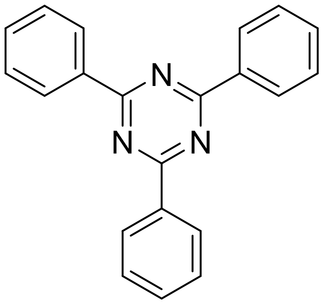

2,4,6-triphenyl-1,3,5-triazine(2a)

White solid; mp: 238.2-239.1 °C; Lit.mp: 237-239 °C [1] **^{1}HNMR** (400 MHz, $CDCl_3$) δ 8.80-8.78 (dd, J = 8.0 Hz, 6H), 8.17- 7.42 (m, 9H). **^{13}C NMR** (100 MHz, $CDCl_3$) δ 171.66, 136.26, 132.49, 128.96, 128.63. **HRMS (EI)**: calcd for $C_{21}H_{15}N_3$: 309.1266; found: 309.1272

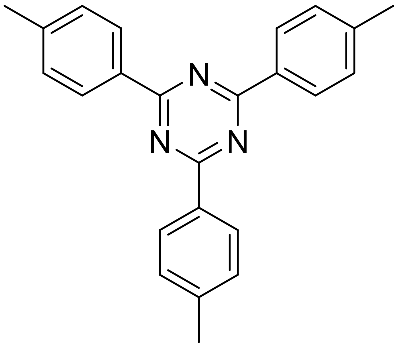

2,4,6-tri-p-tolyl-1,3,5-triazine(2b)

White solid; mp: 292.1-294.4 °C; Lit.mp: 297-298 °C [1]
^{1}H NMR (400 MHz, $CDCl_3$) δ 8.66-8.64 (d, J = 8.0 Hz, 6H), 7.37-7.35 (d, J = 8.0 Hz, 6H),2.48(s, 9H) **^{13}C NMR** (100 MHz, $CDCl_3$) δ 171.37, 142.82, 133.76, 129.30, 128.90, 21.70. **HRMS (EI)**: calcd for $C_{24}H_{21}N_3$: 351.17335; found: 351.1738

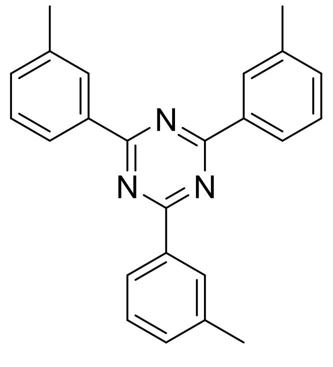

2,4,6-tri-m-tolyl-1,3,5-triazine(2c)

White solid; mp: 145.1-147.6 °C; Lit.mp: 146-147 °C [2] **^{1}H NMR** (400 MHz,$CDCl_3$) δ 8.60-8.58 (d, J = 9.2 Hz, 6H), 7.96 – 6.85 (m, 6H), 2.54（s, 9H） **^{13}C NMR** (100 MHz, $CDCl_3$) δ 171.68, 138.25, 136.27, 133.22, 129.38, 128.50, 126.20, 77.32, 77.00, 76.68, 21.55. **HRMS (EI)**: calcd for $C_{24}H_{21}N_3$: 351.1735; found:351.1742

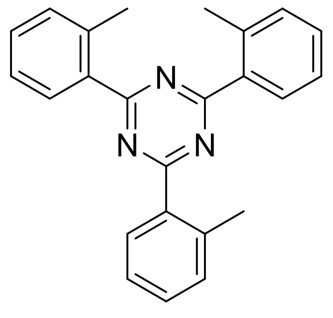

2,4,6-tri-o-tolyl-1,3,5-triazine(2d)

White solid; mp: 112.3-114.7 °C; Lit.mp: 110-111 °C [2] **^{1}HNMR** (400 MHz, $CDCl_3$) δ 8.25-8.23(d, J = 7.6 Hz, 3H), 7.71 – 7.23 (m, 9H), 2.80 (d, J = 13.6 Hz, 9H). **^{13}C NMR** (100 MHz, $CDCl_3$) δ 173.81, 138.91, 136.16, 131.79, 131.28, 130.84, 126.03, 22.26. **HRMS (EI)**: calcd for $C_{24}H_{21}N_3$: 351.1735; found: 351.1731

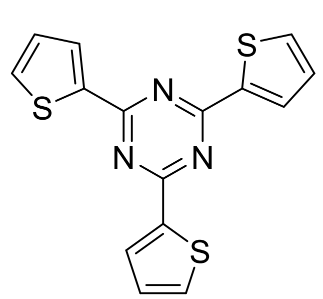

2,4,6-tri(thiophen-2-yl)-1,3,5-triazine(2e)

White solid; mp: 183.6-185.4 °C; Lit.mp: 187 °C [3] **^{1}HNMR** (400 MHz, $CDCl_3$) δ 8.28-8.27 (d, J = 3.6 Hz, 3H), 7.63-7.61 (d, J = 4.8 Hz, 3H), 7.22-7.20 (t, J = 4.4 Hz, 3H). **^{13}C NMR** (100 MHz, $CDCl_3$) δ 167.69, 141.43, 132.28, 131.66, 128.38. **HRMS (EI)**: calcd for $C_{15}H_9N_3S_3$: 326.9959.1263; found: 326.9965

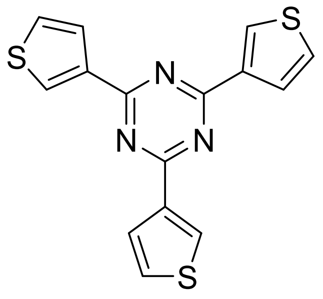

2,4,6-tri(thiophen-3-yl)-1,3,5-triazine(2f)

White solid; mp: 176.4-178.8 °C; **^{1}H NMR** (400 MHz, $CDCl_3$) δ 8.61-8.60 (dd, J = 3.0, 1.0 Hz, 3H), 8.07-8.05 (dd, J = 4.8, 1.2 Hz, 3H), 7.44-7.42 (dd, J = 5.2, 3.2 Hz, 3H). **^{13}C NMR** (100 MHz, $CDCl_3$) δ 168.34, 140.40, 131.00, 127.62, 126.21. **HRMS (EI)**: calcd for $C_{15}H_{19}N_3S_3$: 326.9959; found: 326.9963

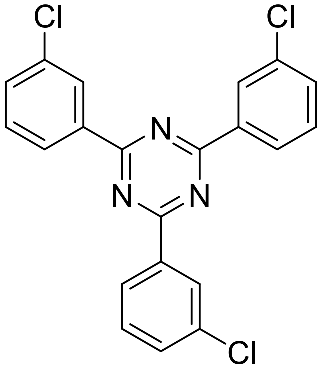

2,4,6-tris(3-chlorophenyl)-1,3,5-triazine(2k)

White solid; mp: 226.2-228.7 °C; **^{1}H NMR** (400 MHz, $CDCl_3$) δ 8.61 (s, 3H), 8.57-8.55 (d, J = 8.0 Hz, 3H), 7.58-7.56 (d, J = 8.0 Hz, 3H), 7.51-7.47 (t, J = 7.8 Hz, 3H). **^{13}C NMR** (100 MHz, $CDCl_3$) δ 170.67, 137.39, 134.92, 132.76, 129.93, 128.85, 127.09. **HRMS (EI)**: calcd for $C_{21}H_{12}N_3Cl_3$: 411.0097; found: 411.0092

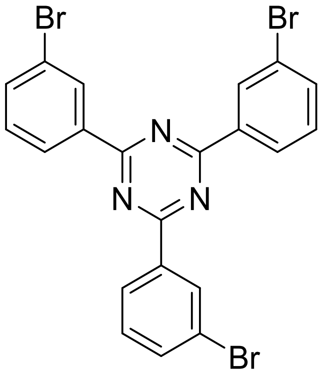

2,4,6-tris(3-bromophenyl)-1,3,5-triazine(2j)

White solid; mp: 192.2-194.5 °C; **^{1}H NMR** (400 MHz, $CDCl_3$) δ 8.76 (s, 3H), 8.61-8.59 (d, J = 8.0 Hz, 3H), 7.74-7.72 (d, J = 7.8 Hz, 3H), 7.44-7.41 (t, J = 8.0 Hz, 3H). **^{13}C NMR** (100 MHz, $CDCl_3$) δ 170.58, 137.59, 135.72, 131.79, 130.21, 127.58, 122.98. **HRMS (EI)**: calcd for $C_{21}H_{12}N_3Br_3$: 544.8561; found: 544.8566

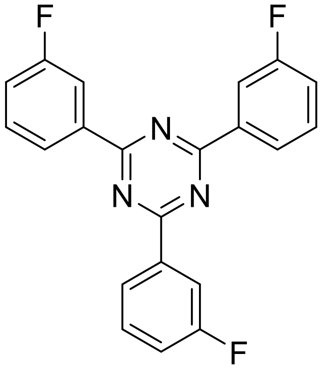

2,4,6-tris(3-fluorophenyl)-1,3,5-triazine(2l)

White solid; mp: 257.4-259.6 °C; **^{1}H NMR** (400 MHz, $CDCl_3$) δ 8.56-8.54 (d, J = 8.0 Hz, 3H), 8.50 – 8.33 (m, 3H), 7.56 (q, J = 7.8 Hz, 3H), 7.46 – 7.27 (m, 3H). **^{13}C NMR** (100 MHz, $CDCl_3$) δ 170.96, 164.39, 161.94, 138.16, 138.08, 130.33, 130.25, 124.70, 124.67, 119.87, 119.66, 115.86, 115.62. **HRMS (EI)**: calcd for $C_{21}H_{12}N_3F_3$: 363.0983; found: 363.0988

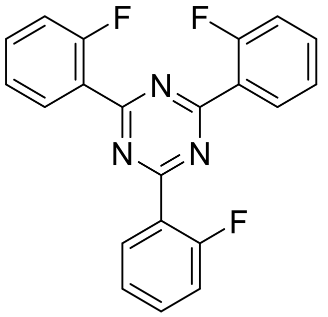

2,4,6-tris(2-fluorophenyl)-1,3,5-triazine(2g)

White solid; mp: 167.8-171.4 °C; **^{1}H NMR** (400 MHz, $CDCl_3$) δ 8.49-8.46 (t, J = 7.8 Hz, 3H), 7.61-7.56 (q, J = 6.8 Hz, 3H), 7.38-7.34 (t, J = 7.6 Hz, 3H), 7.31 – 7.28 (m, 3H). **^{13}C NMR** (100 MHz, $CDCl_3$) δ 170.80, 170.75, 163.70, 161.12, 133.74, 133.65, 132.38, 124.61, 124.53, 124.30, 124.26, 117.37, 117.15. **HRMS (EI)**: calcd for $C_{21}H_{12}N_3F_3$: 363.0983; found: 363.0976

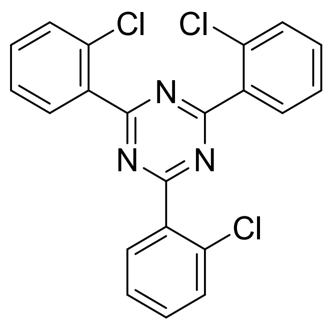

2,4,6-tris(2-chlorophenyl)-1,3,5-triazine(2h)

Yellow solid; mp: 201.5-203.7°C; Lit.mp: 202-203°C [10] **^{1}H NMR** (400 MHz, $CDCl_3$) δ 8.12-8.10 (m, 3H), 7.58-7.55 (m, 1H), 7.49-7.42 (m, Hz, 6H). **^{13}C NMR** (100 MHz, $CDCl_3$) δ 172.73, 135.35, 133.63, 132.66, 131.91, 131.13, 126.99. **HRMS (EI)**: calcd for $C_{21}H_{12}N_3Cl_3$: 411.0097; found: 411.0103

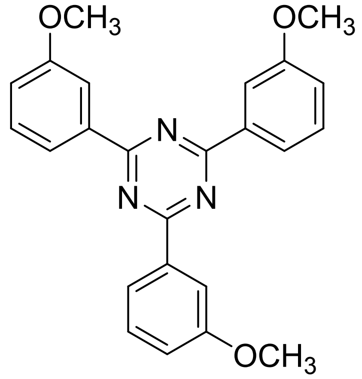

2,4,6-tris(3-methoxyphenyl)-1,3,5-triazine(2n)

White solid; mp: 172.3-176.4 °C; **^{1}H NMR** (400 MHz, $CDCl_3$) δ 8.37-8.35 (d, J = 8.0 Hz, 3H), 8.30-8.29 (m, 3H), 7.50-7.46 (t, J = 8.0 Hz, 3H), 7.17 – 7.14 (m, 3H), 3.96 (s, 9H). **^{13}C NMR** (100 MHz, $CDCl_3$) δ 171.42, 159.91, 137.61, 129.63, 121.52, 118.44, 113.88, 55.44. **HRMS (EI)**: calcd for $C_{24}H_{21}N_3O_3$: 399.1583; found: 399.1589

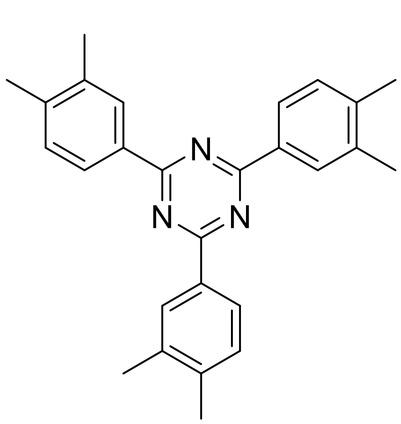

2,4,6-tris(3,4-dimethylphenyl)-1,3,5-triazine(2o)

White solid; mp: 212.8-214.4 °C; **^{1}H NMR** (400 MHz, $CDCl_3$) δ 8.51-8.49 (d, J = 9.2 Hz, 6H), 7.34-7.32 (d, J = 7.6 Hz, 3H), 2.44-2.39 (d, J = 20.8 Hz, 18H). **^{13}C NMR** (100 MHz, $CDCl_3$) δ 171.50, 141.50, 136.79, 134.17, 129.94, 129.87, 126.58, 20.06, 19.94.

HRMS (EI): calcd for $C_{27}H_{27}N_3$:393.2205; found: 393.2201

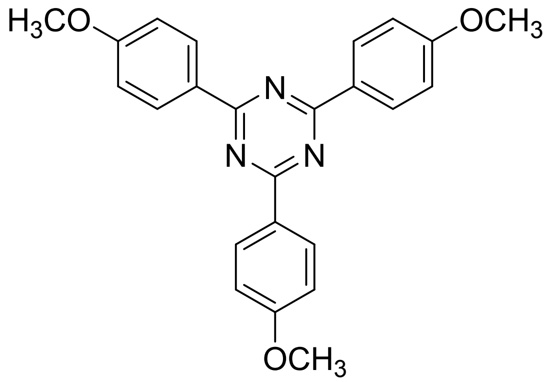

2,4,6-tris(4-methoxyphenyl)-1,3,5-triazine(2m)

White solid; mp: 220.4-222.6 °C; Lit.mp: 219-220°C [4] **¹H NMR** (400 MHz, $CDCl_3$) δ 8.71-8.69 (d, J = 9.2 Hz, 6H), 7.06-7.04 (d, J =9.6 Hz, 6H), 3.91 (s, 9H). **¹³C NMR** (100 MHz, $CDCl_3$) δ 170.68, 163.08, 130.68, 129.13, 113.82, 55.41. **HRMS (EI)**: calcd for $C_{24}H_{21}N_3O_3$: 399.1583; found: 399.1576

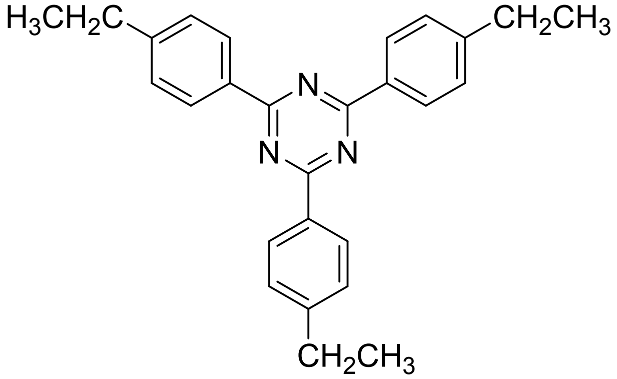

2,4,6-tris(4-ethylphenyl)-1,3,5-triazine(2i)

White solid; mp: 112.8-114.6 °C; Lit.mp: 114-115 °C [9] **¹H NMR** (400 MHz, $CDCl_3$) δ 8.70-8.68 (d, J = 7.6 Hz, 6H), 7.41-7.39 (d, J = 7.6 Hz, 6H), 2.81-2.76 (q, J = 7.4 Hz, 6H), 1.35-1.31 (t, J = 7.6 Hz, 9H). **¹³C NMR** (100 MHz, $CDCl_3$) δ 171.43, 149.09, 134.03, 129.01, 128.11, 29.02, 15.37. **HRMS (EI)**: calcd for $C_{27}H_{27}N_3$: 393.2205; found: 393.2213

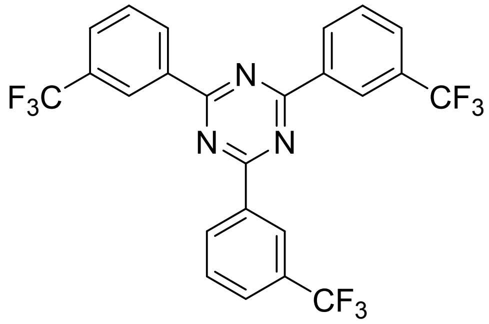

2,4,6-tris(3-(trifluoromethyl)phenyl)-1,3,5-triazine(2q)

White solid; mp: 210.1-211.6 °C; ^{1}**H NMR** (400 MHz, $CDCl_3$) δ 8.97 – 8.92 (m, 6H), 7.91-7.89 (d, J = 7.8 Hz, 3H), 7.76-7.72 (t, J = 7.8 Hz, 3H). ^{13}**C NMR** (100 MHz, $CDCl_3$) δ 170.98, 136.32, 132.21, 131.63, 131.30, 129.43, 125.82, 125.79, 125.29, 122.58. **HRMS (EI)**: calcd for $C_{24}H_{12}N_3F_9$: 513.0888; found: 513.0883

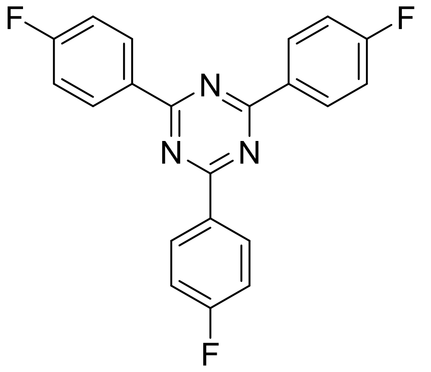

2,4,6-tris(4-fluorophenyl)-1,3,5-triazine(2p)

White solid; mp: 260.3-261.5 °C; Lit.mp: 261.2-262°C [5] ^{1}**H NMR** (400 MHz, $CDCl_3$) δ 8.81-8.78 (dd, J = 8.7, 5.6 Hz, 6H), 7.30-7.28 (m, 6H). ^{13}**C NMR** (100 MHz, $CDCl_3$) δ 170.71, 167.16, 132.21, 131.35, 131.26, 115.88, 115.67. **HRMS (EI)**: calcd for $C_{21}H_{12}N_3F_3$: 363.0983; found:363.0991

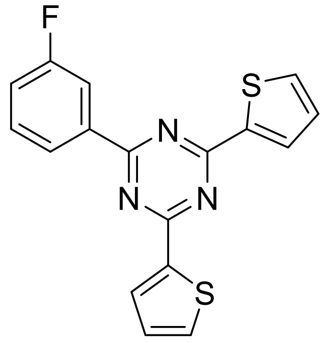

2-(3-fluorophenyl)-4,6-di(thiophen-2-yl)-1,3,5-triazine(3a)

White solid; mp: 218.3-219.7 °C; ^{1}**H NMR** (400 MHz,$CDCl_3$) δ 8.46-8.44 (d, J = 8.0 Hz, 1H), 8.34 – 8.31 (m, 3H), 7.65-7.64 (d, J = 5.2 Hz, 2H), 7.54 – 7.49 (m, 1H), 7.31-7.28 (m, 1H) 7.24-7.22 (t, J = 4.4 Hz, 2H). ^{13}**C NMR** (100 MHz, $CDCl_3$) δ 170.34, 170.31, 168.00, 164.28, 161.84, 141.51, 132.49, 131.80, 130.12, 130.04, 128.49, 124.57, 124.54, 119.55, 119.34, 115.74, 115.50. ^{19}**F NMR** (376 MHz, $CDCl_3$) δ -112.50 – -112.91 (m). **HRMS (EI)**: calcd for $C_{17}H_{10}N_3S_2F$: 339.0300; found: 339.0305

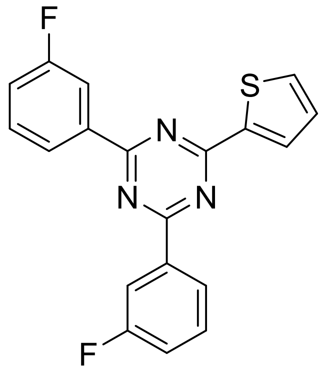

2,4-bis(3-fluorophenyl)-6-(thiophen-2-yl)-1,3,5-triazine(4a)

White solid; mp: 190.3-192.7 °C; **¹H NMR** (400 MHz, $CDCl_3$) δ 8.51-8.49 (d, J = 8.0 Hz, 2H), 8.39 – 8.36 (m, 3H), 7.68-7.66 (dd, J = 4.8, 1.2 Hz, 1H), 7.57-7.51 (m, 2H), 7.33-7.29 (td, J = 8.2, 2.4 Hz, 2H), 7.25-7.24 (m, 1H).**¹³C NMR** (100 MHz, $CDCl_3$) δ 170.62, 168.31, 164.33, 161.89, 141.56, 138.13, 138.05, 132.71, 131.95, 130.22, 130.14, 128.60, 124.63, 124.60, 119.71, 119.49, 115.80, 115.56. **¹⁹F NMR** (376 MHz, $CDCl_3$) δ -102.32 – -116.72 (m,2F). **HRMS (EI)**: calcd for $C_{19}H_{11}N_3SF_2$: 351.0642; found: 351.0646

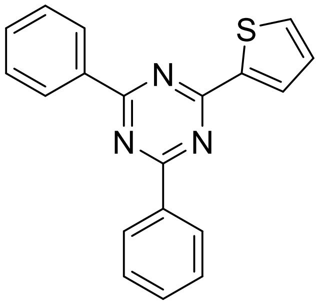

2,4-diphenyl-6-(thiophen-2-yl)-1,3,5-triazine(3b)

White solid; mp: 254.6-256.2 °C; Lit.mp: 255.2-257°C [6] **¹H NMR** (400 MHz, $CDCl_3$) δ 8.74-8.71 (dd, J = 8.0, 1.6 Hz, 4H), 8.38-8.37 (dd, J = 4.0, 1.4 Hz, 1H), 7.65 – 7.55 (m, 7H), 7.25 – 7.24 (m, 1H). **¹³C NMR** (100 MHz, $CDCl_3$) δ 171.55, 168.09, 142.15, 135.93, 132.55, 132.15, 131.49, 128.94, 128.60, 128.46. **HRMS (EI)**: calcd for $C_{19}H_{13}N_3S$: 315.0830; found: 315.0837

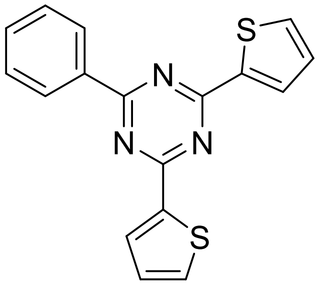

2-phenyl-4,6-di(thiophen-2-yl)-1,3,5-triazine(4b)

White solid; mp: 181.5-183.7 °C; **¹H NMR** (400 MHz, $CDCl_3$) δ 8.67-8.66 (d, J = 6.8 Hz, 2H), 8.33-8.32 (d, J = 3.6 Hz, 2H), 7.64-7.63 (d, J = 4.8 Hz, 2H), 7.60-7.54 (m, 3H), 7.24 – 7.22 (t, J=4.0, 2H). **¹³C NMR** (100 MHz, $CDCl_3$) δ 171.41, 167.91, 141.80,

135.60, 132.59, 132.23, 131.59, 128.91, 128.57, 128.44. **HRMS (EI)**: calcd for $C_{17}H_{11}N_3S_2$: 321.0394; found: 321.0399

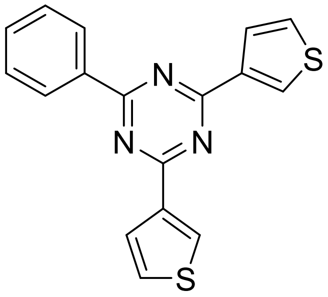

2-phenyl-4,6-di(thiophen-3-yl)-1,3,5-triazine(4c)

White solid; mp: 196.2-198.8 °C; **^{1}H NMR** (400 MHz, $CDCl_3$) δ 8.70 – 8.68 (m, 2H), 8.65-8.64 (dd, J = 3.0, 1.0 Hz, 2H), 8.11-8.10 (d, J = 5.2 Hz, 2H), 7.62-7.54 (m, 3H), 7.45-7.43 (dd, J = 5.0, 2.4 Hz, 2H). **^{13}C NMR** (100 MHz, $CDCl_3$) δ 171.68, 168.34, 140.53, 136.07, 132.43, 131.02, 128.82, 128.56, 127.67, 126.22. **HRMS (EI)**: calcd for $C_{17}H_{11}N_3S_2$: 321.0394; found: 321.0390

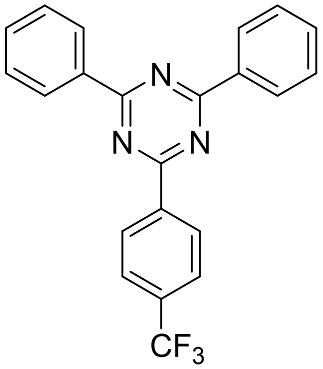

2,4-diphenyl-6-(4-(trifluoromethyl)phenyl)-1,3,5-triazine(3d)

White solid; mp: 183.3.2-185.2 °C; **^{1}H NMR** (400 MHz, $CDCl_3$) δ 8.89-8.87 (d, J = 8.2 Hz, 2H), 8.79-8.76 (m, 4H), 7.84-8.72 (d, J = 8.0 Hz, 2H), 7.66 – 7.57 (m, 6H). **^{13}C NMR** (100 MHz, $CDCl_3$) δ 171.94, 170.47, 139.59, 135.89, 132.79, 129.23, 129.02, 128.72, 125.57, 125.53. **HRMS (EI)**: calcd for $C_{22}H_{14}N_3F_3$: 377.1140; found: 377.1133

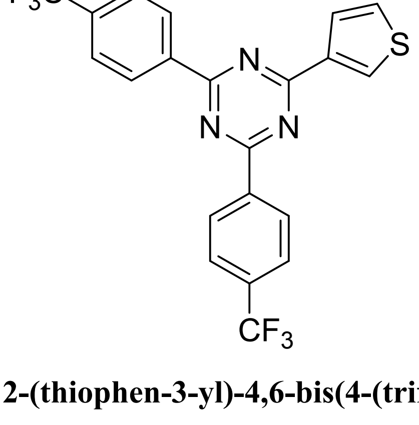

2-(thiophen-3-yl)-4,6-bis(4-(trifluoromethyl)phenyl)-1,3,5-triazine(4e)

White solid; mp: 149.1-152.3 °C; **^{1}H NMR** (400 MHz, $CDCl_3$) δ 8.78-8.76 (d, J = 8.0 Hz, 4H), 8.67 (d, J = 2.0 Hz, 1H), 8.09-8.08 (d, J =4.8 Hz, 1H), 7.81-7.79 (d, J = 8.0 Hz, 4H), 7.47-7.45 (dd, J = 5.0, 2.4 Hz, 1H). **^{13}C NMR** (100 MHz, $CDCl_3$) δ 170.66, 168.65, 139.85, 139.04, 134.23, 133.91, 131.93, 129.16, 127.57, 126.63, 125.63, 125.59, 125.55, 125.51, 125.25, 122.54. **^{19}F NMR** (376 MHz, $CDCl_3$) δ -62.92. **HRMS (EI)**: calcd for $C_{21}H_{11}N_3F_6S$: 451.0578; found: 451.0572

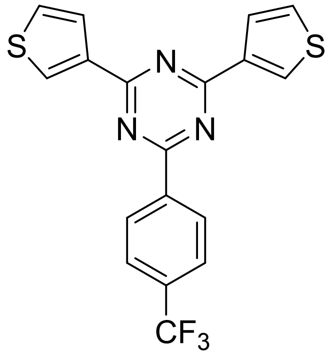

2,4-di(thiophen-3-yl)-6-(4-(trifluoromethyl)phenyl)-1,3,5-triazine(3e)

White solid; mp: 175.9-177.2 °C; **^{1}H NMR** (400 MHz, $CDCl_3$) δ 8.77-8.75 (d, J = 8.4 Hz, 2H), 8.64-8.63 (d, J = 2.4 Hz, 2H), 8.08 – 8.06 (m, 2H), 7.80-7.78 (d, J = 7.2 Hz, 2H), 7.45-7.43 (m, 2H). **^{13}C NMR** (100 MHz, $CDCl_3$) δ 170.42, 168.48, 140.16, 139.38, 133.92, 133.59, 131.40, 129.06, 127.59, 126.38, 125.50, 125.47, 125.43, 125.40. **^{19}F NMR** (376 MHz, $CDCl_3$) δ -62.85. **HRMS (EI)**: calcd for $C_{18}H_{10}N_3F_3S_2$: 389.0268; found: 389.0274

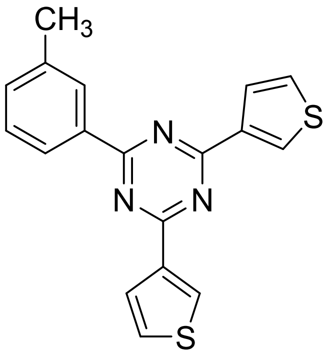

2,4-di(thiophen-2-yl)-6-(m-tolyl)-1,3,5-triazine(3f)

White solid; mp: 142.7-145.6 °C; **^{1}H NMR** (400 MHz, $CDCl_3$) δ 8.47-7.46 (d, J = 7.2 Hz, 2H), 8.33-8.32 (d, J = 3.6 Hz, 2H), 7.64-7.63 (d, J = 4.8 Hz, 2H), 7.46 – 7.40 (m, 2H), 7.24 – 7.22 (t, J = 4.4 Hz, 2H), 2.50 (s, 3H). **^{13}C NMR** (100 MHz, $CDCl_3$) δ 171.55, 167.86, 141.84, 138.24, 135.53, 133.43, 132.16, 131.55, 129.36, 128.49, 128.41, 126.16, 21.52. **HRMS (EI)**: calcd for $C_{18}H_{13}N_3S_2$: 335.0551; found: 335.0558

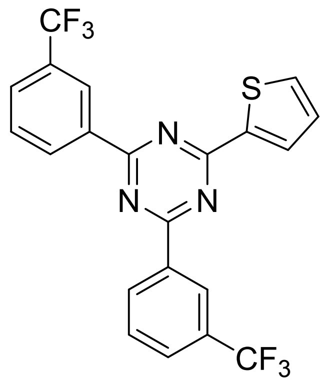

2-(thiophen-2-yl)-4,6-bis(3-(trifluoromethyl)phenyl)-1,3,5-triazine(4g)

White solid; mp: 145.2-147.3 °C; **^{1}H NMR** (400 MHz,CDCl$_3$) δ8.97– 8.91 (m, 4H), 8.42-8.41 (d, J = 4.0 Hz, 1H), 7.92-7.90 (d, J = 7.6 Hz, 2H), 7.77-7.73 (dd, J = 9.4, 6.4 Hz, 3H), 7.31 – 7.29 (t, J = 4.4 Hz, 1H). **^{13}C NMR** (100 MHz, CDCl$_3$) δ 170.51, 168.45, 141.26, 136.42, 133.07, 132.28, 132.10, 131.45, 131.12, 129.25, 129.20, 129.16, 128.71, 125.75, 125.71. **HRMS (EI)**: calcd for $C_{21}H_{11}N_3F_6S$: 451.0578; found: 451.0570

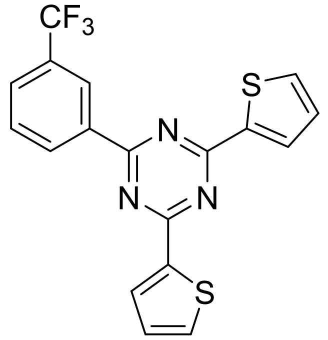

2,4-di(thiophen-2-yl)-6-(3-(trifluoromethyl)phenyl)-1,3,5-triazine(3g)

White solid; mp: 170.1-171.9 °C; **^{1}H NMR** (400 MHz, CDCl$_3$) δ 8.89 (s, 1H), 8.84-8.82 (d, J = 7.6 Hz, 1H), 8.33-8.31 (dd, J = 3.6, 0.8 Hz, 2H), 7.85-7.83 (d, J = 8.0 Hz, 1H), 7.69 – 7.64 (m, 3H), 7.24 – 7.22 (m, 2H). **^{13}C NMR** (100 MHz, CDCl$_3$) δ 170.07, 168.07, 141.36, 136.48, 132.64, 132.01, 131.95, 131.29, 130.96, 129.10, 128.95, 128.91, 128.54, 125.68, 125.64. **HRMS (EI)**: calcd for $C_{18}H_{10}N_3F_3S_2$: 389.0268; found: 389.0271

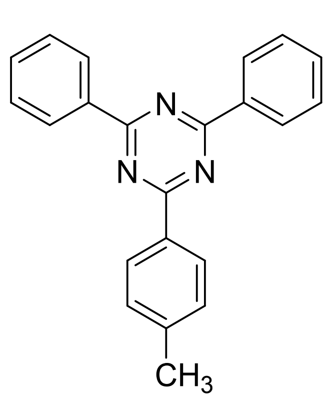

2,4-diphenyl-6-(p-tolyl)-1,3,5-triazine(3h)

White solid; mp: 196.1-198.5 °C; Lit.mp: 197-199°C [7] **^{1}H NMR** (400 MHz, $CDCl_3$) δ 8.79-8.77 (d, J = 7.6 Hz, 4H), 8.67-8.68 (d, J = 8.0 Hz, 2H), 7.62 – 7.56 (m, 6H), 7.39-7.37 (d, J =8.0 Hz, 2H), 2.49 (s, 1H). **^{13}C NMR** (100 MHz, $CDCl_3$) δ 171.66, 171.56, 143.12, 136.37, 133.56, 132.40, 129.40, 128.98, 128.94, 128.60, 21.73. **HRMS (EI)**: calcd for $C_{22}H_{17}N_3$: 323.1422; found: 323.1426

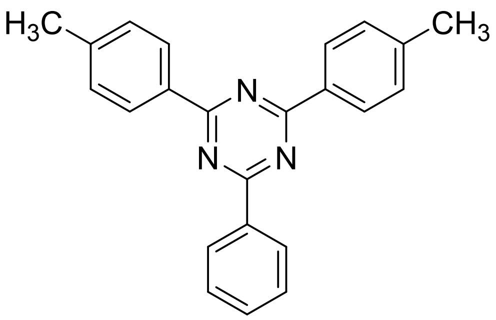

2-phenyl-4,6-di-p-tolyl-1,3,5-triazine(4h)

White solid; mp: 235.7-237.5 °C; Lit.mp: 236-238°C [8] **^{1}H NMR** (400 MHz, $CDCl_3$) δ 8.78-8.76 (d, J = 7.6 Hz, 2H), 8.68-8.66 (d, J = 8.0 Hz, 4H), 7.63-7.56 (m, 3H), 7.38-7.36 (d, J = 8.0 Hz, 4H), 2.48 (s, 6H). **^{13}C NMR** (100 MHz, $CDCl_3$) δ 171.53, 171.43, 142.98, 136.48, 133.66, 132.29, 129.36, 128.94, 128.91, 128.59, 128.56, 21.72. **HRMS (EI)**: calcd for $C_{23}H_{19}N_3$: 335.1579; found: 335.1584

5. NMR data of products

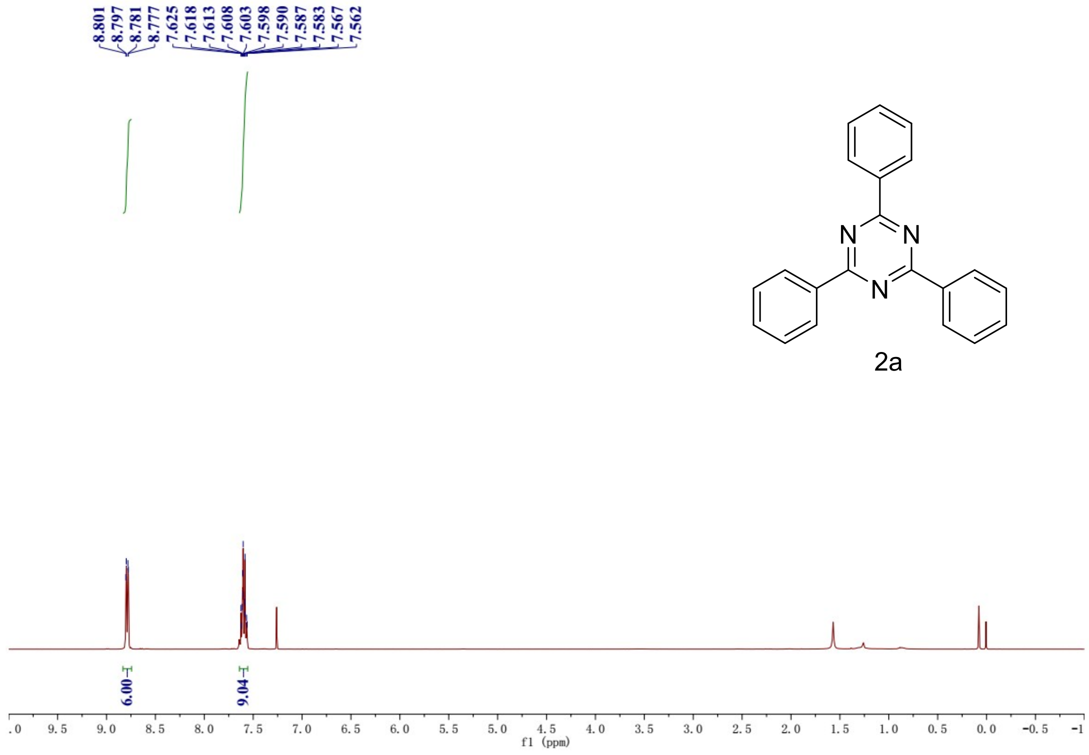

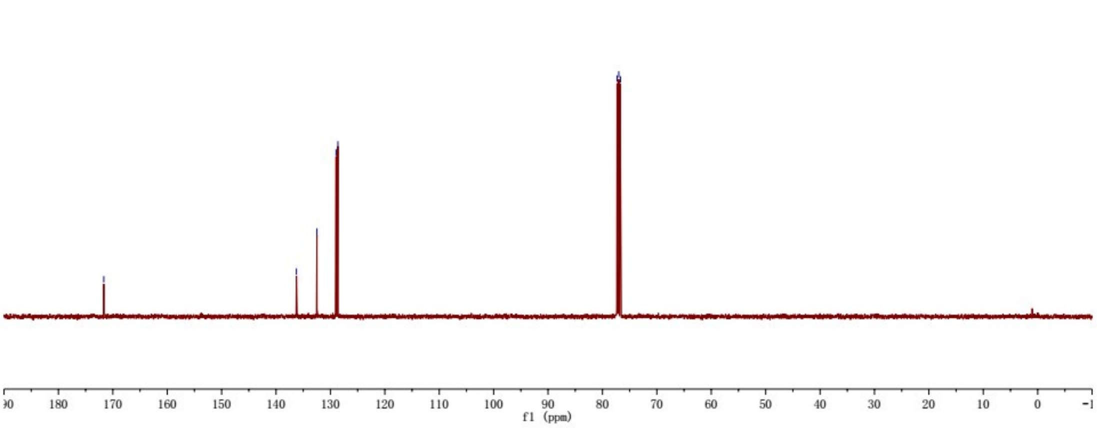

171.66
136.26
132.49
128.96
128.63
77.32
77.00
76.68
f1 (ppm)

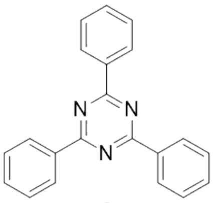

N
N
N

2a

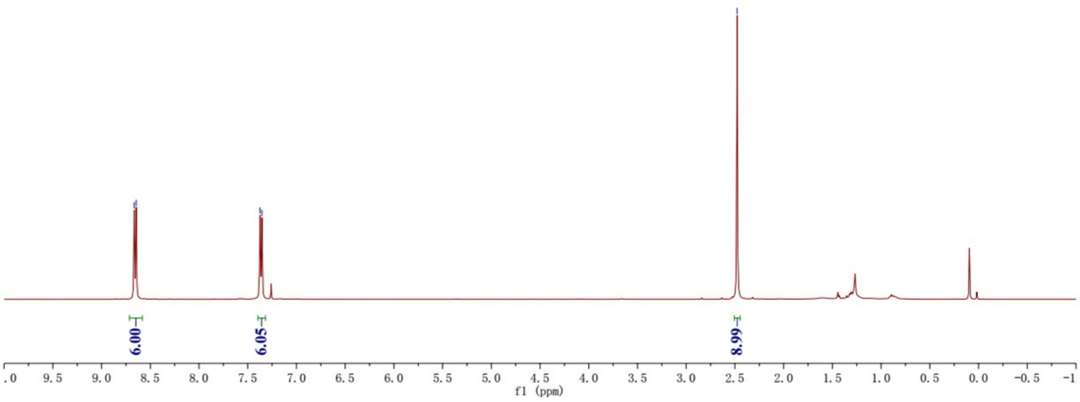

8.66
8.64
7.37
7.35
2.48
6.00
6.05
8.99
f1 (ppm)

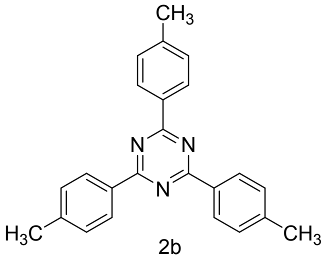

CH3
H3C
CH3
N
N
N

2b

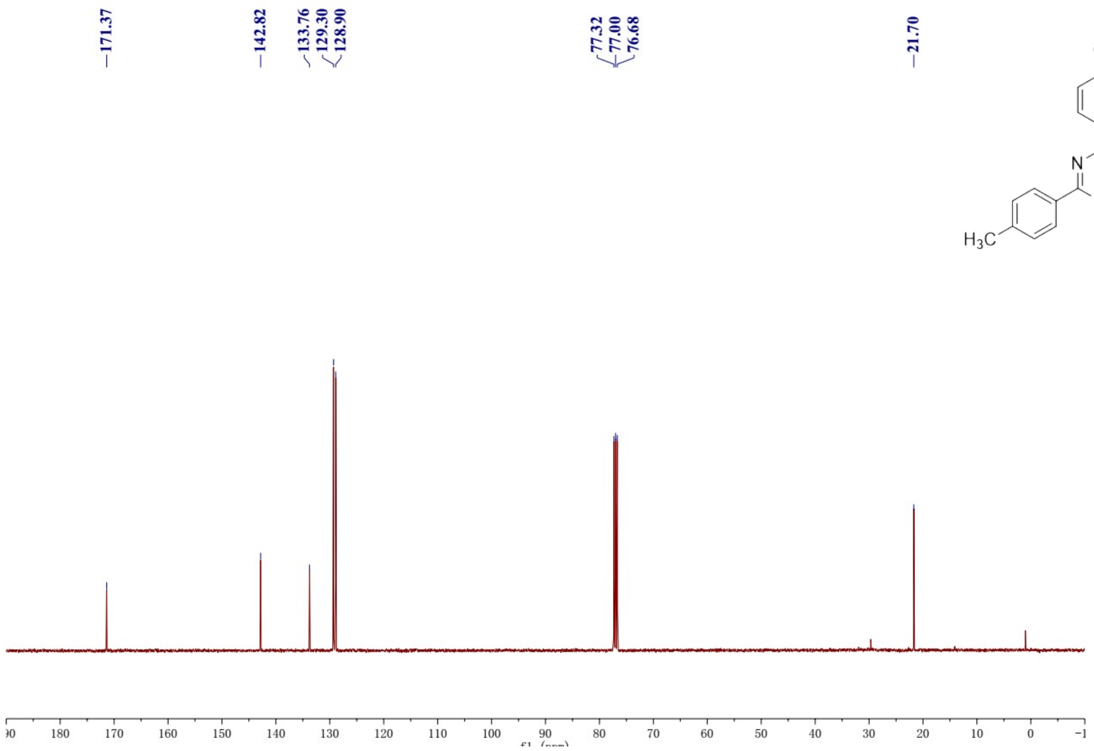
171.37
142.82
133.76
129.30
128.90
77.32
77.00
76.68
21.70
180 170 160 150 140 130 120 110 100 90 80 70 60 50 40 30 20 10 0

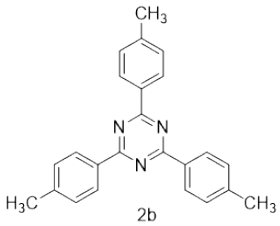
CH3
N
N
N
H3C
CH3
2b

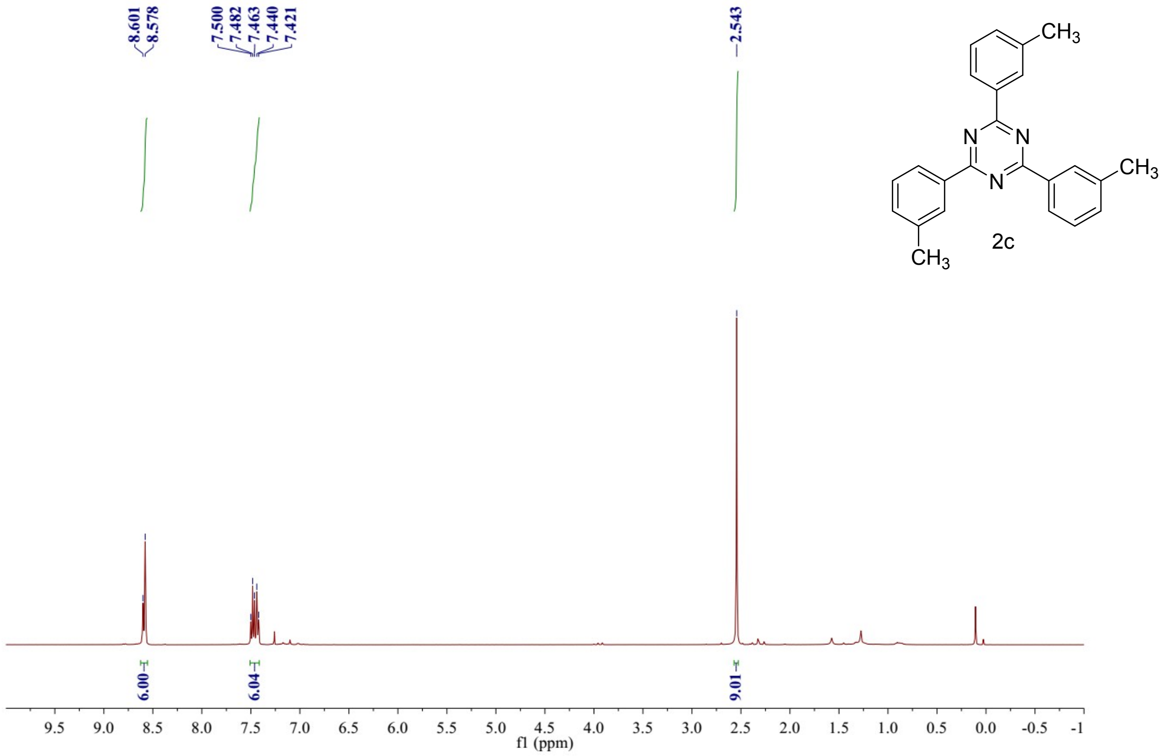
8.601
8.578
7.500
7.482
7.463
7.440
7.421
2.543
6.00
6.04
9.01
9.5 9.0 8.5 8.0 7.5 7.0 6.5 6.0 5.5 5.0 4.5 4.0 3.5 3.0 2.5 2.0 1.5 1.0 0.5 0.0 -0.5 -1
f1 (ppm)
CH3
N
N
N
CH3
CH3
2c

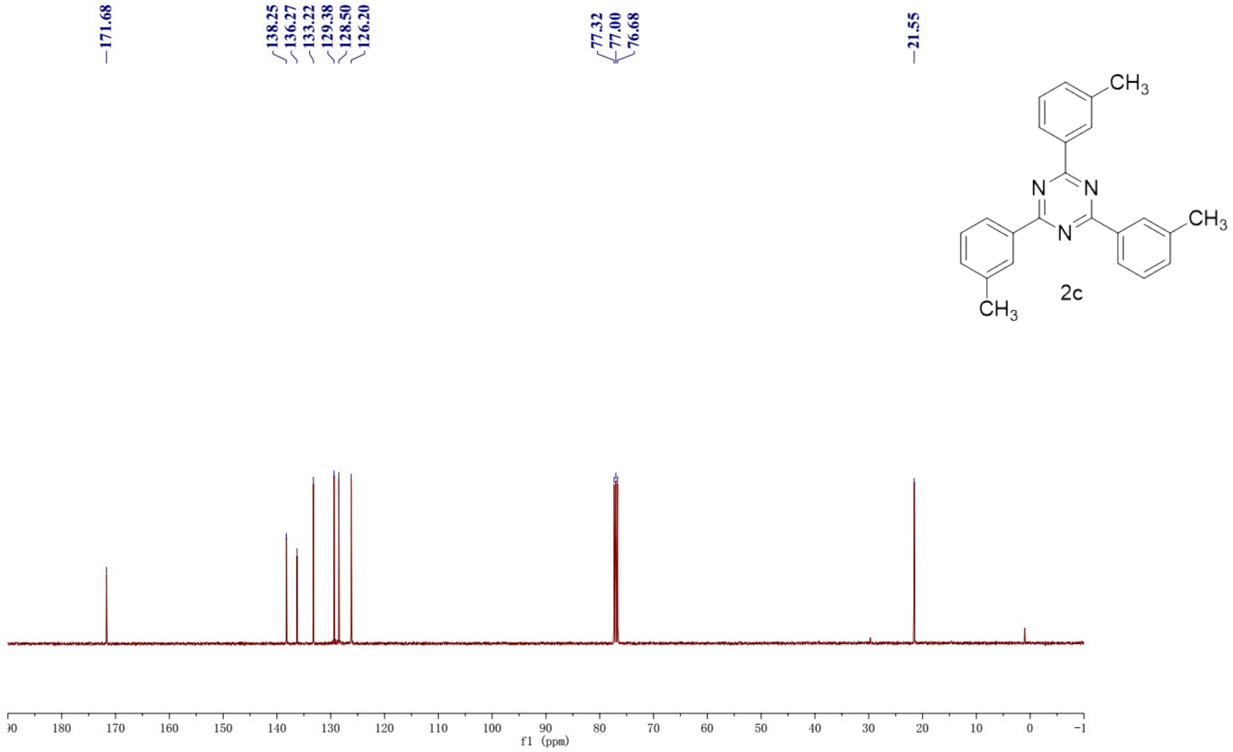
171.68
138.25
136.27
133.22
129.38
128.50
126.20
77.32
77.00
76.68
21.55
CH3
N
N
N
CH3
CH3
2c
180 170 160 150 140 130 120 110 100 90 80 70 60 50 40 30 20 10 0
f1 (ppm)

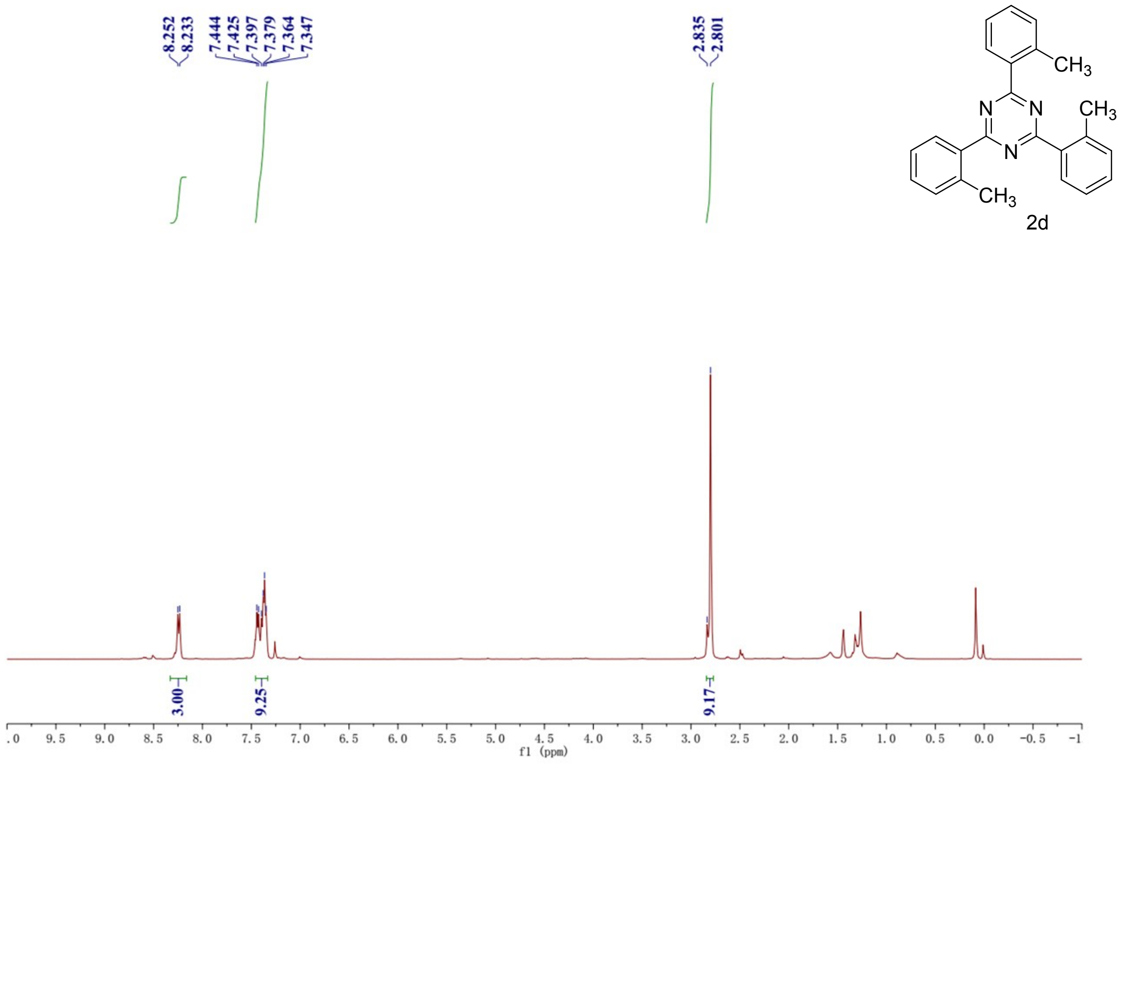
8.252
8.233
7.444
7.425
7.397
7.379
7.364
7.347
2.835
2.801
CH3
N
N
N
CH3
CH3
2d
3.00
9.25
9.17
9.5 9.0 8.5 8.0 7.5 7.0 6.5 6.0 5.5 5.0 4.5 4.0 3.5 3.0 2.5 2.0 1.5 1.0 0.5 0.0 -0.5
f1 (ppm)

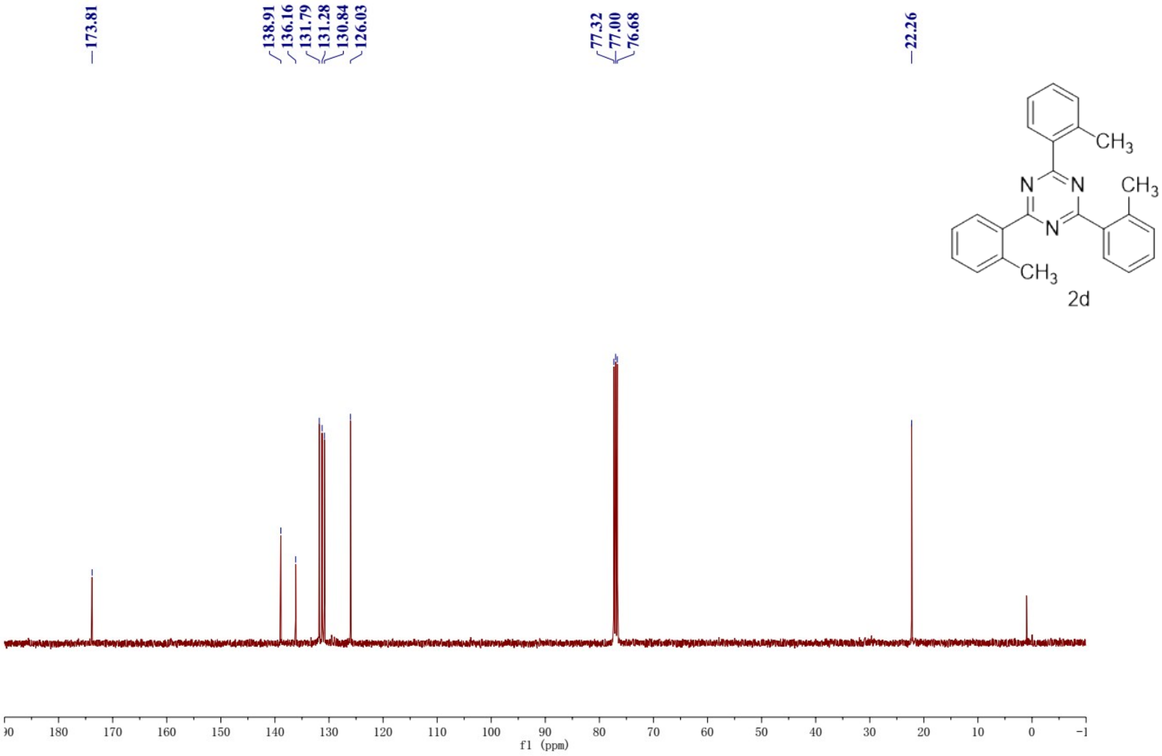

173.81
138.91
136.16
131.79
131.28
130.84
126.03
77.32
77.00
76.68
22.26
N
N
N
CH3
CH3
CH3
f1 (ppm)

2d

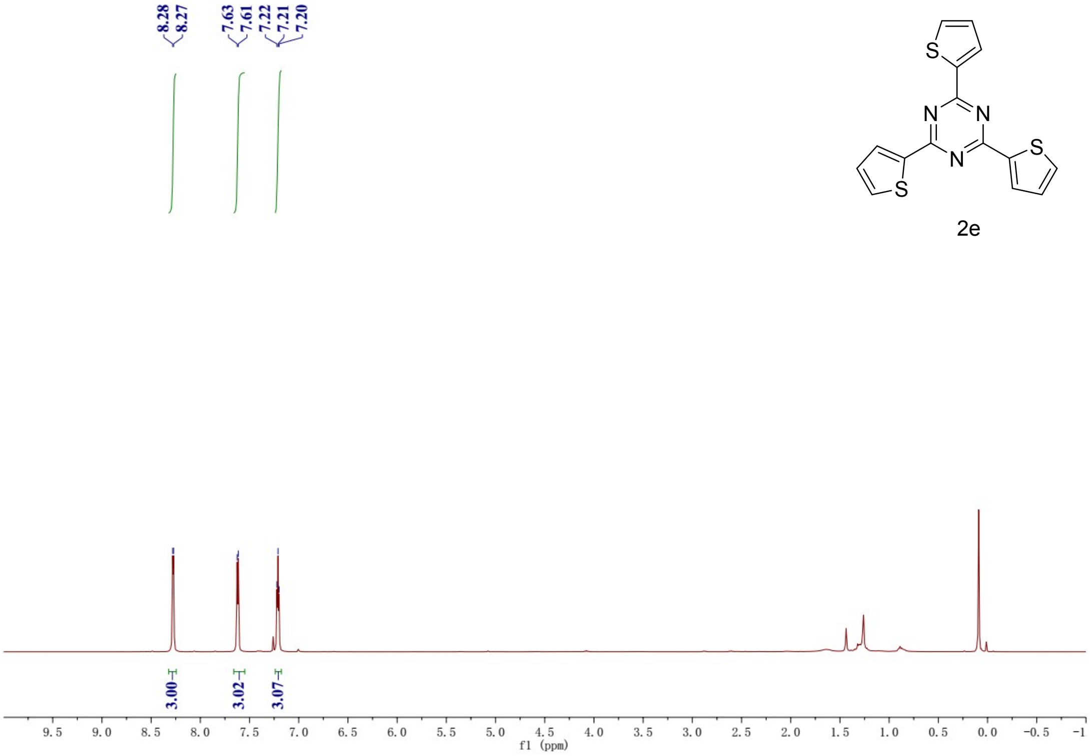

8.28
8.27
7.63
7.61
7.22
7.21
7.20
S
N
N
N
S
S
3.00
3.02
3.07
f1 (ppm)

2e

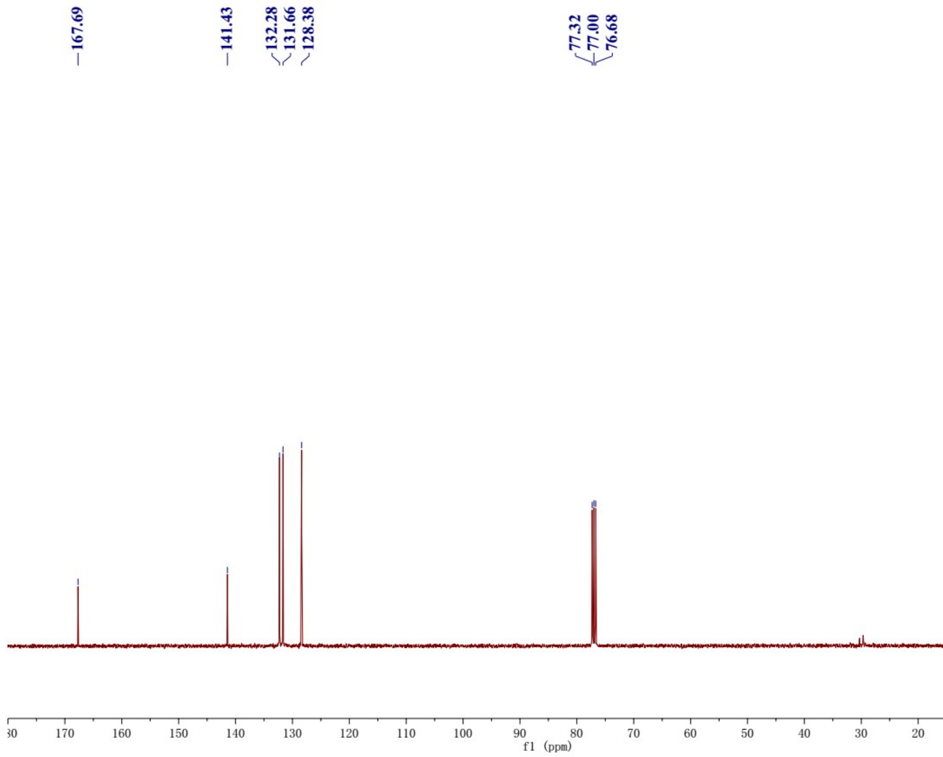

167.69
141.43
132.28
131.66
128.38
77.32
77.00
76.68
f1 (ppm)

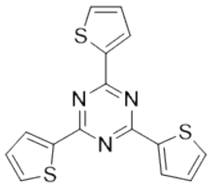

S
N
N
N
S
S

2e

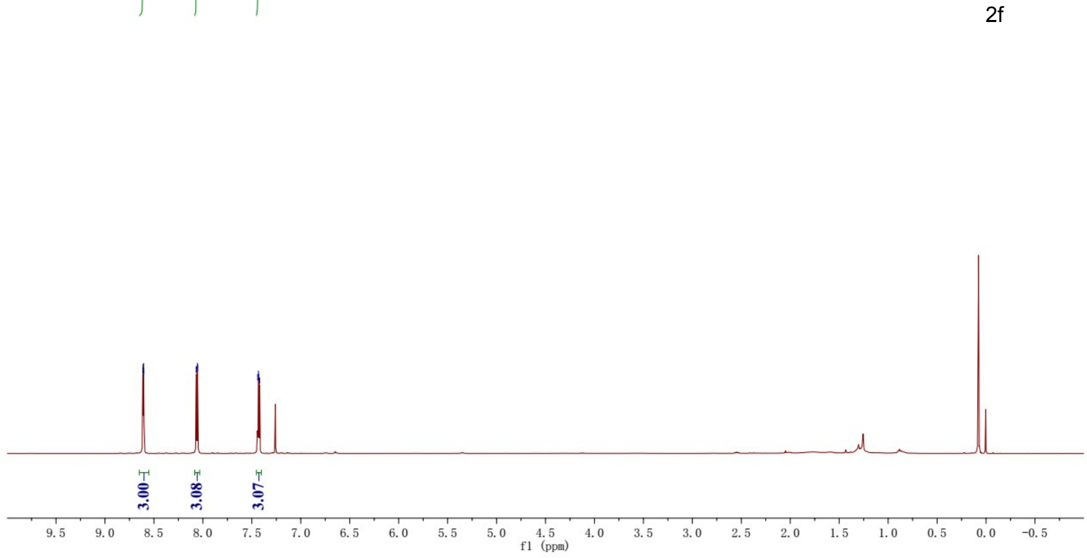

8.61
8.61
8.61
8.60
8.07
8.07
8.06
8.05
7.44
7.43
7.43
7.42
3.00
3.08
3.07
f1 (ppm)

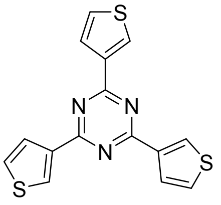

S
N
N
N
S
S

2f

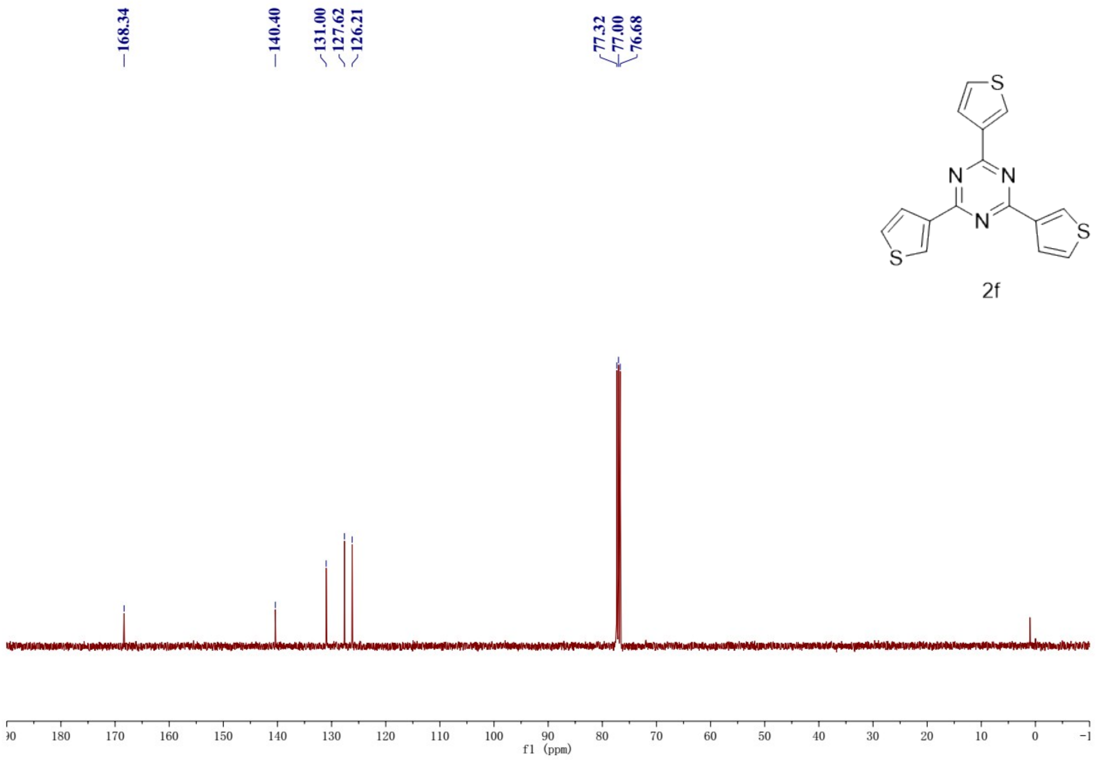
168.34
140.40
131.00
127.62
126.21
77.32
77.00
76.68
S
N
N
N
S
S
2f
90
180
170
160
150
140
130
120
110
100
90
80
70
60
50
40
30
20
10
0
-1
f1 (ppm)

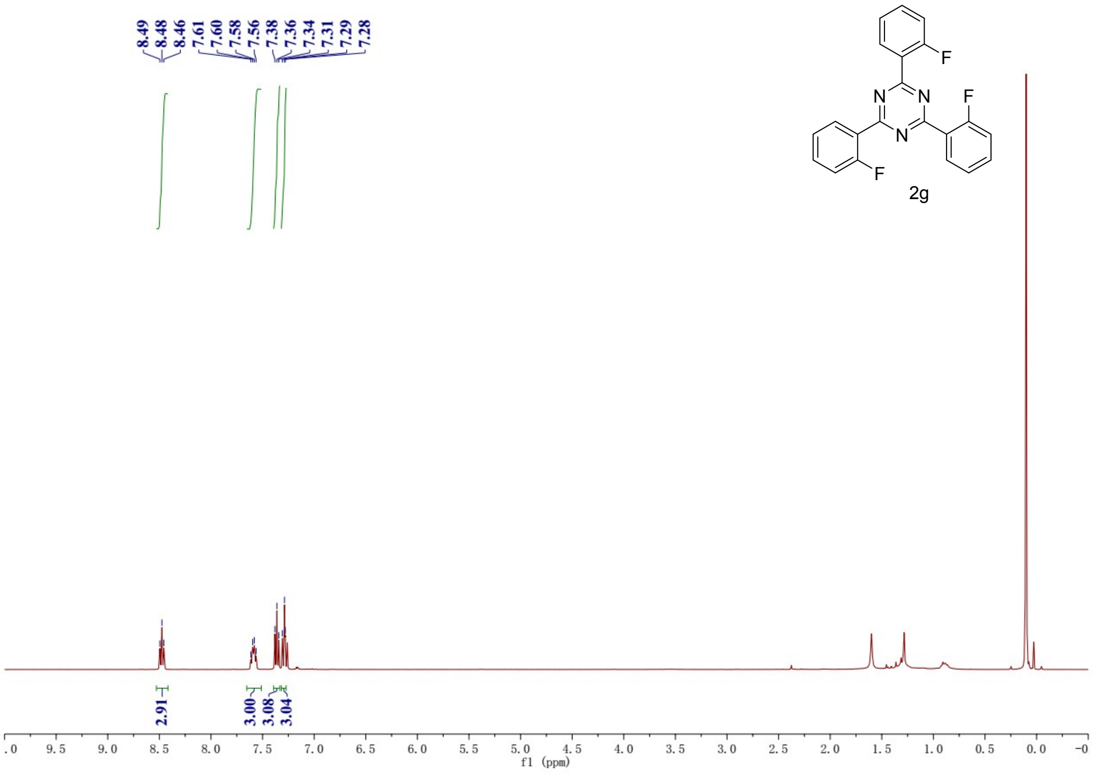
8.49
8.48
8.46
7.61
7.60
7.58
7.56
7.38
7.36
7.34
7.31
7.29
7.28
F
N
N
F
N
F
2g
2.91
3.00
3.08
3.04
.0
9.5
9.0
8.5
8.0
7.5
7.0
6.5
6.0
5.5
5.0
4.5
4.0
3.5
3.0
2.5
2.0
1.5
1.0
0.5
0.0
-0
f1 (ppm)

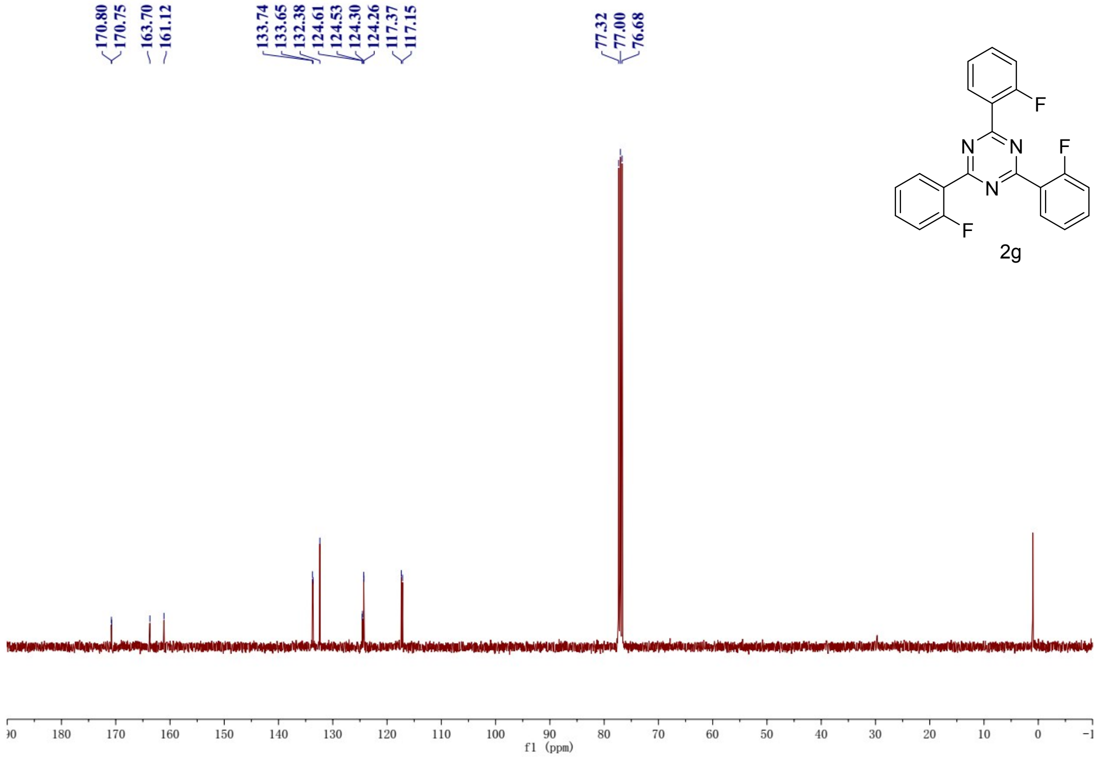
170.80
170.75
163.70
161.12
133.74
133.65
132.38
124.61
124.53
124.30
124.26
117.37
117.15
77.32
77.00
76.68
F
N
N
N
F
F
2g
180 170 160 150 140 130 120 110 100 90 80 70 60 50 40 30 20 10 0
f1 (ppm)

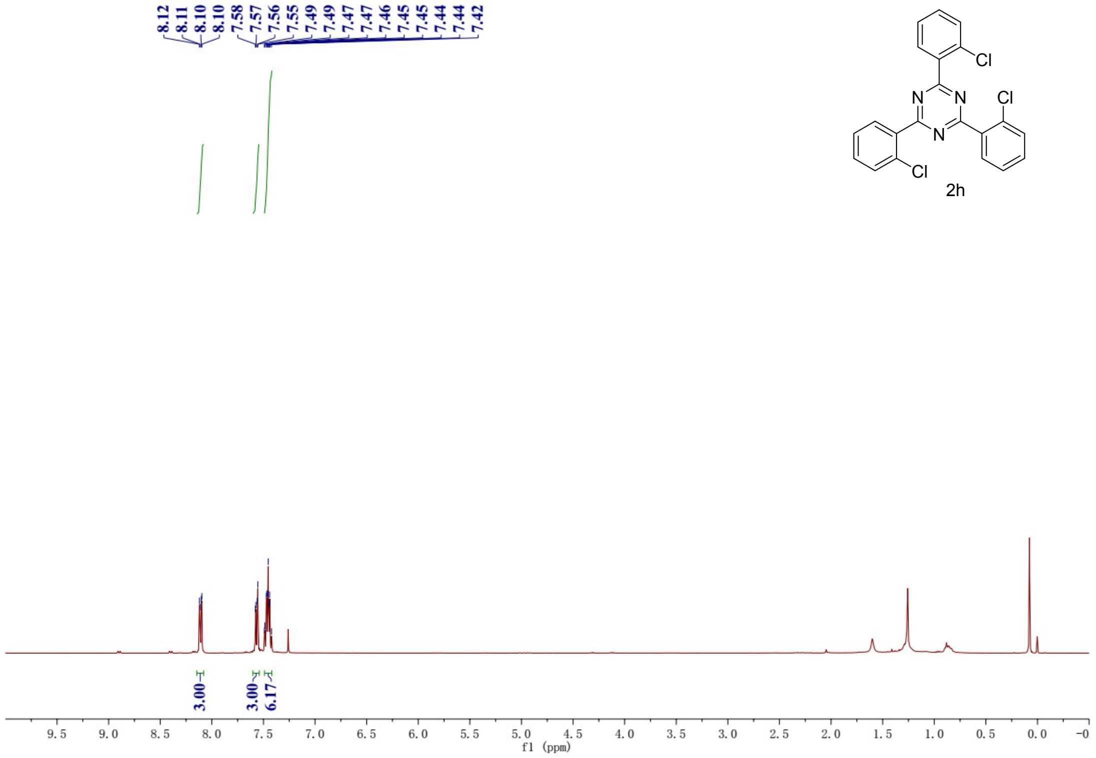
8.12
8.11
8.10
8.10
7.58
7.57
7.56
7.55
7.49
7.49
7.47
7.46
7.45
7.45
7.44
7.44
7.42
Cl
N
N
N
Cl
Cl
2h
3.00
3.00
6.17
9.5 9.0 8.5 8.0 7.5 7.0 6.5 6.0 5.5 5.0 4.5 4.0 3.5 3.0 2.5 2.0 1.5 1.0 0.5 0.0
f1 (ppm)

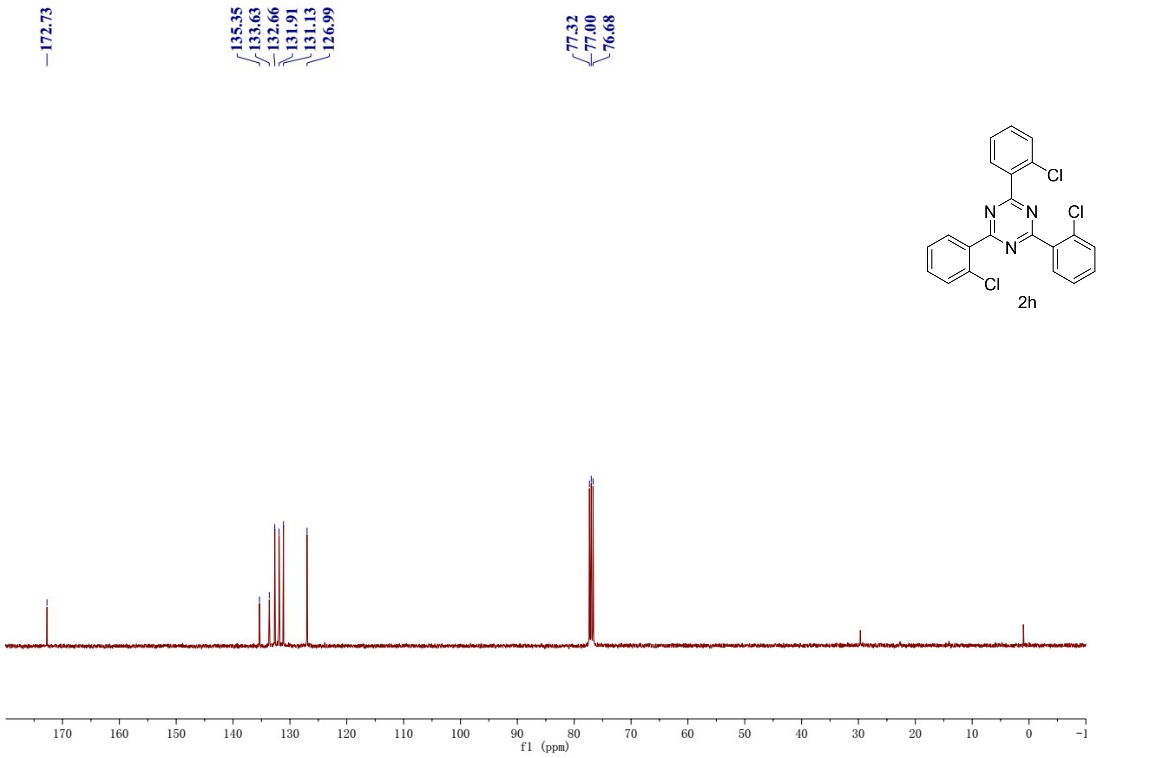
172.73
135.35
133.63
132.66
131.91
131.13
126.99
77.32
77.00
76.68
Cl
N
N
N
Cl
Cl
2h
f1 (ppm)

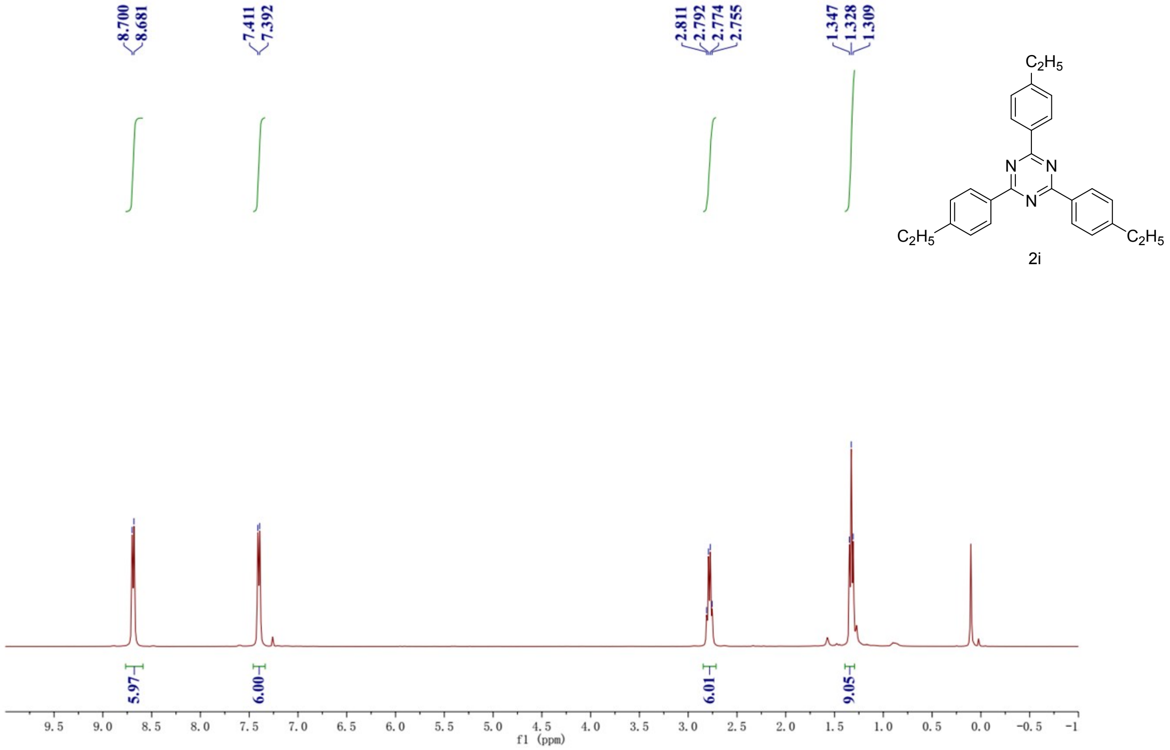
8.700
8.681
7.411
7.392
2.811
2.792
2.774
2.755
1.347
1.328
1.309
C_2H_5
N
N
N
C_2H_5
C_2H_5
2i
5.97
6.00
6.01
9.05
f1 (ppm)

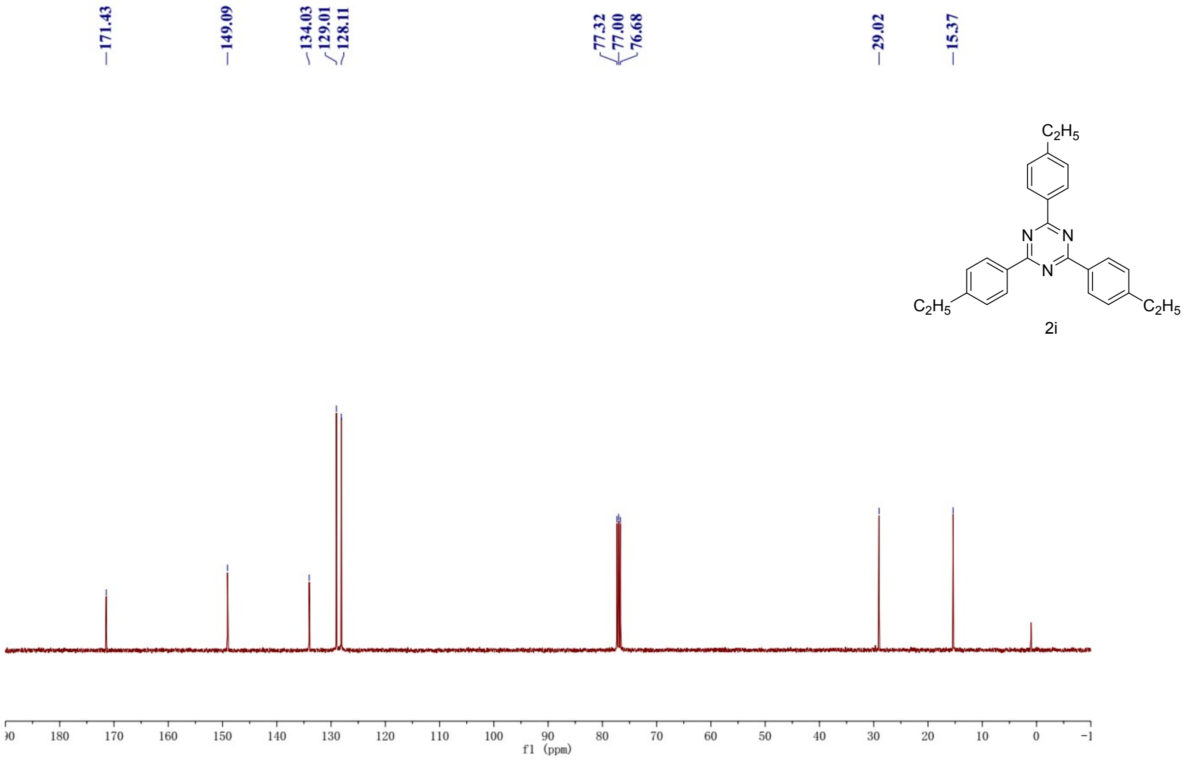
171.43
149.09
134.03
129.01
128.11
77.32
77.00
76.68
29.02
15.37
C2H5
N
N
N
C2H5
C2H5
2i
f1 (ppm)

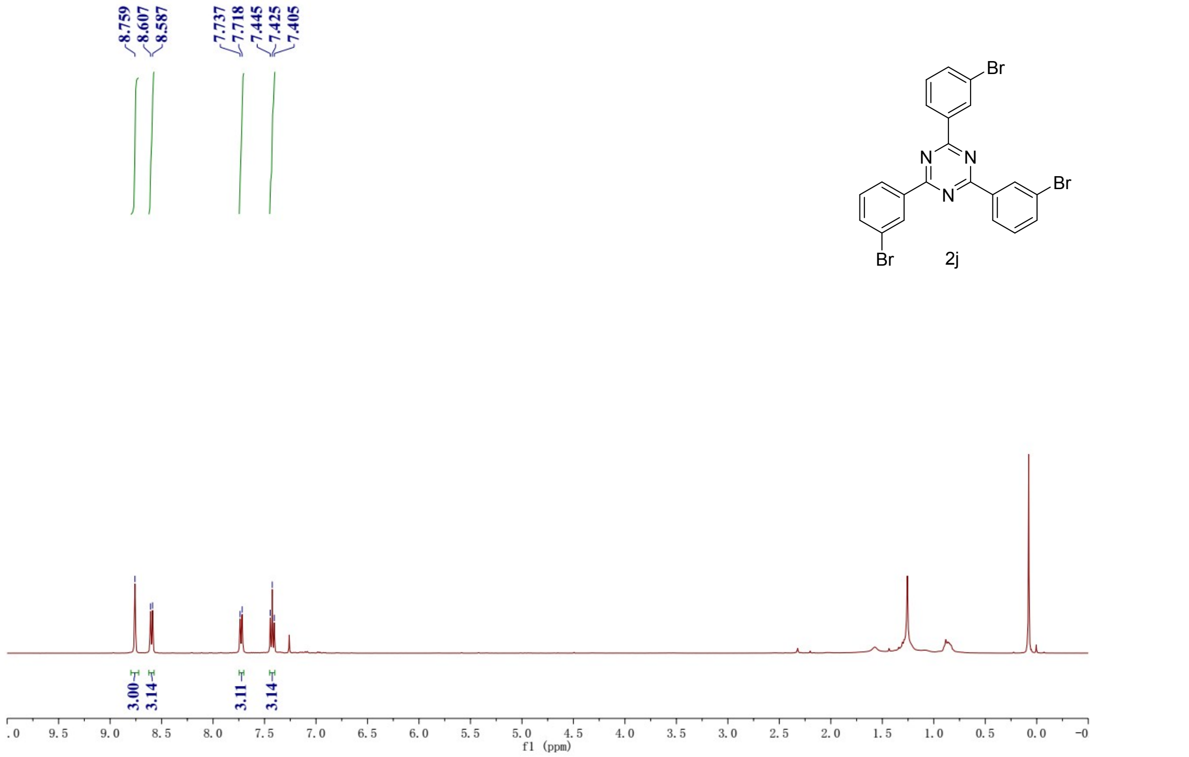
8.759
8.607
8.587
7.737
7.718
7.445
7.425
7.405
Br
N
N
N
Br
Br
2j
3.00
3.14
3.11
3.14
f1 (ppm)

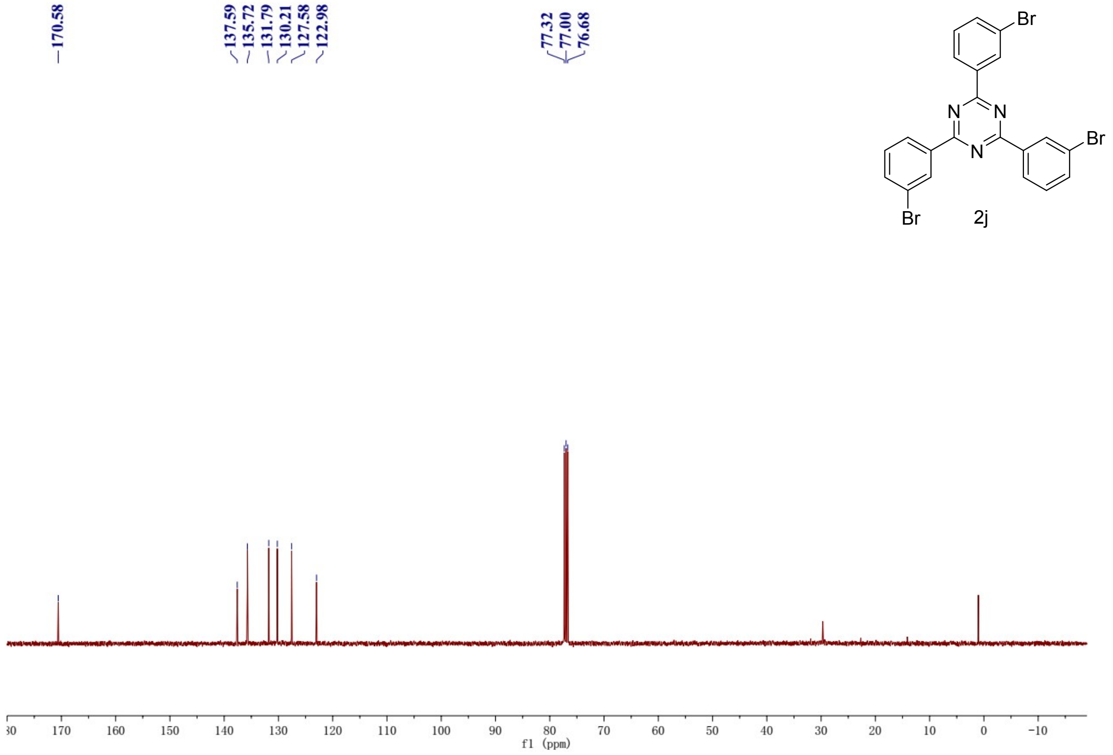
170.58
137.59
135.72
131.79
130.21
127.58
122.98
77.32
77.00
76.68
Br
N
N
N
Br
Br
f1 (ppm)
2j

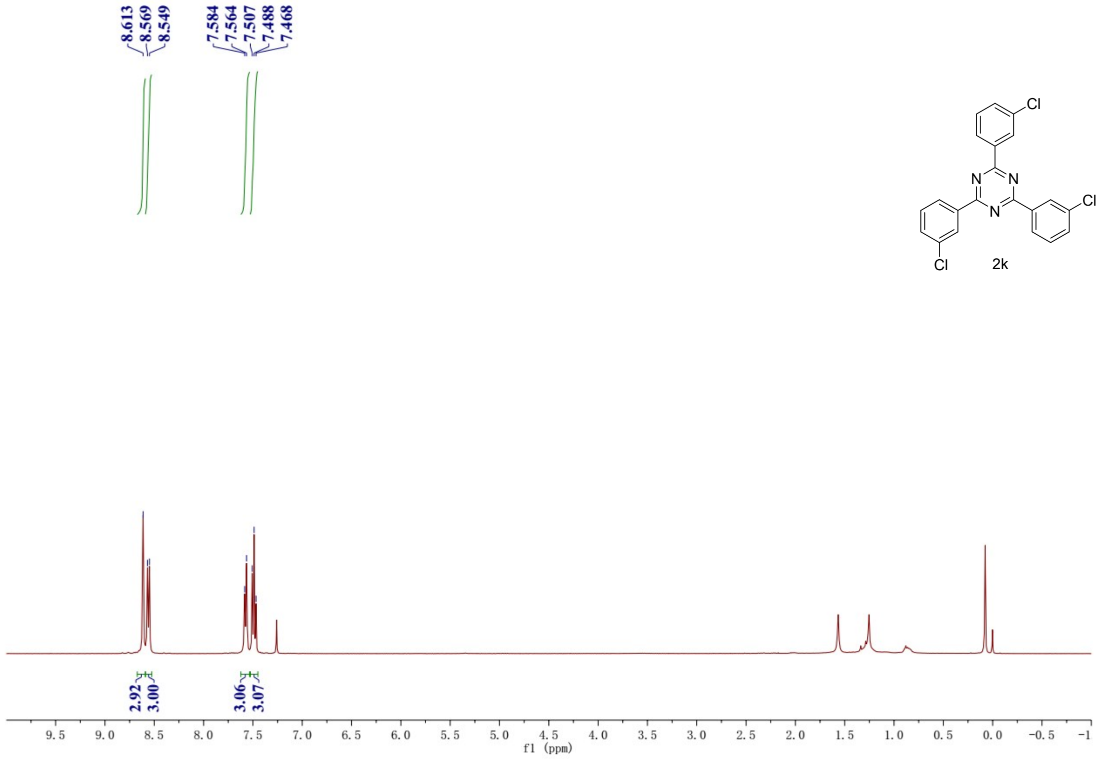
8.613
8.569
8.549
7.584
7.564
7.507
7.488
7.468
Cl
N
N
N
Cl
Cl
2.92
3.00
3.06
3.07
f1 (ppm)
2k

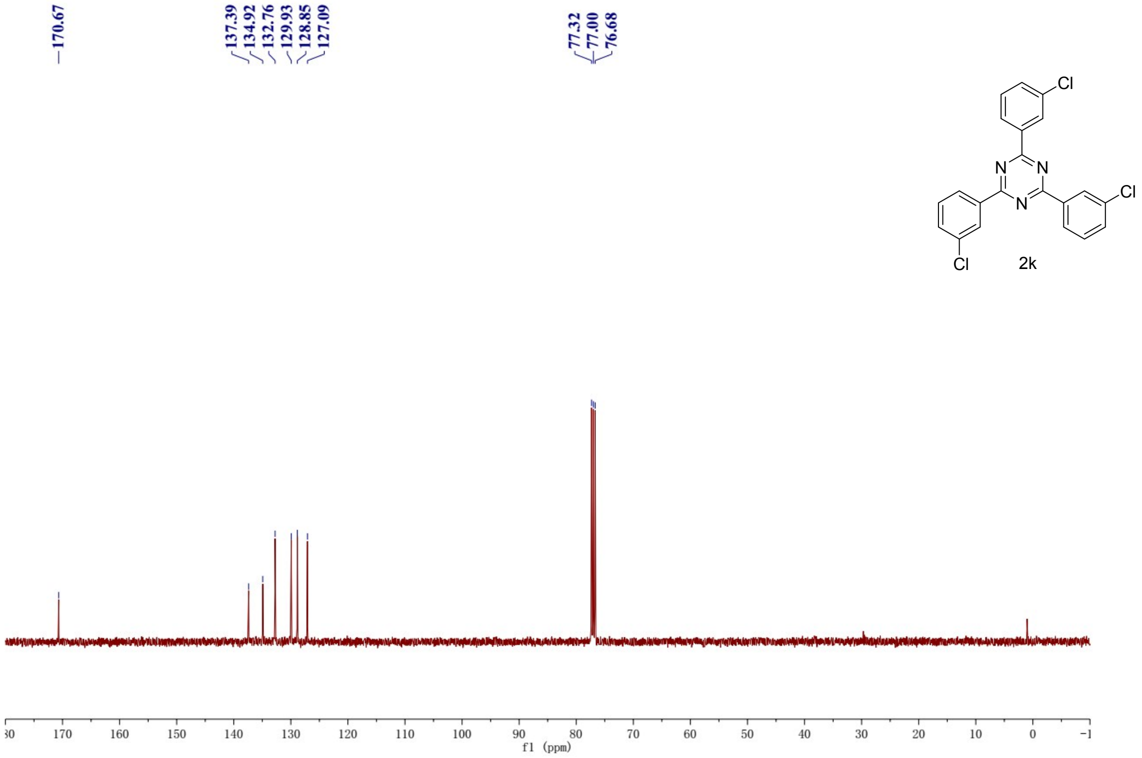
170.67
137.39
134.92
132.76
129.93
128.85
127.09
77.32
77.00
76.68
Cl
N
N
N
Cl
Cl
2k
f1 (ppm)

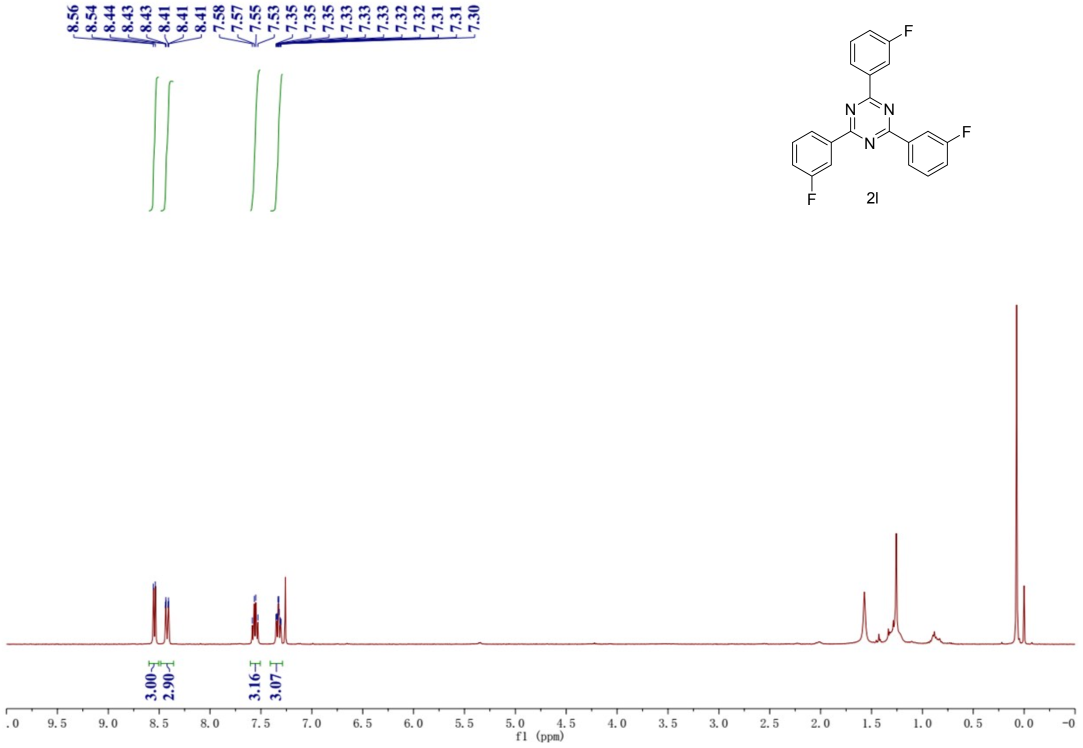
8.56
8.54
8.44
8.43
8.43
8.41
8.41
8.41
7.58
7.57
7.55
7.53
7.35
7.35
7.35
7.33
7.33
7.33
7.32
7.31
7.31
7.30
F
N
N
N
F
F
2l
3.00
2.90
3.16
3.07
f1 (ppm)

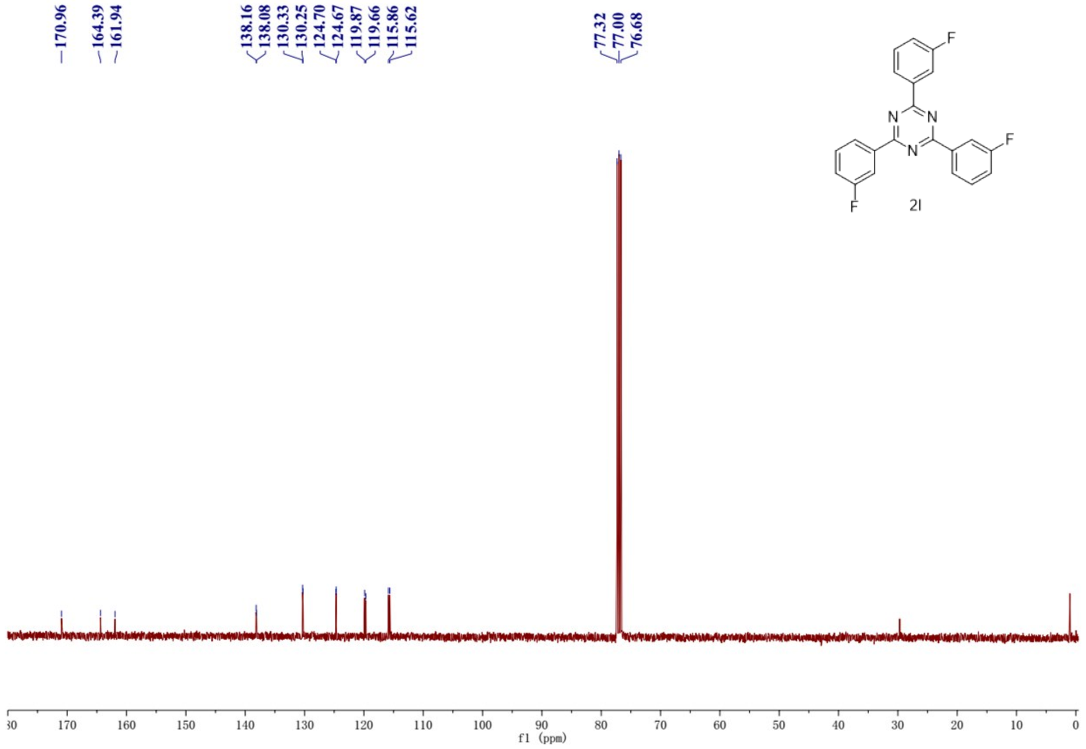
170.96
164.39
161.94
138.16
138.08
130.33
130.25
124.70
124.67
119.87
119.66
115.86
115.62
77.32
77.00
76.68
F
N
N
N
F
F
2l
170
160
150
140
130
120
110
100
90
80
70
60
50
40
30
20
10
0
f1 (ppm)

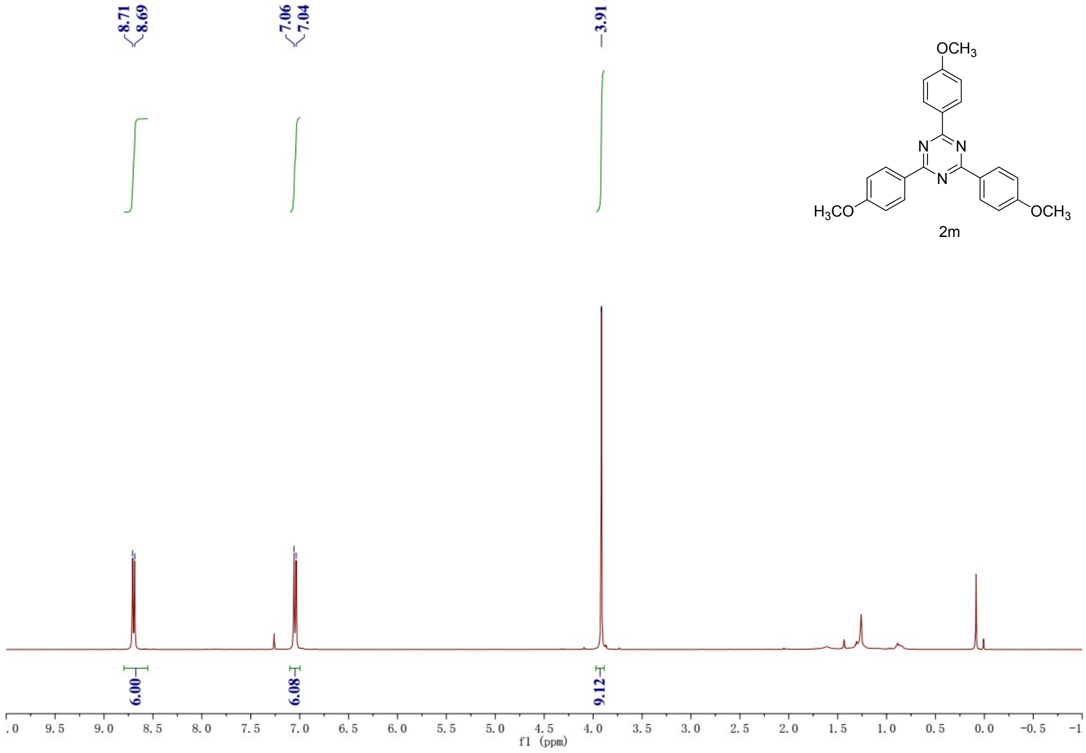
8.71
8.69
7.06
7.04
3.91
OCH3
N
N
N
H3CO
OCH3
2m
6.00
6.08
9.12
9.5
9.0
8.5
8.0
7.5
7.0
6.5
6.0
5.5
5.0
4.5
4.0
3.5
3.0
2.5
2.0
1.5
1.0
0.5
0.0
-0.5
f1 (ppm)

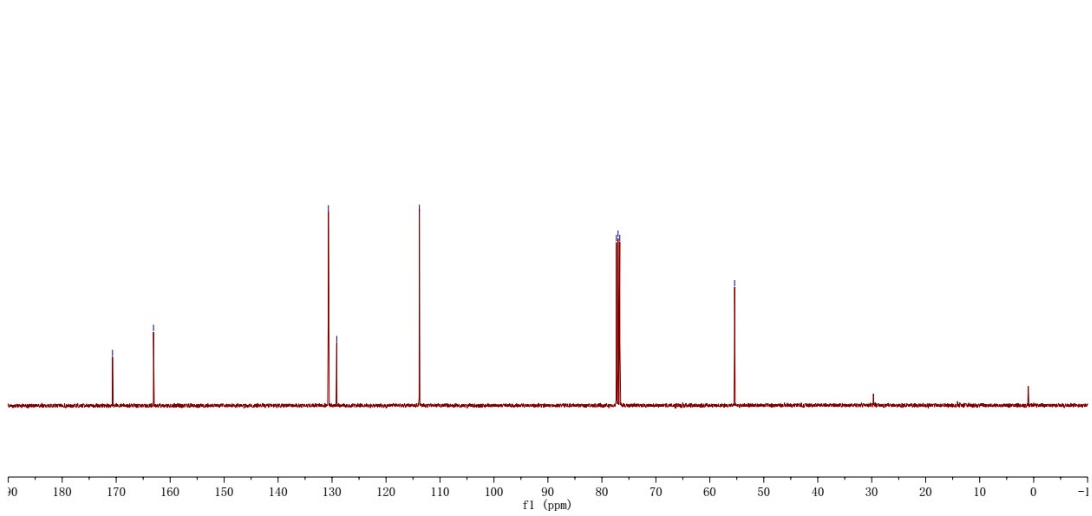

170.68
163.08
130.68
129.13
113.82
77.32
77.00
76.68
55.41
180 170 160 150 140 130 120 110 100 90 80 70 60 50 40 30 20 10 0 -1
f1 (ppm)

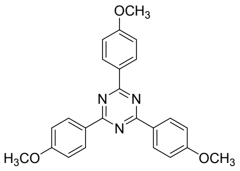

OCH3
N
N
N
H3CO
OCH3

2m

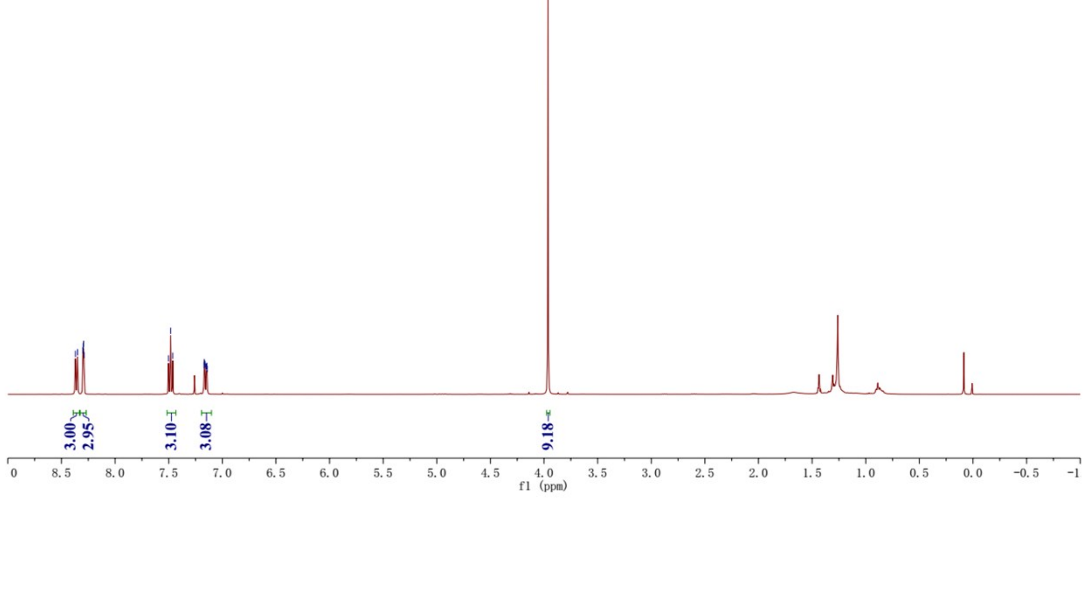

8.370
8.350
8.301
8.297
8.294
8.290
7.502
7.482
7.462
7.171
7.169
7.165
7.162
7.151
7.148
7.144
7.141
3.963
3.00
2.95
3.10
3.08
9.18
8.5 8.0 7.5 7.0 6.5 6.0 5.5 5.0 4.5 4.0 3.5 3.0 2.5 2.0 1.5 1.0 0.5 0.0 -0.5 -1
f1 (ppm)

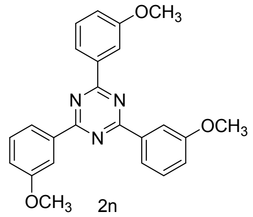

OCH3
N
N
N
OCH3
OCH3

2n

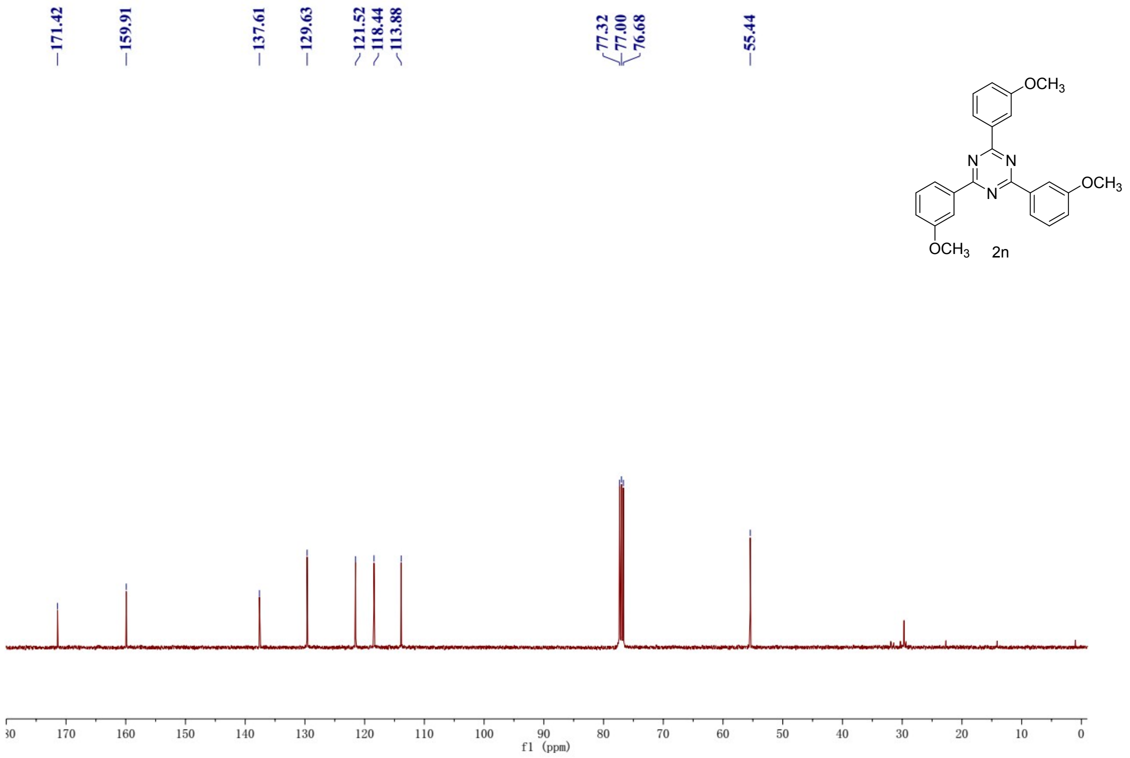
171.42
159.91
137.61
129.63
121.52
118.44
113.88
77.32
77.00
76.68
55.44
OCH3
N
OCH3
OCH3
2n
f1 (ppm)

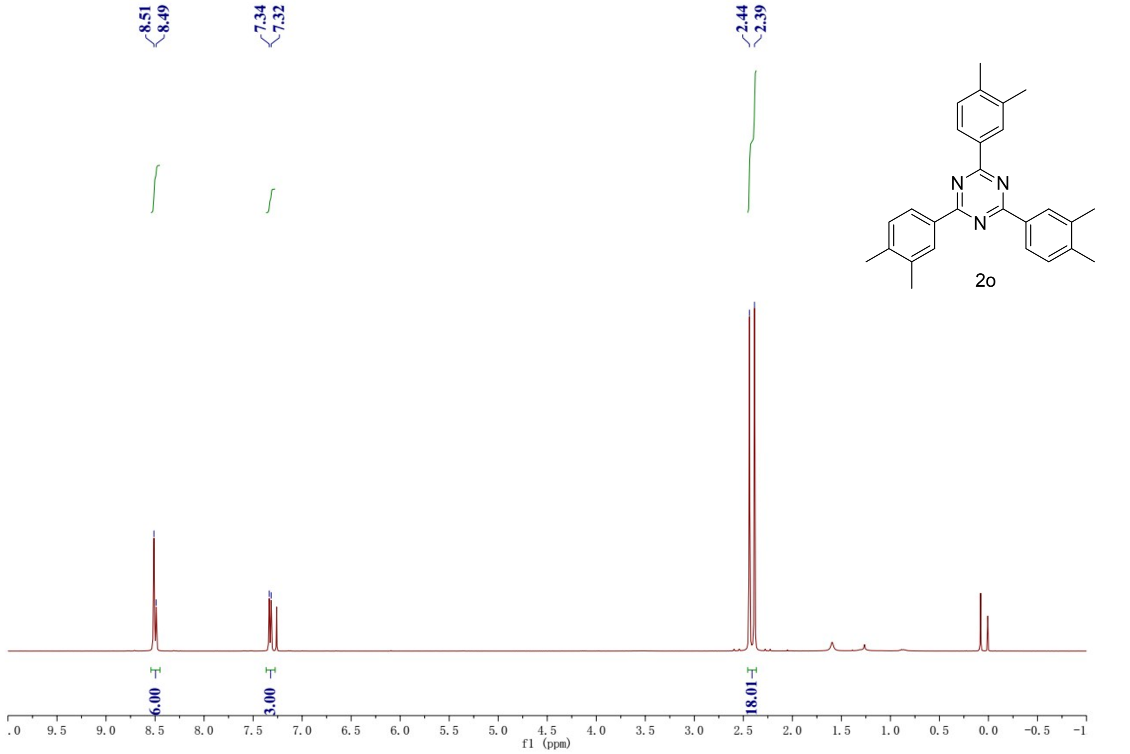
8.51
8.49
7.34
7.32
2.44
2.39
N
2o
6.00
3.00
18.01
f1 (ppm)

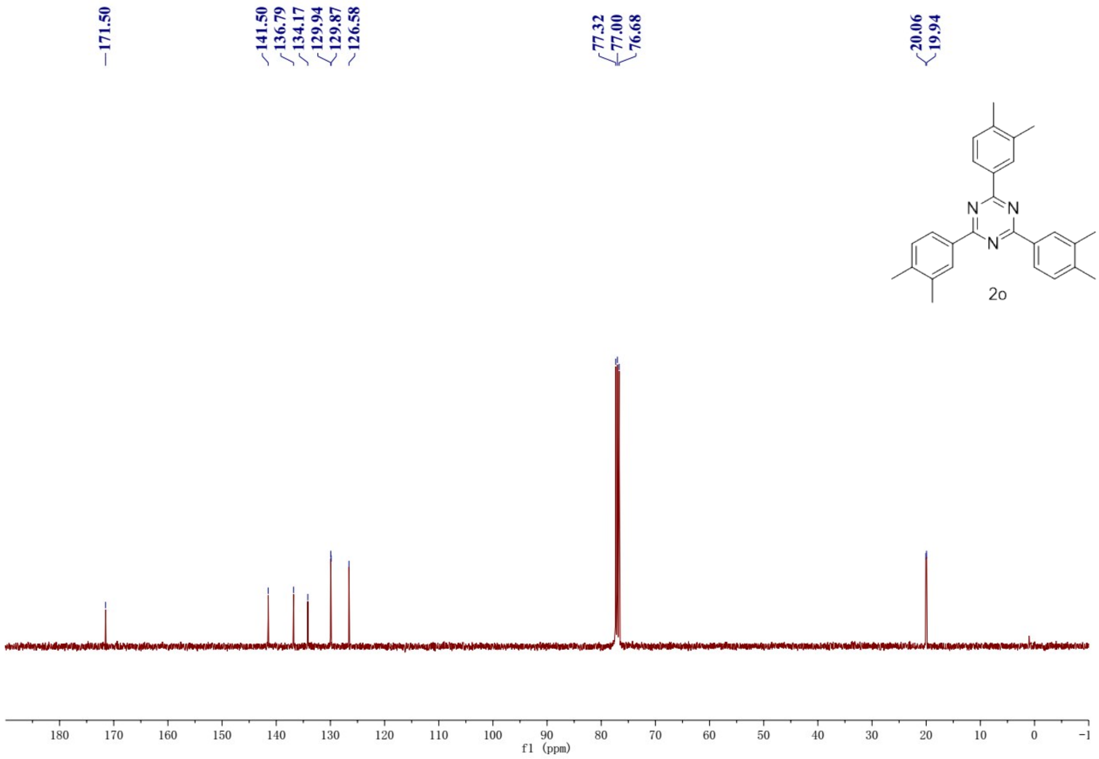
171.50
141.50
136.79
134.17
129.94
129.87
126.58
77.32
77.00
76.68
20.06
19.94
N
N
N
2o
180 170 160 150 140 130 120 110 100 90 80 70 60 50 40 30 20 10 0 -1
f1 (ppm)

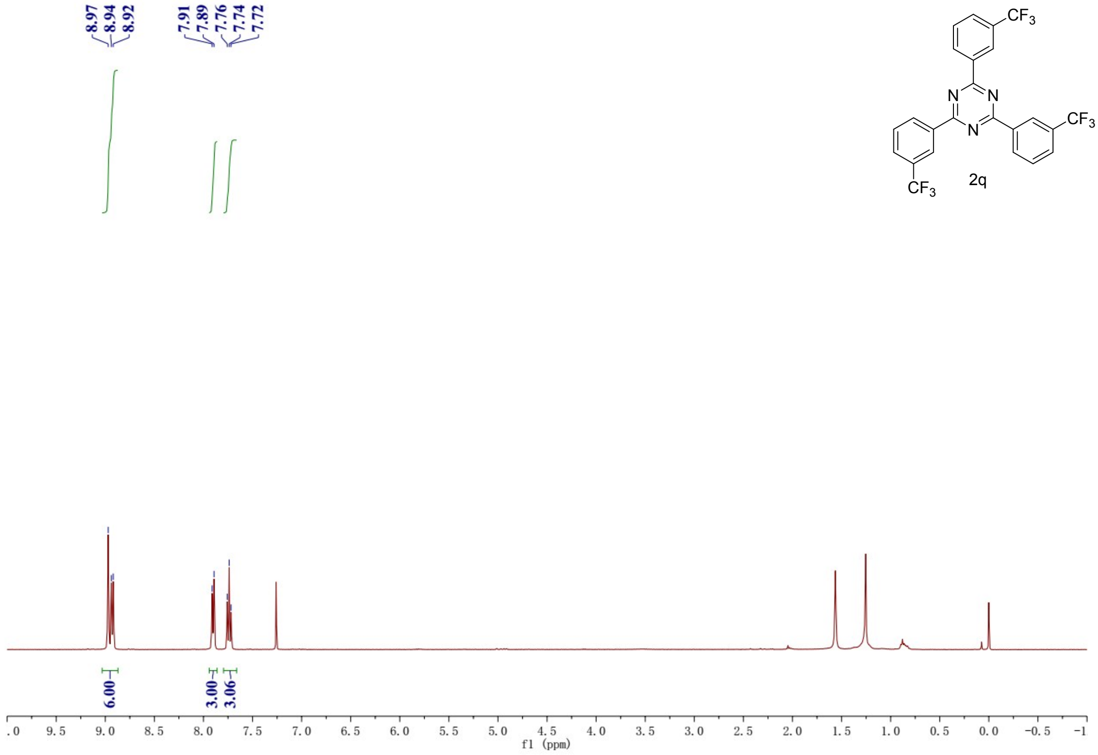
8.97
8.94
8.92
7.91
7.89
7.76
7.74
7.72
CF_3
CF_3
CF_3
N
N
N
2q
6.00
3.00
3.06
.0 9.5 9.0 8.5 8.0 7.5 7.0 6.5 6.0 5.5 5.0 4.5 4.0 3.5 3.0 2.5 2.0 1.5 1.0 0.5 0.0 -0.5 -1
f1 (ppm)

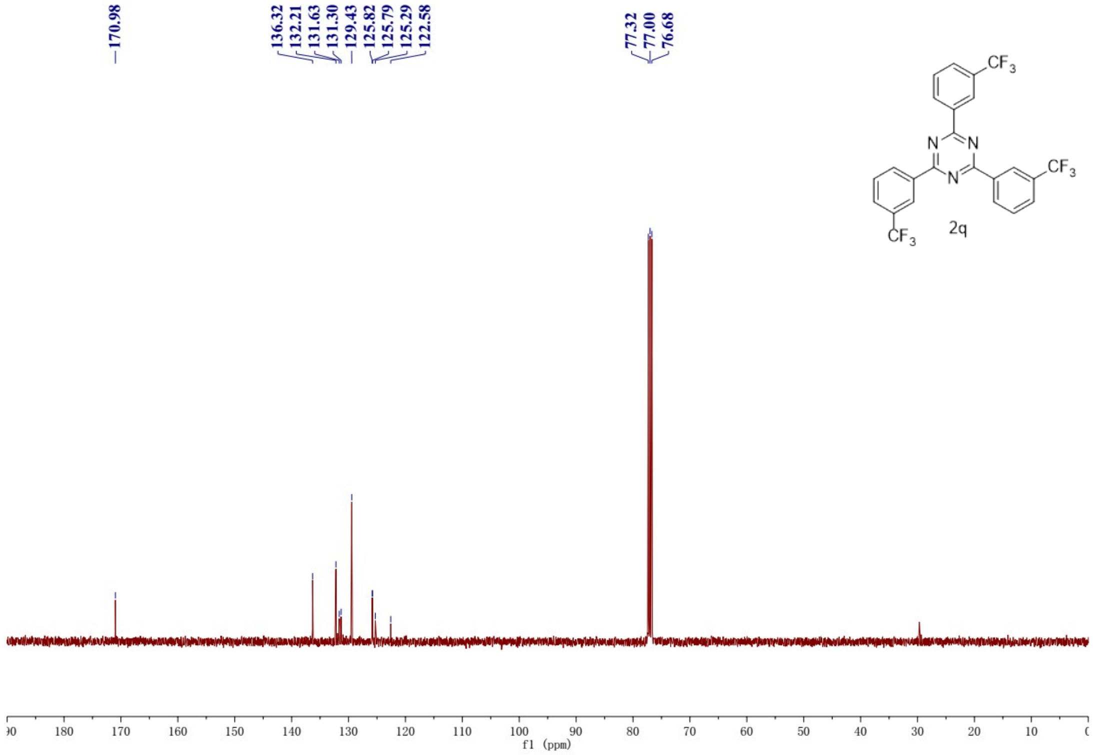
170.98
136.32
132.21
131.63
131.30
129.43
125.82
125.79
125.29
122.58
77.32
77.00
76.68
2q
f1 (ppm)

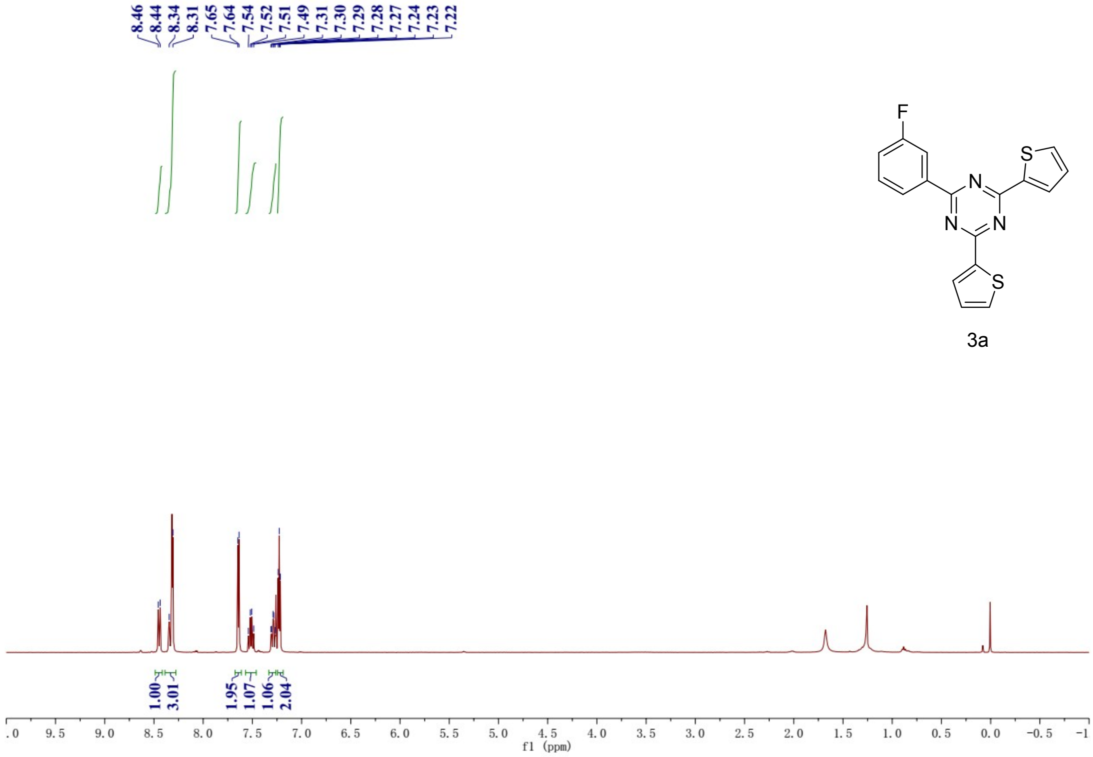
8.46
8.44
8.34
8.31
7.65
7.64
7.54
7.52
7.51
7.49
7.31
7.30
7.29
7.28
7.27
7.24
7.23
7.22
3a
1.00
3.01
1.95
1.07
1.06
2.04
f1 (ppm)

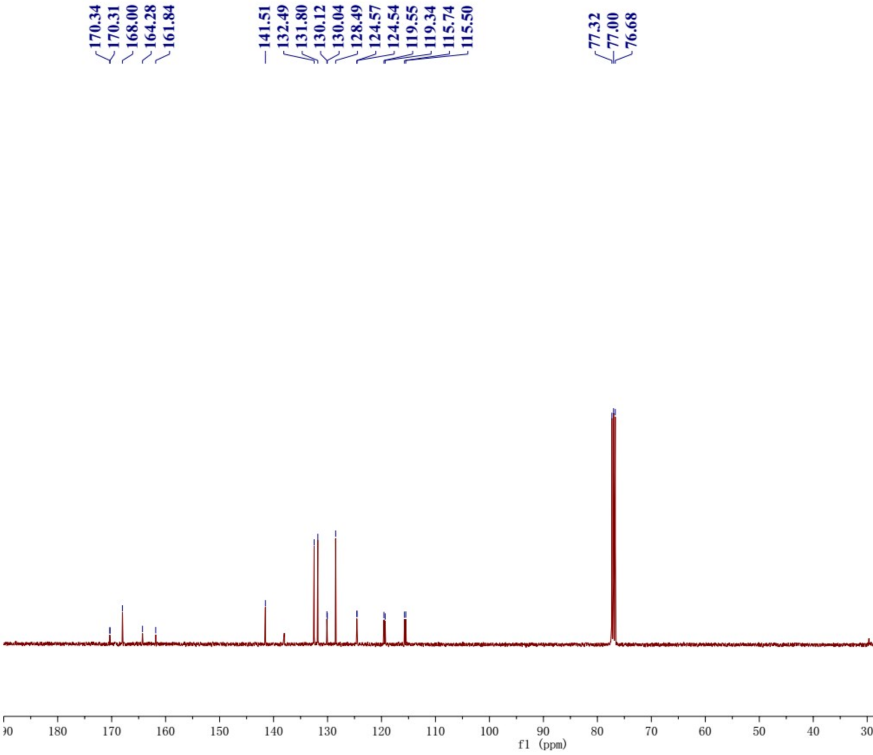
170.34
170.31
168.00
164.28
161.84
141.51
132.49
131.80
130.12
130.04
128.49
124.57
124.54
119.55
119.34
115.74
115.50
77.32
77.00
76.68
f1 (ppm)

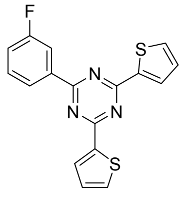
F
N
N
N
S
S

3a

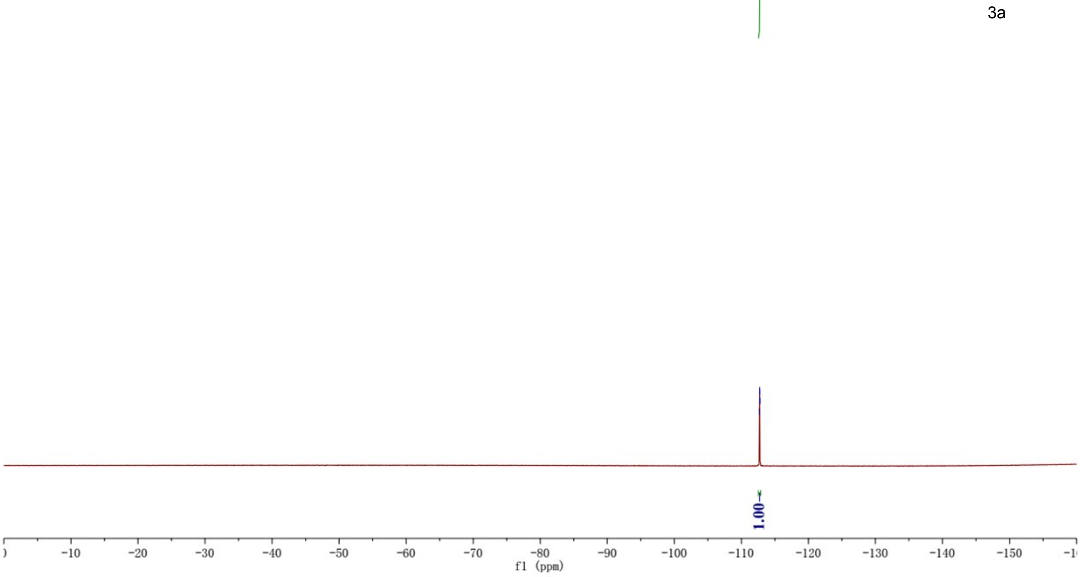
-112.71
-112.73
-112.74
-112.75
-112.77
1.00
f1 (ppm)

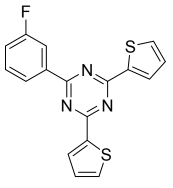
F
N
N
N
S
S

3a

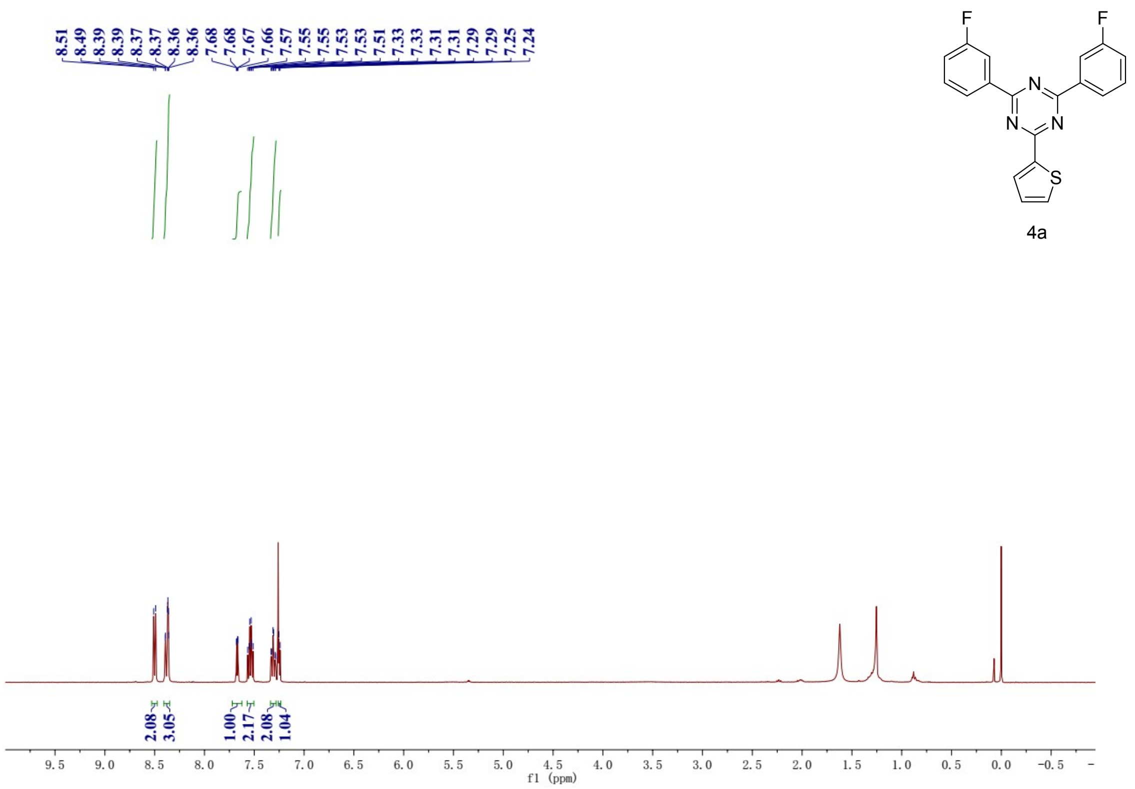
8.51
8.49
8.39
8.39
8.37
8.37
8.36
8.36
7.68
7.68
7.67
7.66
7.57
7.55
7.55
7.53
7.53
7.51
7.33
7.33
7.31
7.31
7.29
7.29
7.25
7.24
2.08
3.05
1.00
2.17
2.08
1.04
9.5
9.0
8.5
8.0
7.5
7.0
6.5
6.0
5.5
5.0
4.5
4.0
3.5
3.0
2.5
2.0
1.5
1.0
0.5
0.0
-0.5
f1 (ppm)

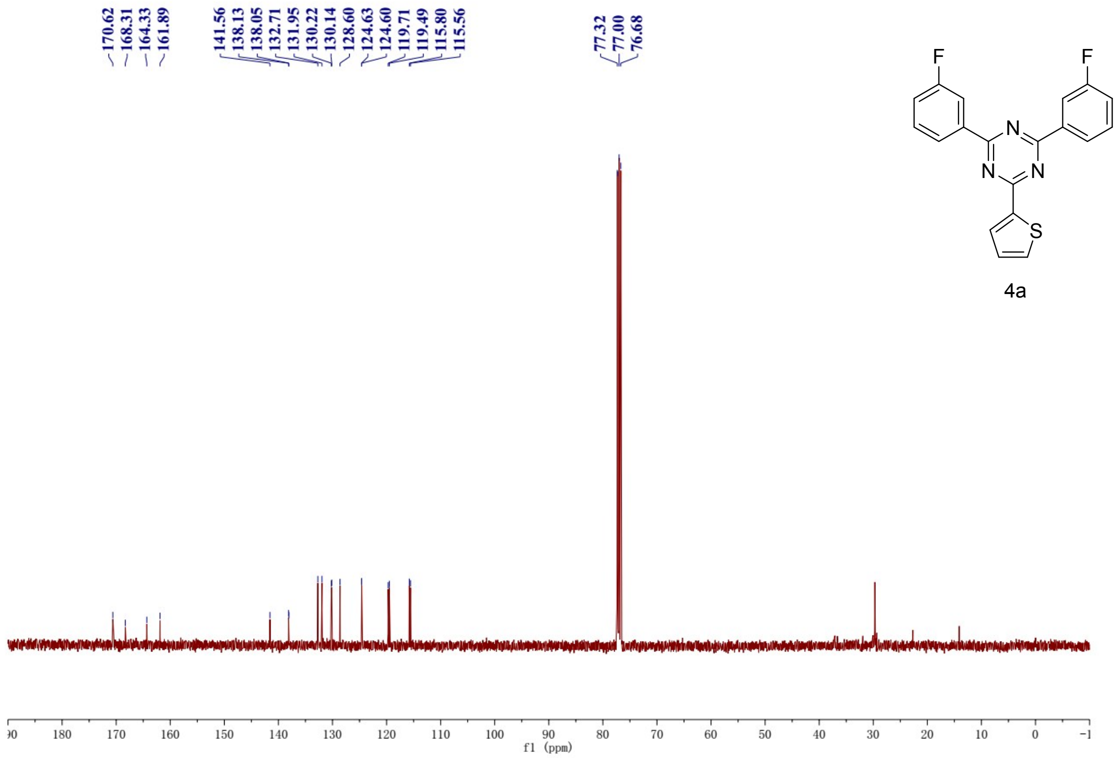
170.62
168.31
164.33
161.89
141.56
138.13
138.05
132.71
131.95
130.22
130.14
128.60
124.63
124.60
119.71
119.49
115.80
115.56
77.32
77.00
76.68
180
170
160
150
140
130
120
110
100
90
80
70
60
50
40
30
20
10
0
f1 (ppm)
4a

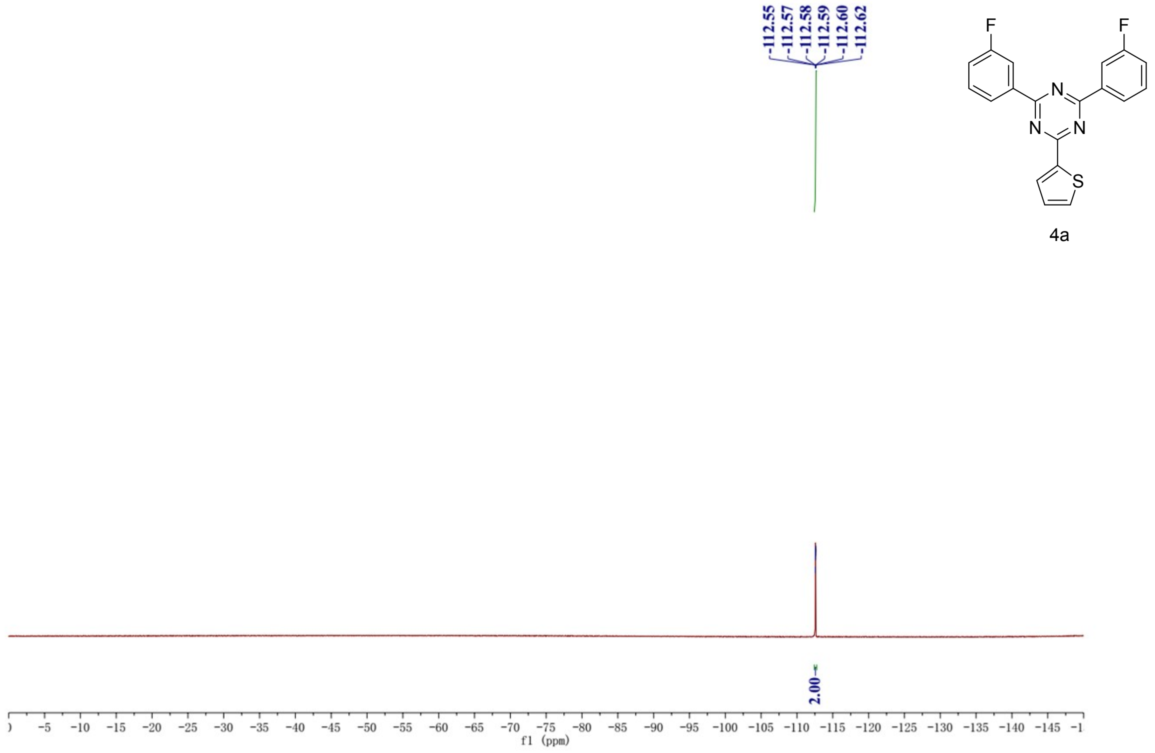
-112.55
-112.57
-112.58
-112.59
-112.60
-112.62
2.00
0 -5 -10 -15 -20 -25 -30 -35 -40 -45 -50 -55 -60 -65 -70 -75 -80 -85 -90 -95 -100 -105 -110 -115 -120 -125 -130 -135 -140 -145 -1
f1 (ppm)
F
F
N
N
N
S

4a

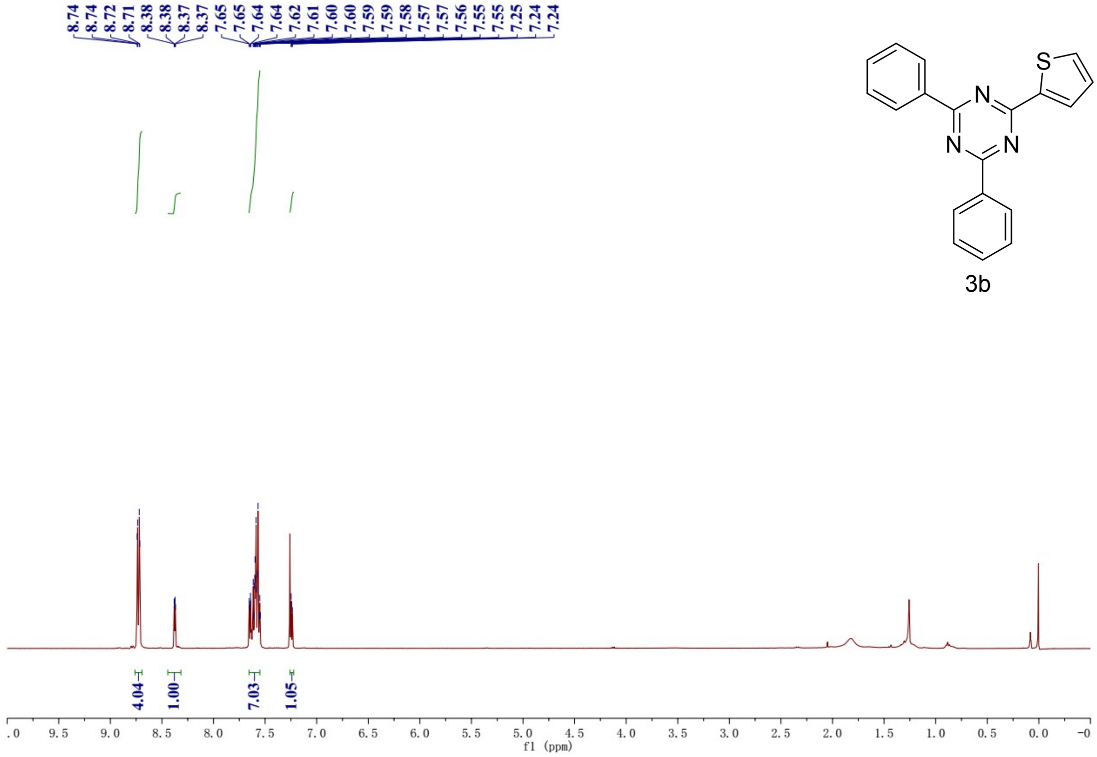
8.74
8.74
8.72
8.71
8.38
8.38
8.37
8.37
7.65
7.65
7.64
7.64
7.62
7.61
7.60
7.60
7.59
7.59
7.58
7.57
7.57
7.56
7.55
7.55
7.25
7.24
7.24
4.04
1.00
7.03
1.05
.0 9.5 9.0 8.5 8.0 7.5 7.0 6.5 6.0 5.5 5.0 4.5 4.0 3.5 3.0 2.5 2.0 1.5 1.0 0.5 0.0 -0
f1 (ppm)
S
N
N
N

3b

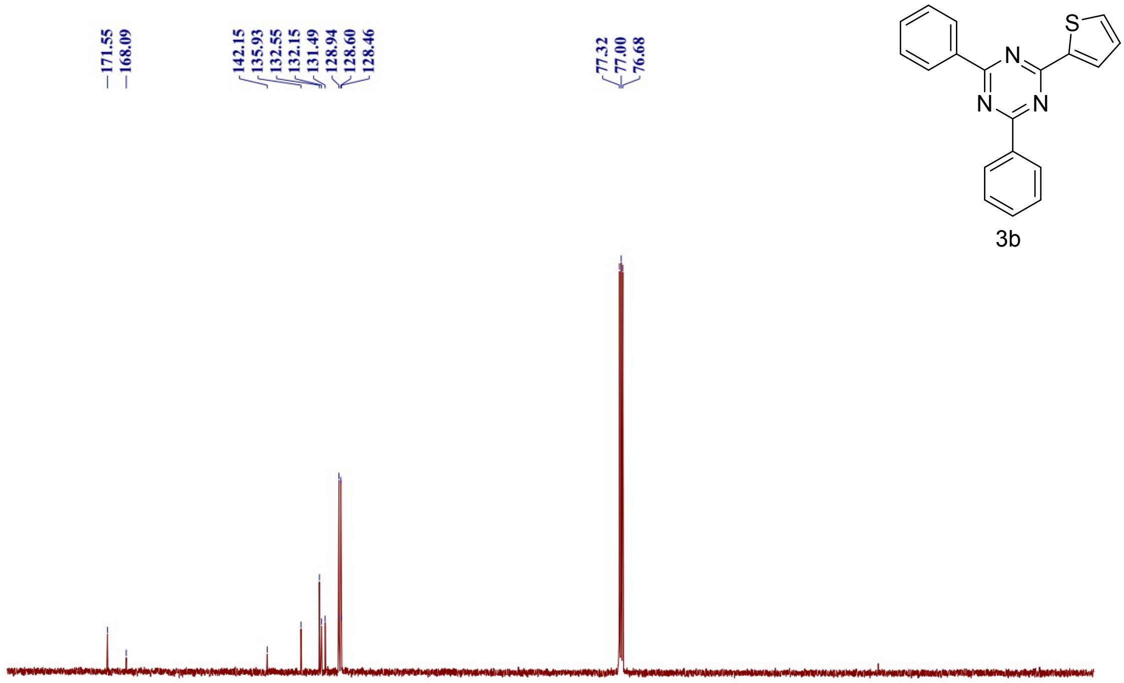
171.55
168.09
142.15
135.93
132.55
132.15
131.49
128.94
128.60
128.46
77.32
77.00
76.68

3b

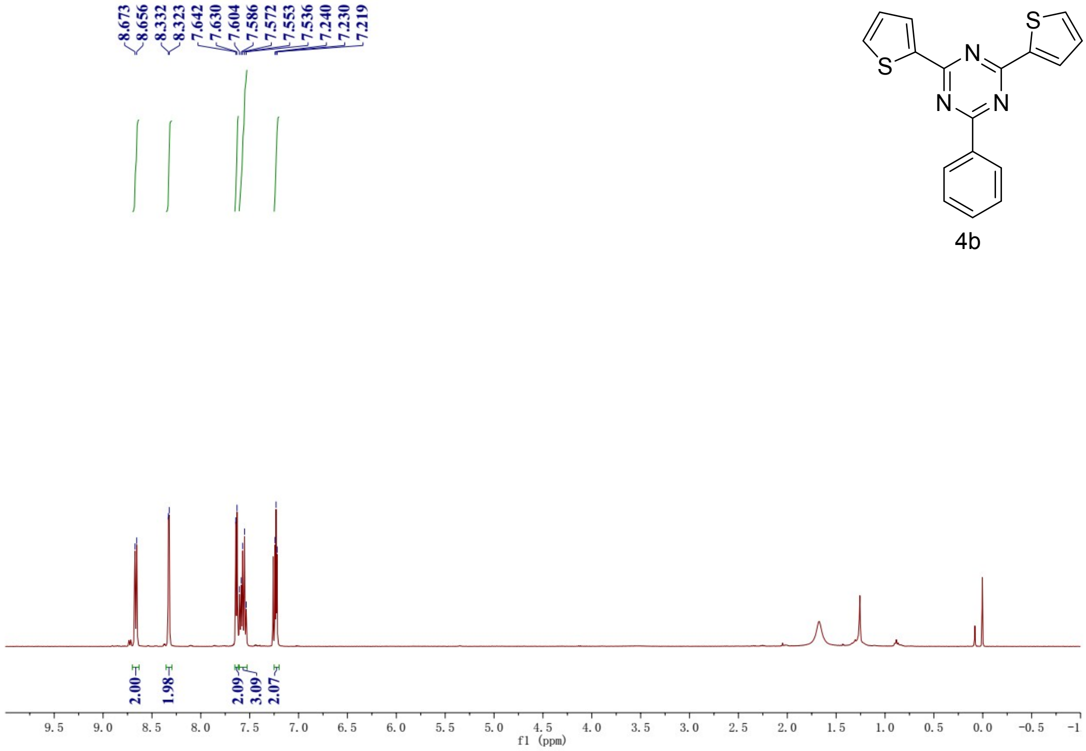
8.673
8.656
8.332
8.323
7.642
7.630
7.604
7.586
7.572
7.553
7.536
7.240
7.230
7.219
2.00
1.98
2.09
3.09
2.07
9.5 9.0 8.5 8.0 7.5 7.0 6.5 6.0 5.5 5.0 4.5 4.0 3.5 3.0 2.5 2.0 1.5 1.0 0.5 0.0 -0.5 -1
f1 (ppm)

4b

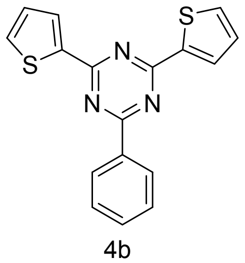

4b

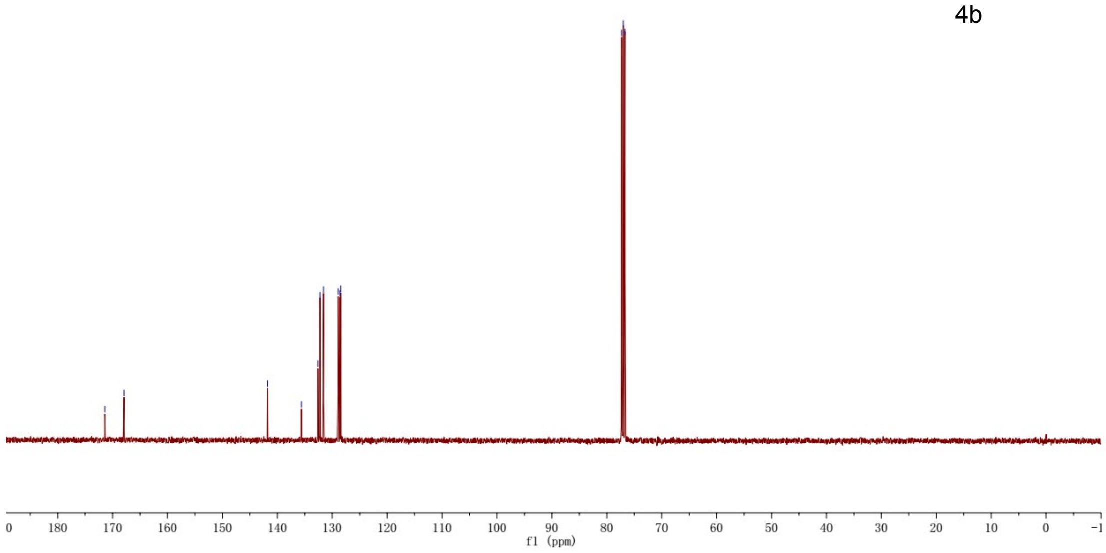

171.41
167.91
141.80
135.60
132.59
132.23
131.59
128.91
128.57
128.44
77.32
77.00
76.68
f1 (ppm)

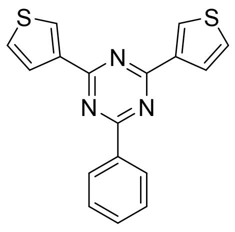

4c

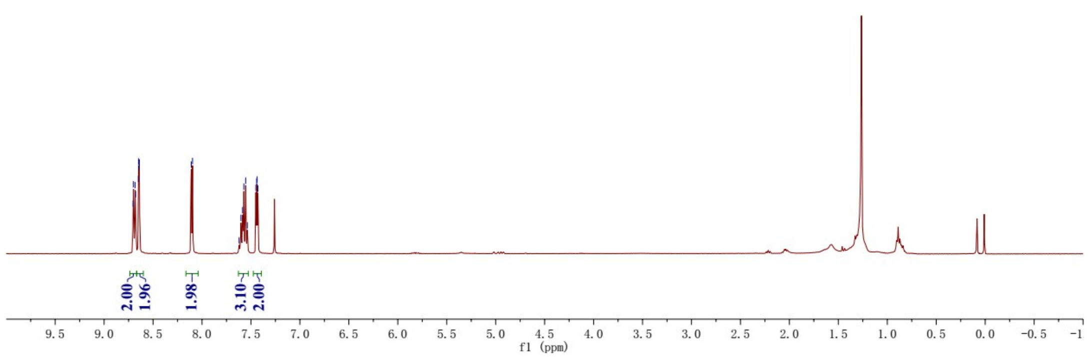

2.00
1.96
1.98
3.10
2.00
f1 (ppm)

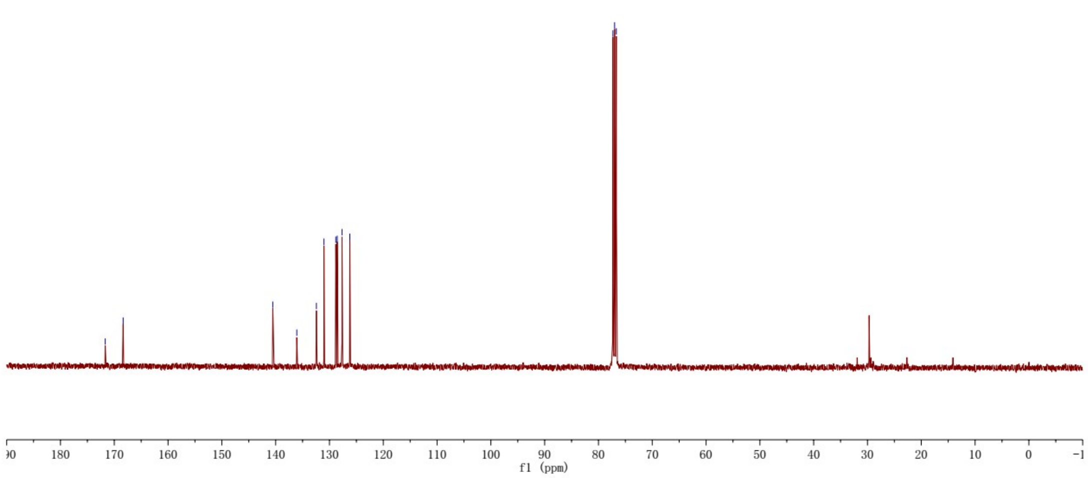

171.68
168.34
140.53
136.07
132.43
131.02
128.82
128.56
127.67
126.22
77.32
77.00
76.68
f1 (ppm)

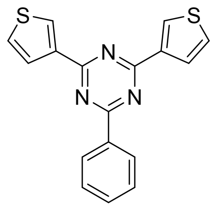

S
N

4c

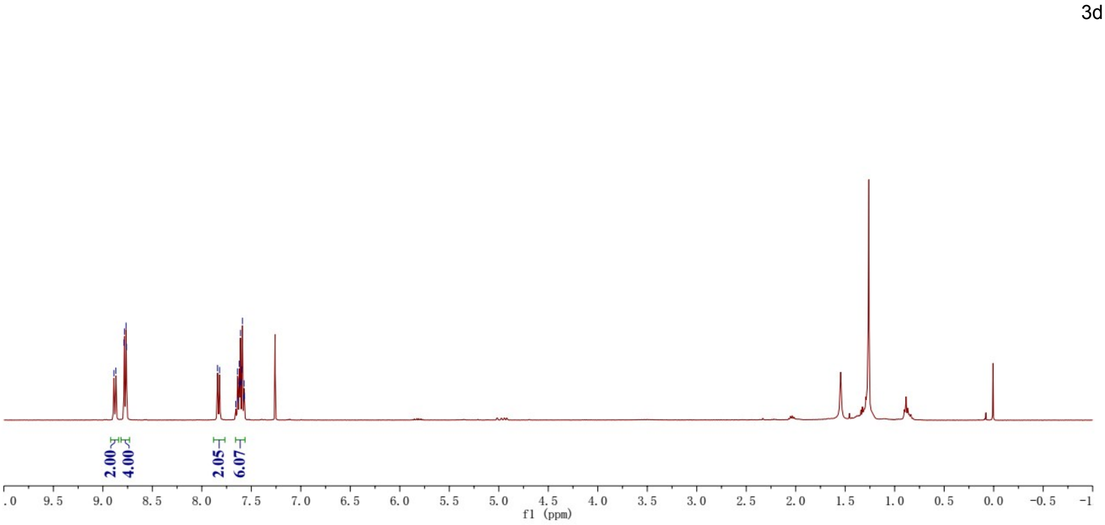

8.889
8.868
8.786
8.782
8.766
8.761
7.841
7.821
7.657
7.639
7.632
7.626
7.622
7.617
7.609
7.605
7.595
7.590
7.578
7.573
7.569
2.00
4.00
2.05
6.07
f1 (ppm)

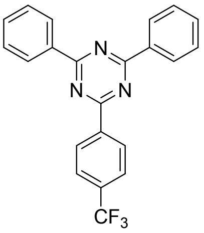

N
CF_3

3d

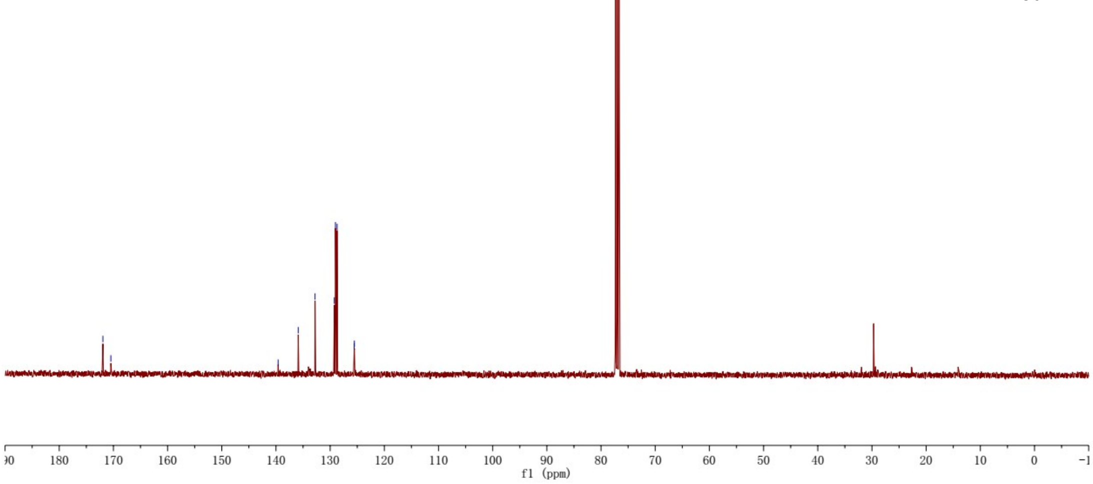
171.94
170.47
139.59
135.89
132.79
129.23
129.02
128.72
125.57
125.53
77.32
77.00
76.68
f1 (ppm)

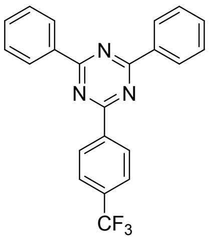

3d

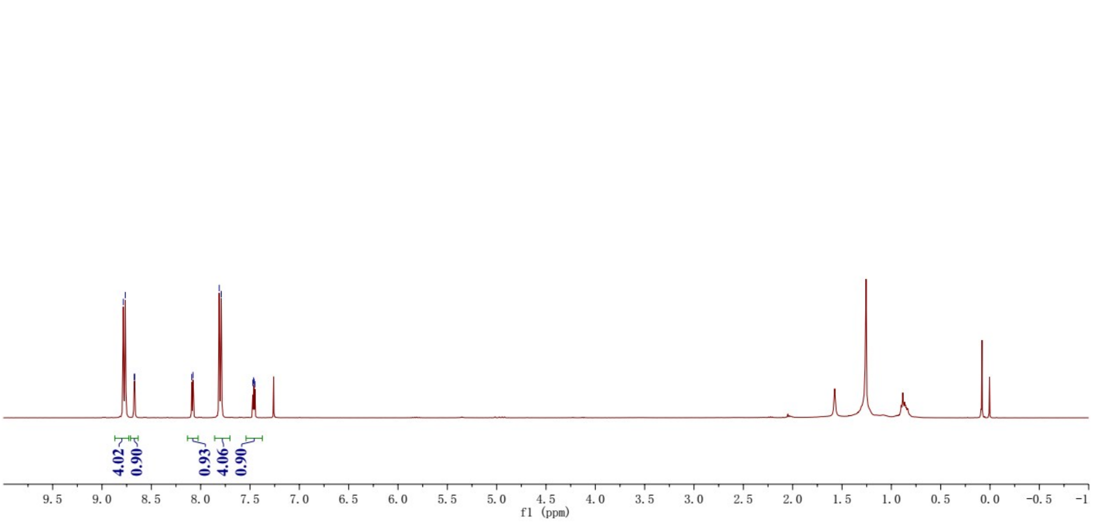
8.784
8.764
8.674
8.669
8.090
8.078
7.812
7.792
7.471
7.463
7.458
7.451
4.02
0.90
0.93
4.06
0.90
f1 (ppm)

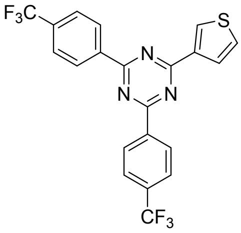

4e

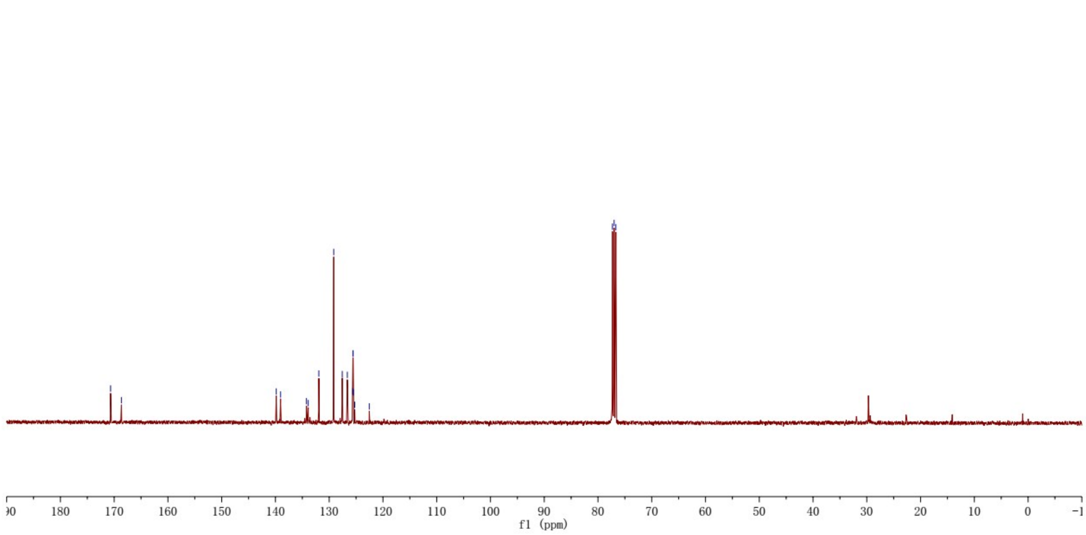
170.66
168.65
139.85
139.04
134.23
133.91
131.93
129.16
127.57
126.63
125.63
125.59
125.55
125.51
125.25
122.54
77.32
77.00
76.68
f1 (ppm)

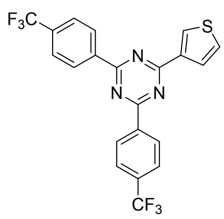
F3C
N
N
N
S
CF3
4e

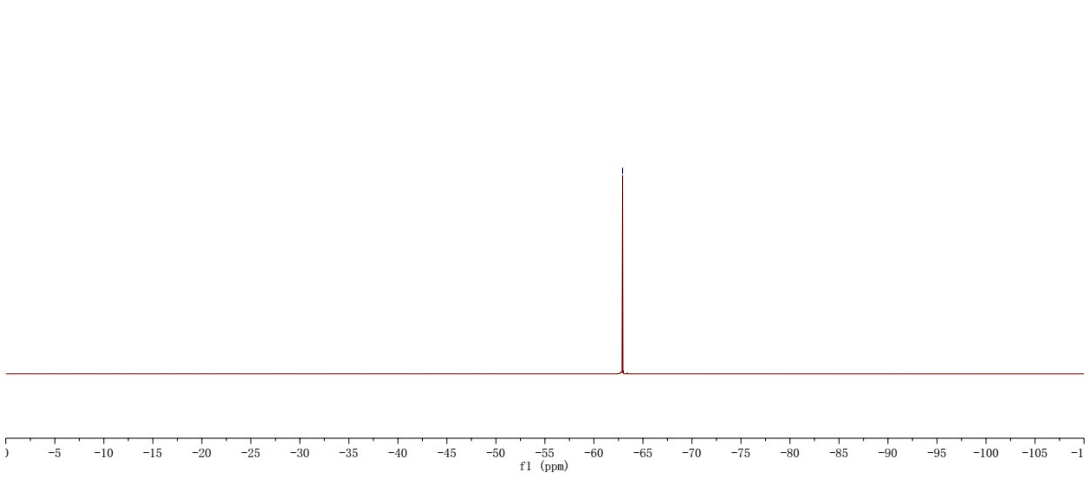
-62.92
f1 (ppm)

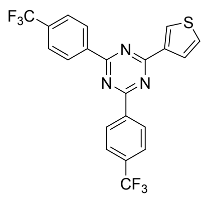
F3C
N
N
N
S
CF3
4e

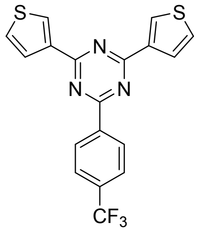
S
S
N
N
N
CF3
3e

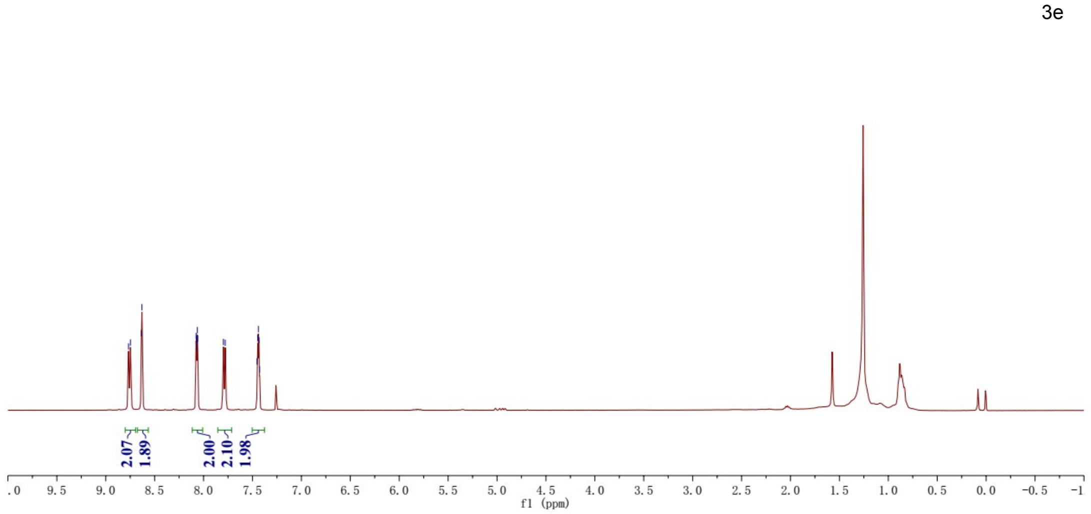
8.767
8.748
8.636
8.630
8.076
8.073
8.071
8.066
8.064
8.061
7.797
7.779
7.453
7.446
7.440
7.433
7.427
2.07
1.89
2.00
2.10
1.98
.0 9.5 9.0 8.5 8.0 7.5 7.0 6.5 6.0 5.5 5.0 4.5 4.0 3.5 3.0 2.5 2.0 1.5 1.0 0.5 0.0 -0.5 -1
f1 (ppm)

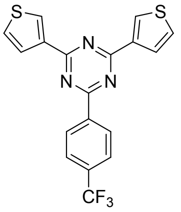
S
S
N
N
N
CF3
3e

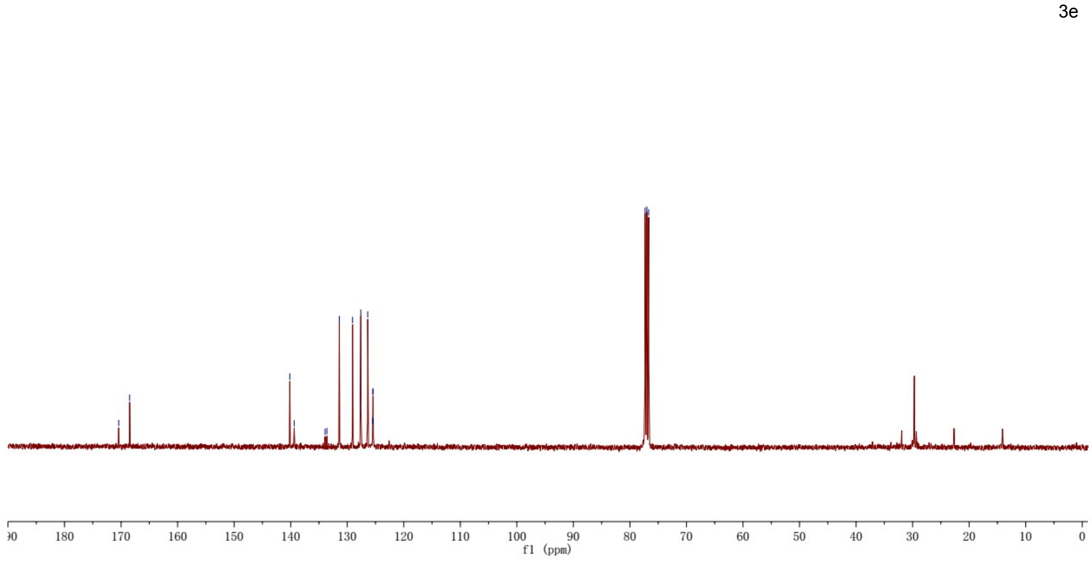
170.42
168.48
140.16
139.38
133.92
133.59
131.40
129.06
127.59
126.38
125.50
125.47
125.43
125.40
77.32
77.00
76.68
180 170 160 150 140 130 120 110 100 90 80 70 60 50 40 30 20 10 0
f1 (ppm)

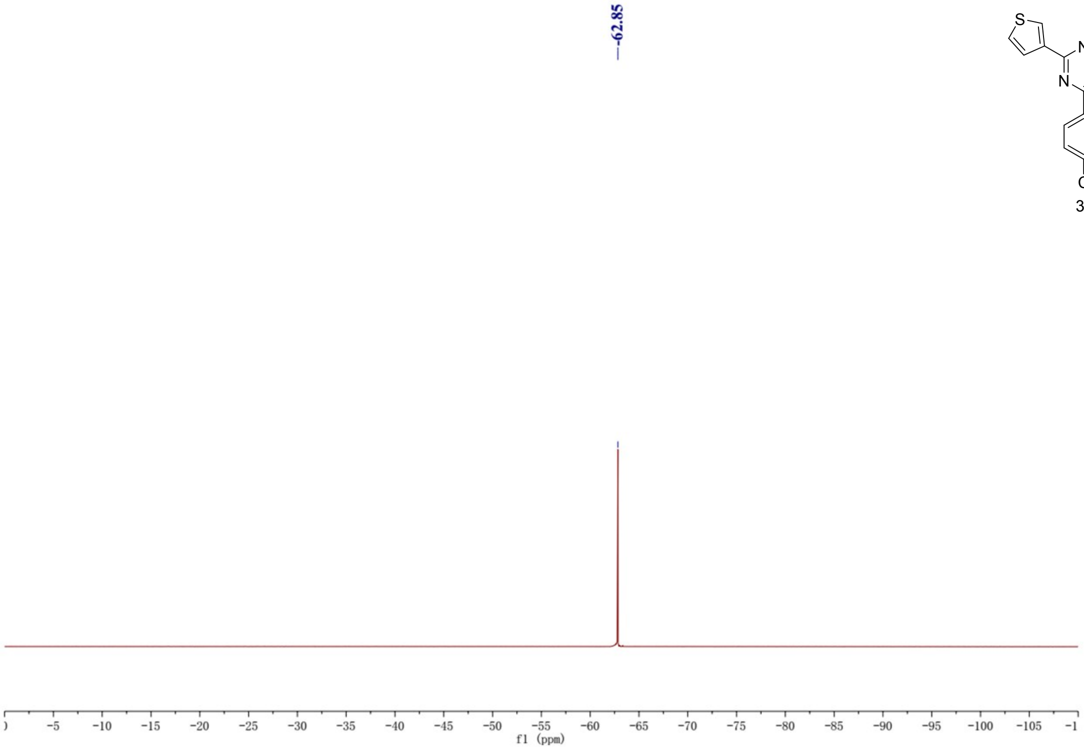
-62.85
f1 (ppm)

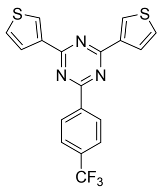

3e

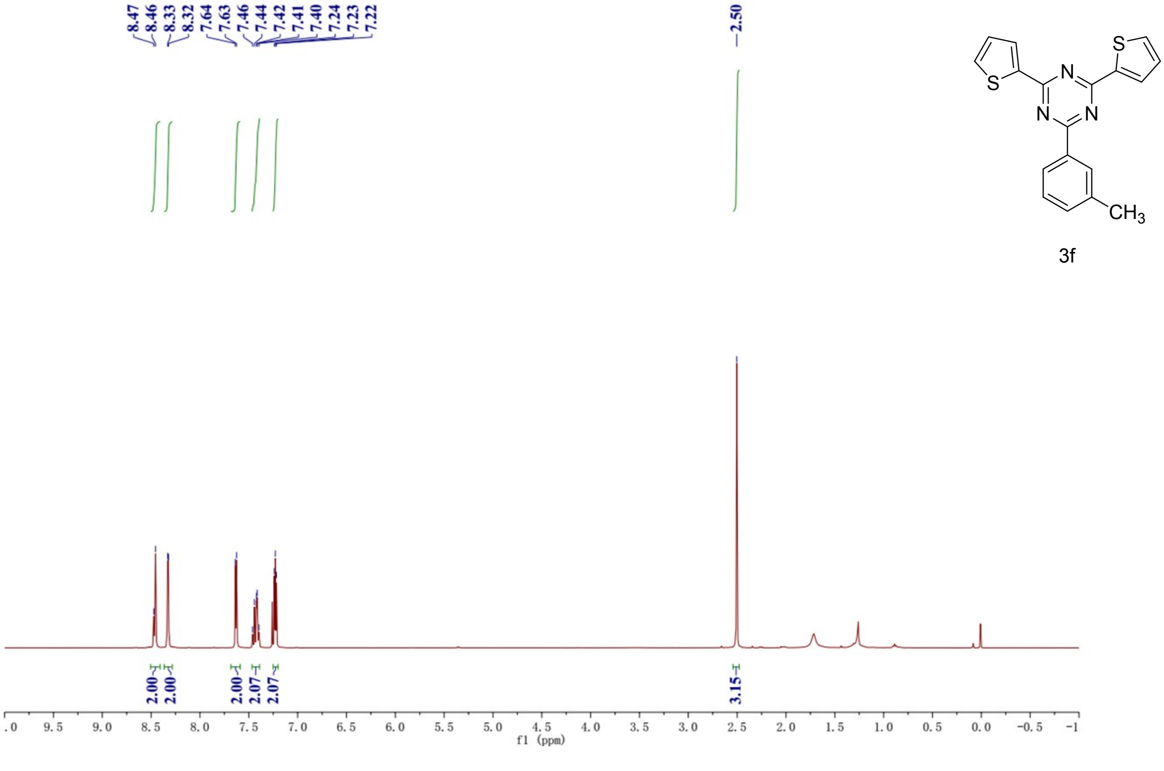
8.47
8.46
8.33
8.32
7.64
7.63
7.46
7.44
7.42
7.41
7.40
7.24
7.23
7.22
2.50
2.00
2.00
2.00
2.07
2.07
3.15
f1 (ppm)
CH3

3f

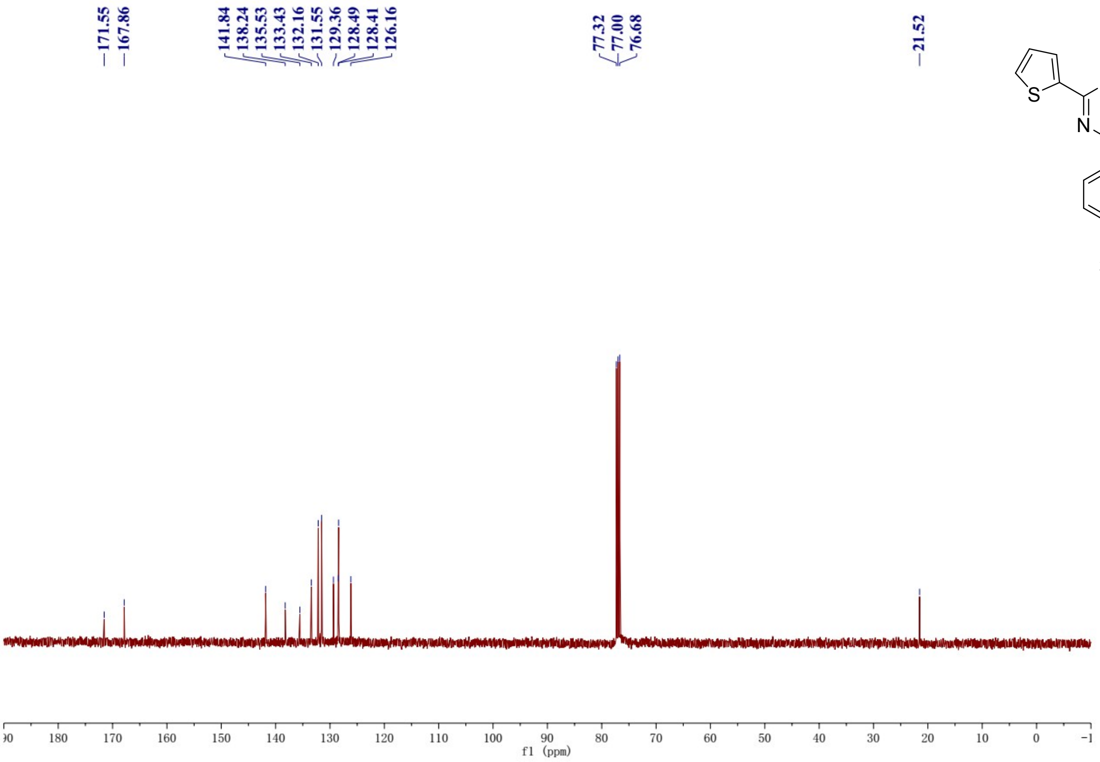
171.55
167.86
141.84
138.24
135.53
133.43
132.16
131.55
129.36
128.49
128.41
126.16
77.32
77.00
76.68
21.52
f1 (ppm)

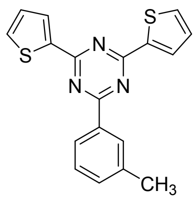
S
N
S
N
N
CH3

3f

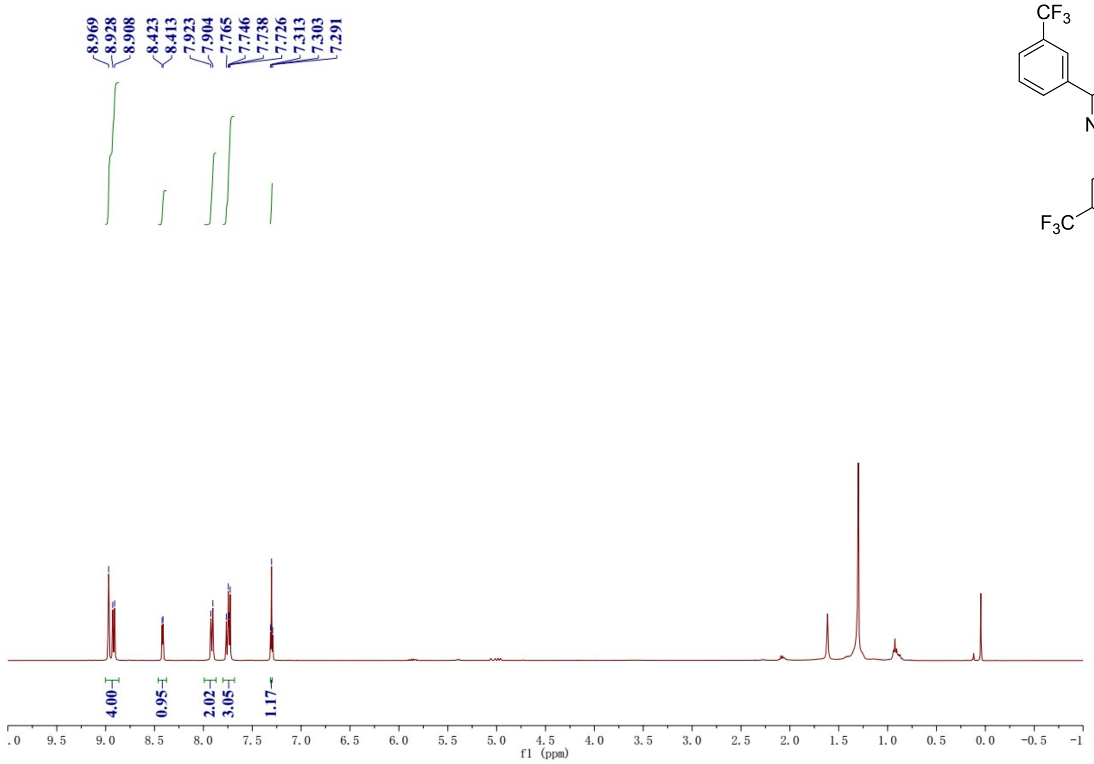
8.969
8.928
8.908
8.423
8.413
7.923
7.904
7.765
7.746
7.738
7.726
7.313
7.303
7.291
4.00
0.95
2.02
3.05
1.17
f1 (ppm)

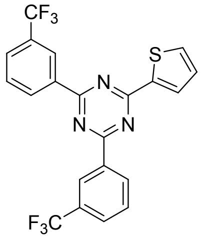
CF3
N
S
N
N
F3C

4g

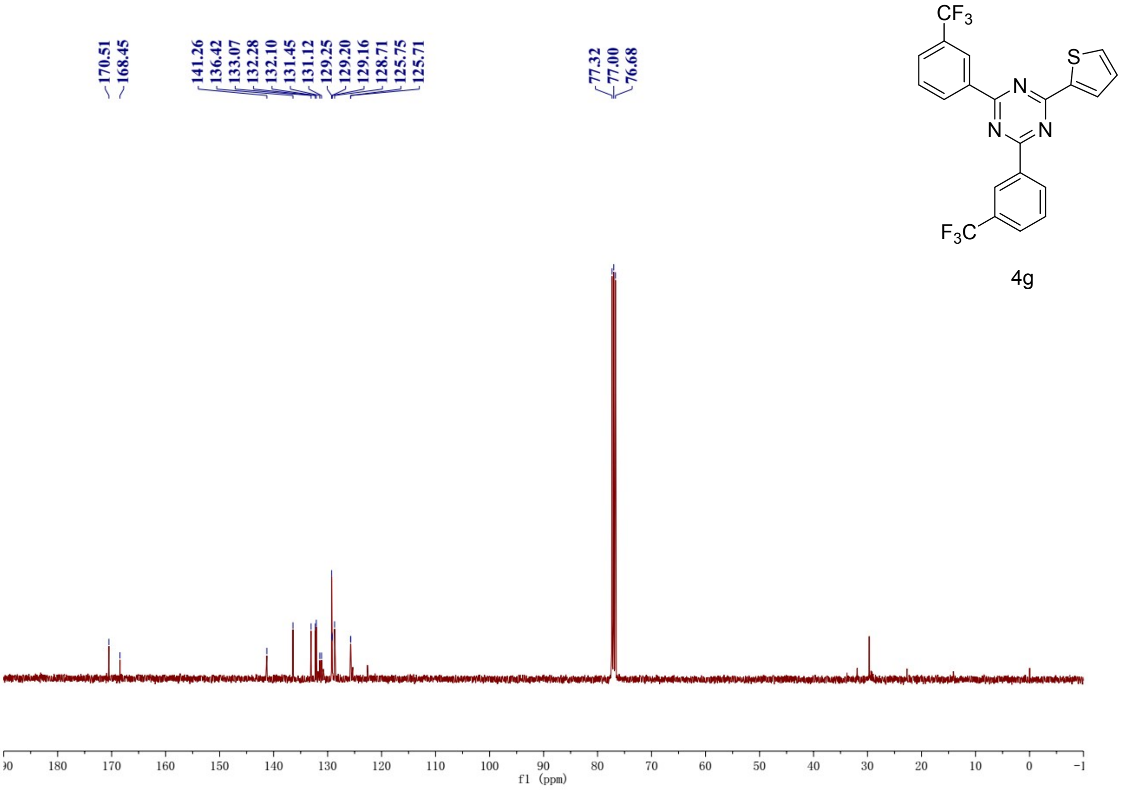
170.51
168.45
141.26
136.42
133.07
132.28
132.10
131.45
131.12
129.25
129.20
129.16
128.71
125.75
125.71
77.32
77.00
76.68
f1 (ppm)
CF3
S
N
N
N
F3C

4g

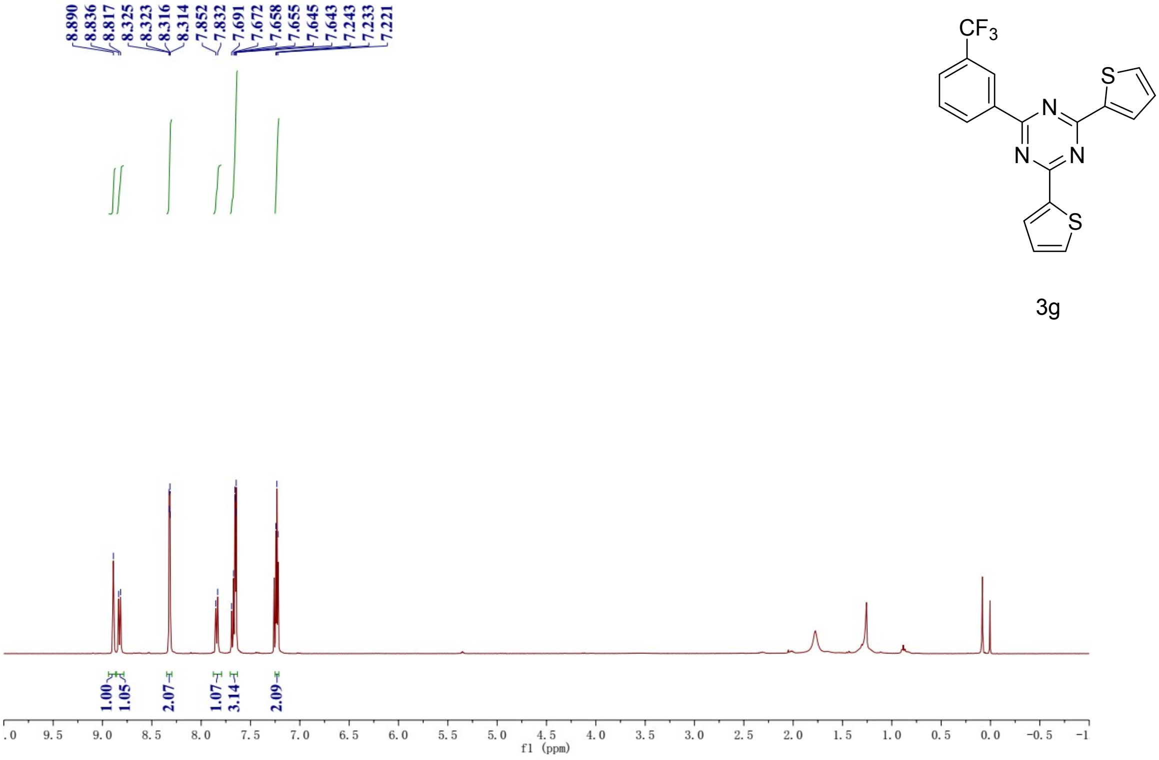
8.890
8.836
8.817
8.325
8.323
8.316
8.314
7.852
7.832
7.691
7.672
7.658
7.655
7.645
7.643
7.243
7.233
7.221
1.00
1.05
2.07
1.07
3.14
2.09
f1 (ppm)
CF3
S
N
N
N
S

3g

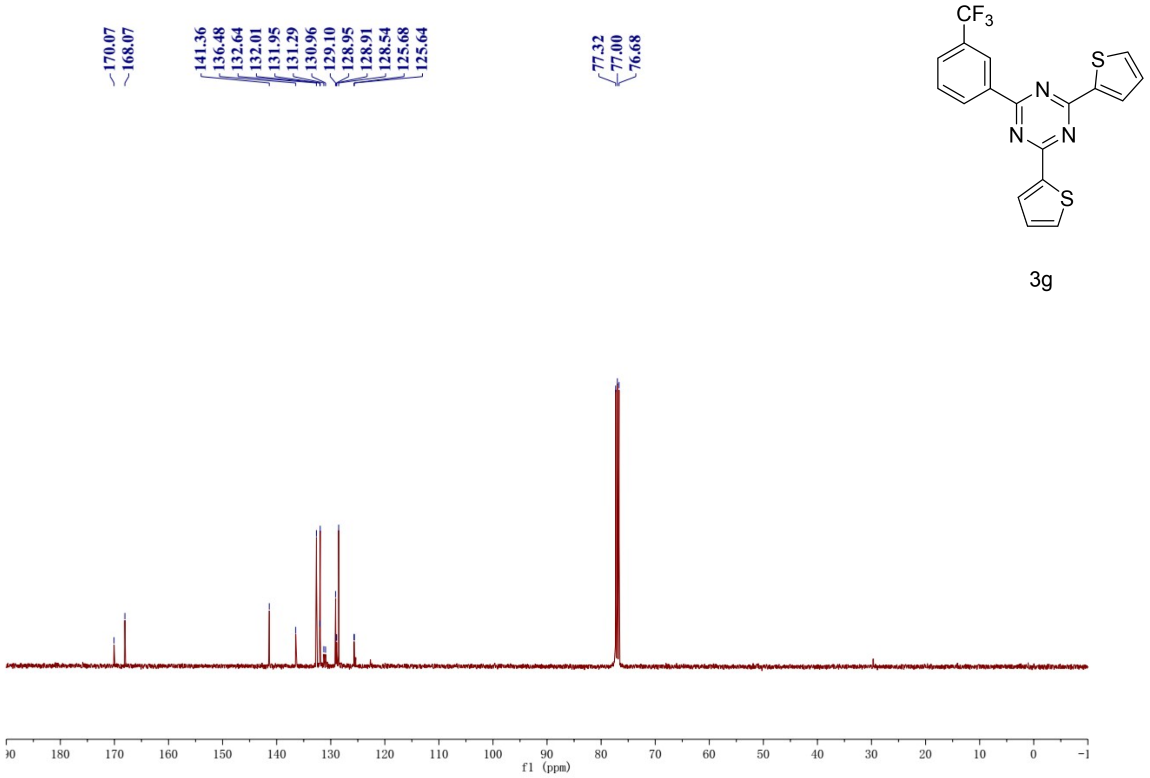
170.07
168.07
141.36
136.48
132.64
132.01
131.95
131.29
130.96
129.10
128.95
128.91
128.54
125.68
125.64
77.32
77.00
76.68
f1 (ppm)
CF3
N
N
N
S
S

3g

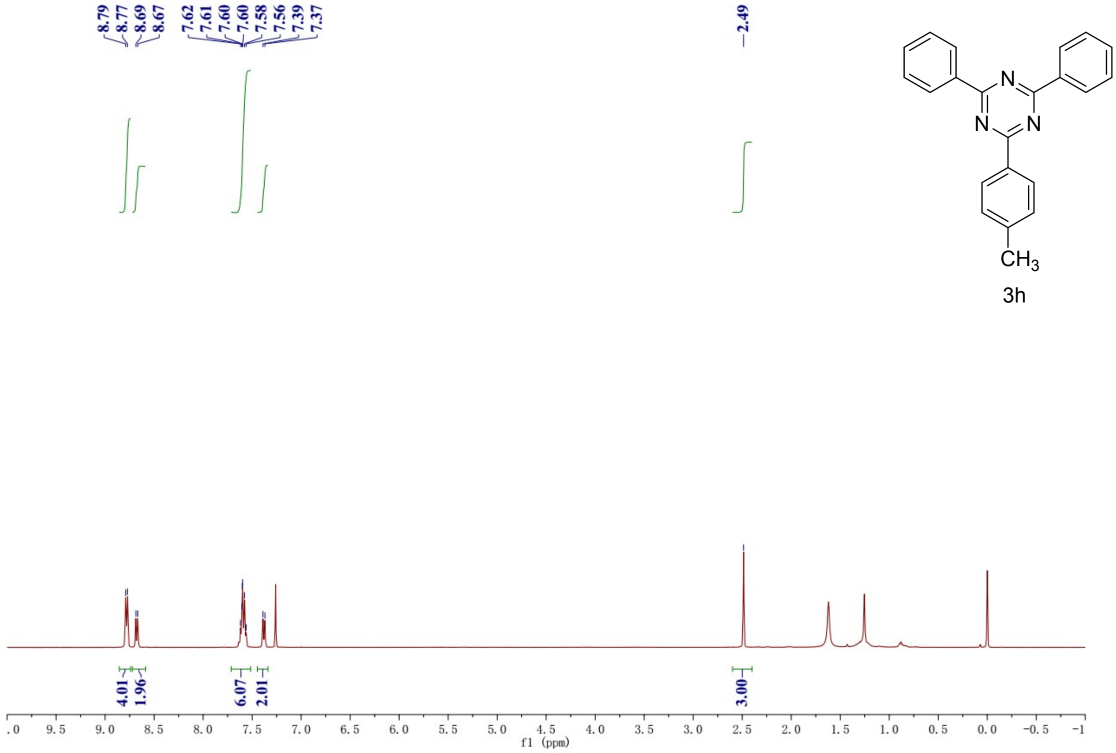
8.79
8.77
8.69
8.67
7.62
7.61
7.60
7.60
7.58
7.56
7.39
7.37
2.49
4.01
1.96
6.07
2.01
3.00
f1 (ppm)
N
N
N
CH3

3h

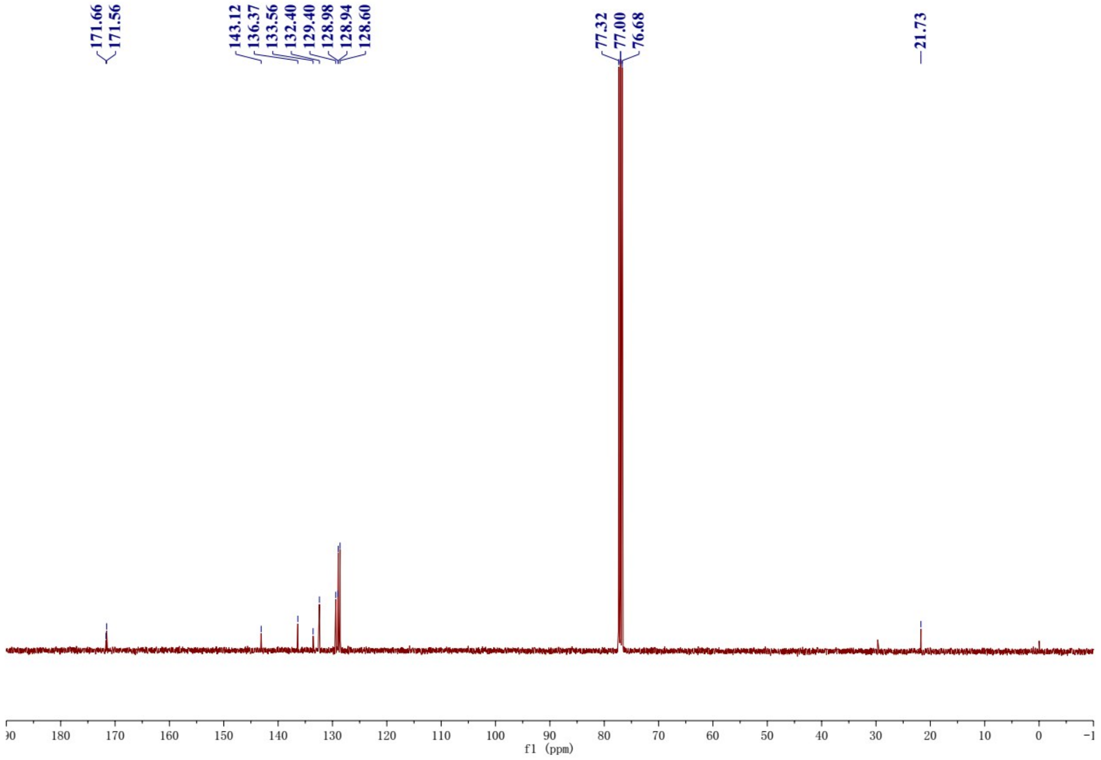
171.66
171.56
143.12
136.37
133.56
132.40
129.40
128.98
128.94
128.60
77.32
77.00
76.68
21.73
180 170 160 150 140 130 120 110 100 90 80 70 60 50 40 30 20 10 0
f1 (ppm)

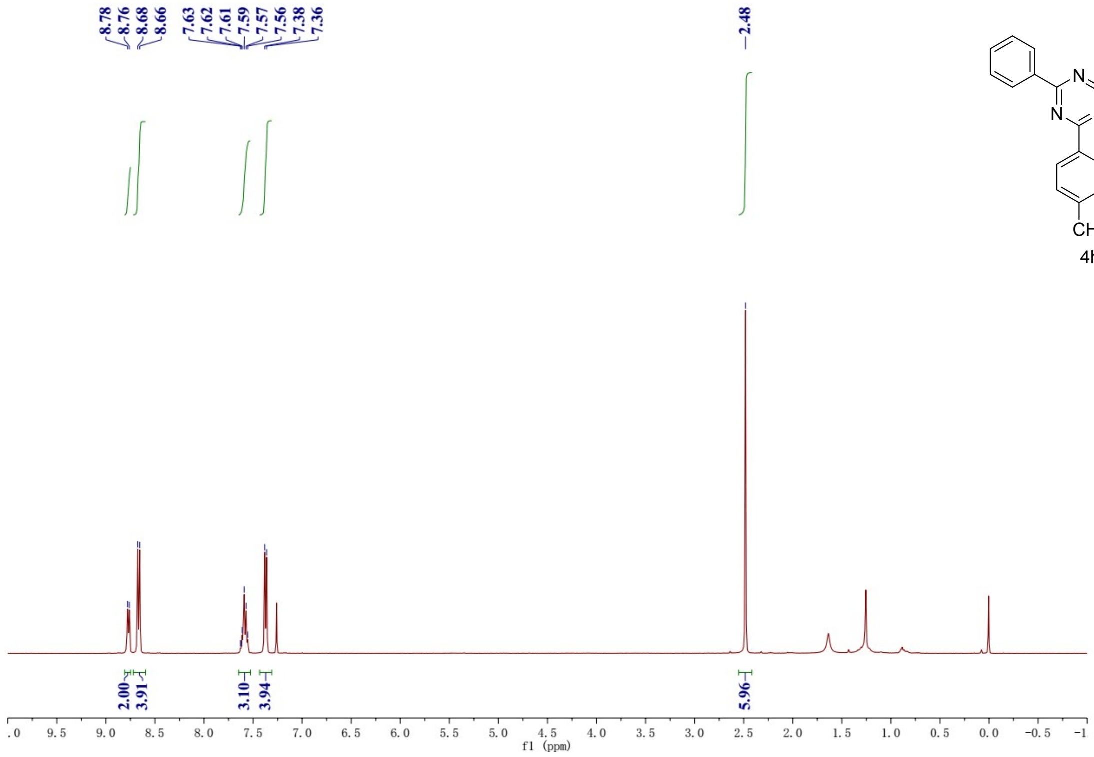
8.78
8.76
8.68
8.66
7.63
7.62
7.61
7.59
7.57
7.56
7.38
7.36
2.48
2.00
3.91
3.10
3.94
5.96
9.5 9.0 8.5 8.0 7.5 7.0 6.5 6.0 5.5 5.0 4.5 4.0 3.5 3.0 2.5 2.0 1.5 1.0 0.5 0.0 -0.5 -1
f1 (ppm)

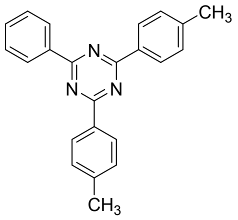
CH3
N
N
N
CH3
4h

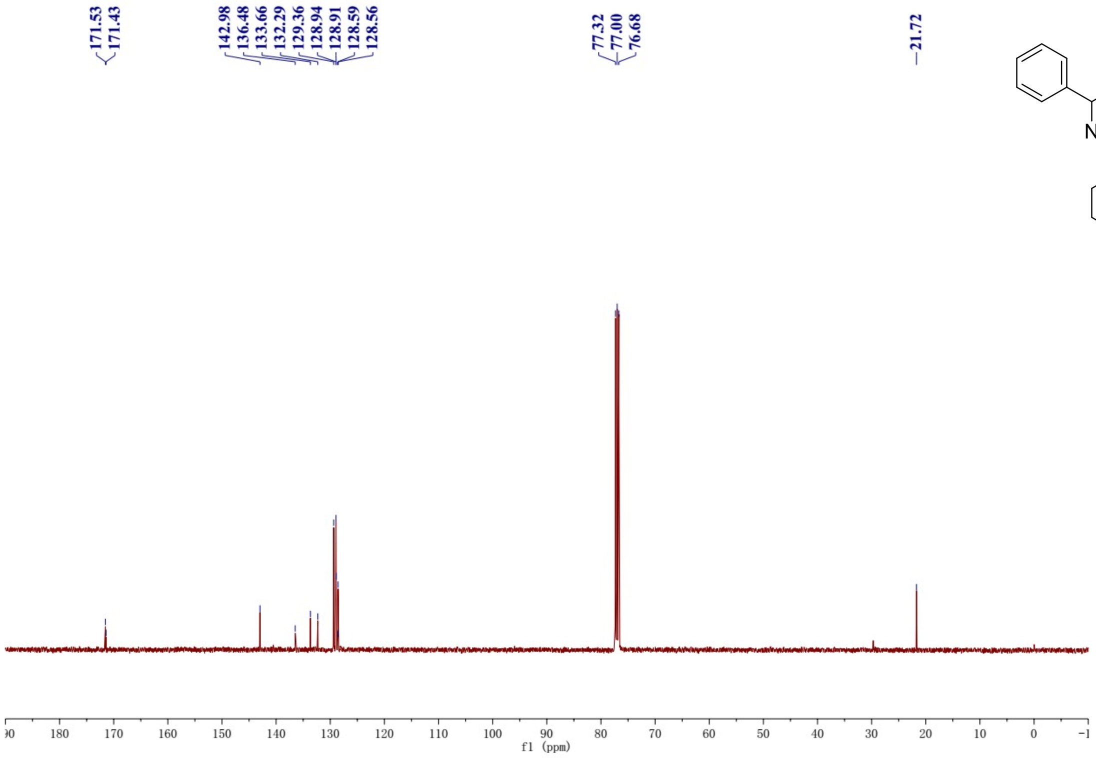
171.53
171.43
142.98
136.48
133.66
132.29
129.36
128.94
128.91
128.59
128.56
77.32
77.00
76.68
21.72
f1 (ppm)

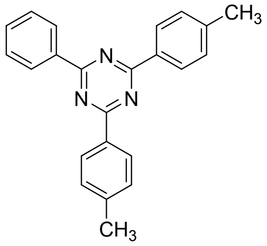
N
N
N
CH3
CH3

4h

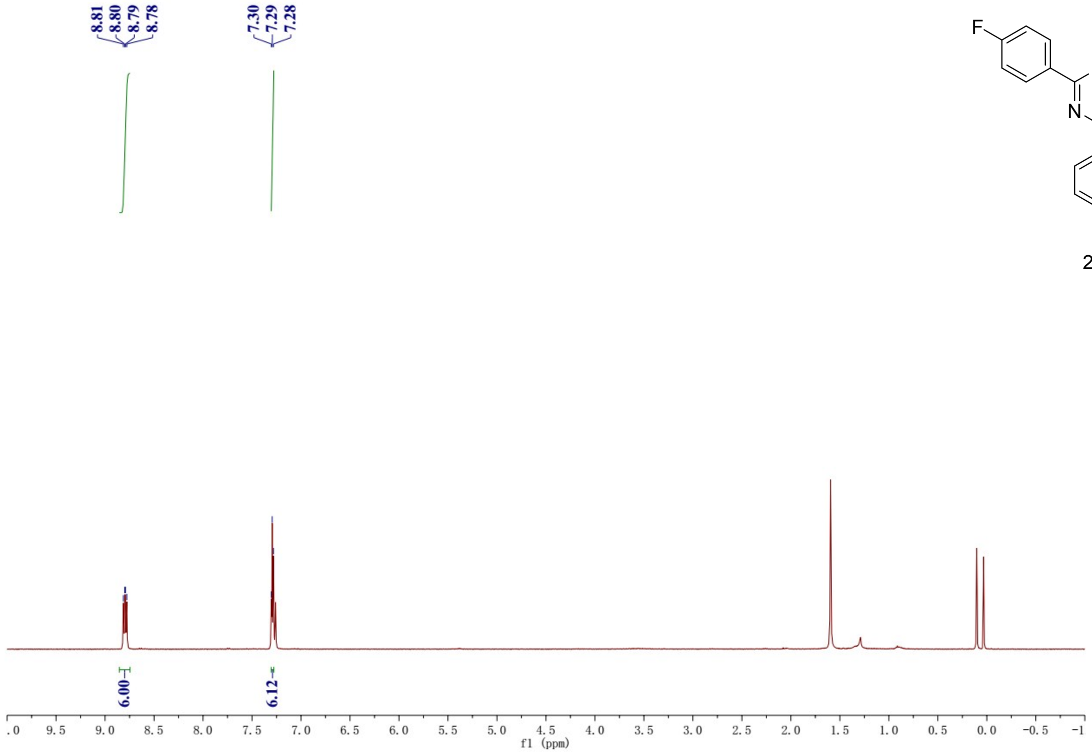
8.81
8.80
8.79
8.78
7.30
7.29
7.28
6.00
6.12
f1 (ppm)

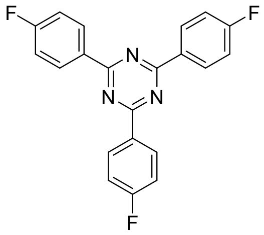
F
F
F
N
N
N

2p

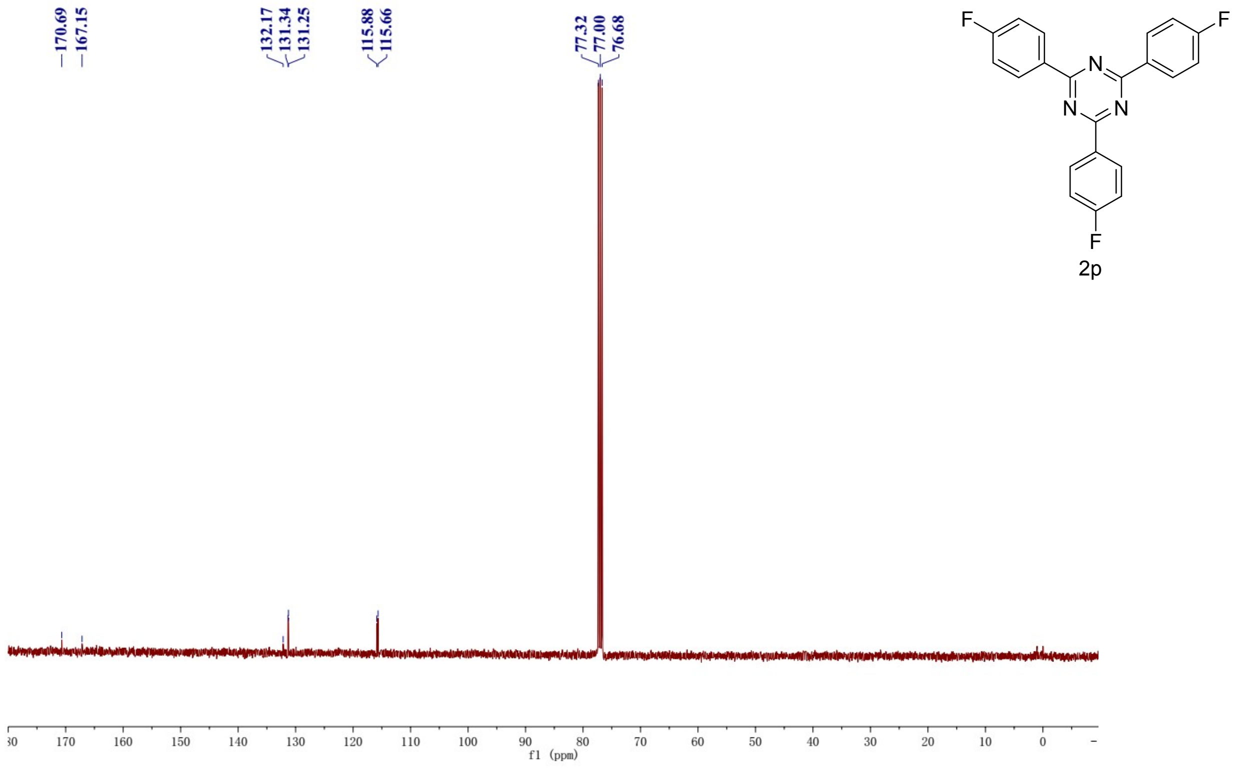

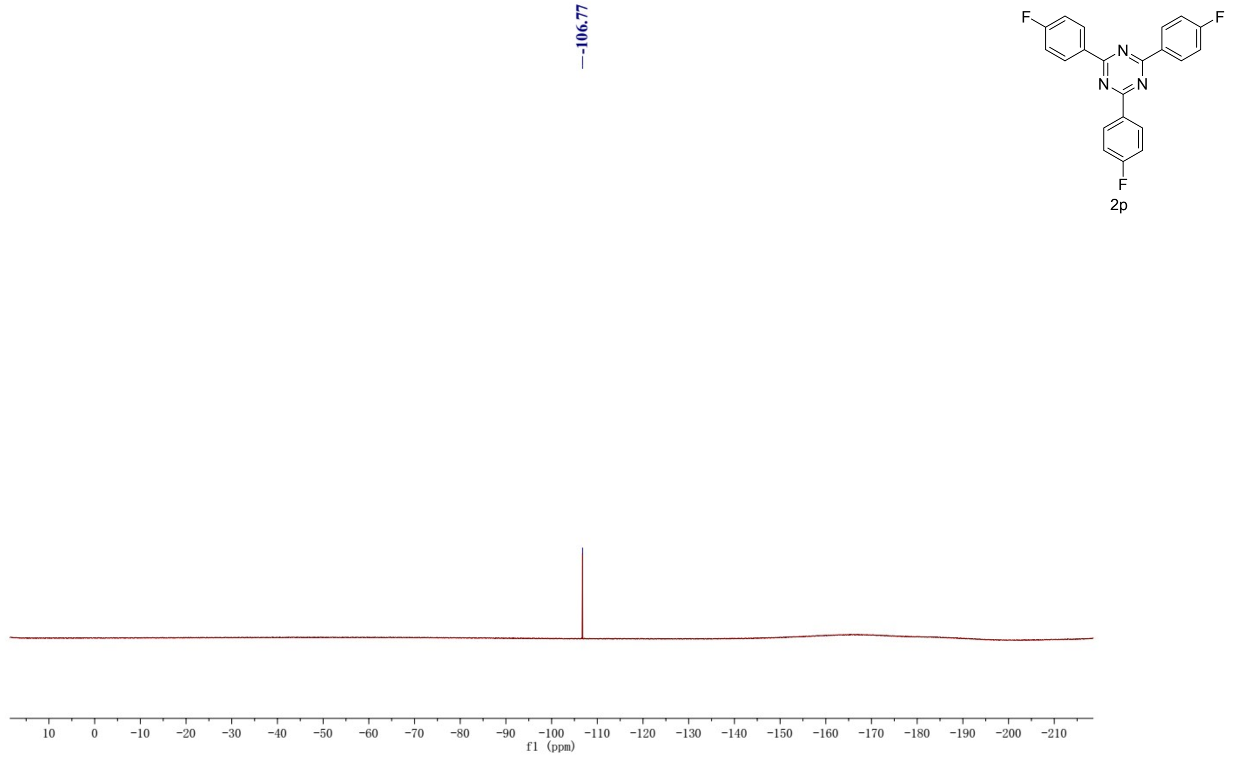

Notes and references

[1] Q. You, F. Wang, C. Wu, T. Shi, D. Min, H. Chen, W, Zhang, *Org. Biomol. Chem.,* 2015, **13**, 6723–6727.

[2] Y. Li, Z. Sun, *Heterocycles,* 2005, **65**, 1425-1430.

[3] A. Naka, R. Fukuda, R. Kishimoto, Y. Yamashita, Y. Ooyama, J. Ohshita, M. Ishikawa, *Journal*

of Organometallic Chemistry, 2012, **702**, 67-72.
[4] A. Isfahani, I. Mohammadpoor-Baltork, V. Mirkhani, A. R. Khosropour, M. Moghadam, S. Tangestaninejad, R. Kia, *Adv. Synth. Catal*., 2013, **355**, 957-972.
[5] Y. Zu, G. Li, L. Zong, L. Qing, J. Wang, X. Jian, *Polym Int ,*2018, **67**, 189–196.
[6] W. Guo, *Org. Biomol. Chem*., 2015, **13**, 10285-10289.
[7] S. D. Pardeshi, P. A. Sathe, B. V. Pawar, K. S. Vadagaonkar, A. C. Chaska, *Eur. J. Org. Chem.* 2018, **18**, 2098–2102.
[8] A. R. Tiwari, S. R. Nath, K. A. Joshi, B. M. Bhanage, *J. Org. Chem.* 2017, **82**, 13239-13249.
[9] A. Herrera, A. Riano, R. Martínez-Alvarez, *J. Org. Chem*., 2014, **79**, 7012-7024.
[10] H. Yoshida, Y. Mimura, J. Ohshita, *Chemistry letters,* 2009, **38**, 1132-1133
